# Reactive Noble-Gas Compounds Explored by 3D Electron Diffraction: XeF_2_–MnF_4_ Adducts and a Facile Sample Handling Procedure

**DOI:** 10.1021/acscentsci.4c00815

**Published:** 2024-08-14

**Authors:** Klemen Motaln, Kshitij Gurung, Petr Brázda, Anton Kokalj, Kristian Radan, Mirela Dragomir, Boris Žemva, Lukáš Palatinus, Matic Lozinšek

**Affiliations:** † Jožef Stefan Institute, Jamova cesta 39, 1000 Ljubljana, Slovenia; ‡ Jožef Stefan International Postgraduate School, Jamova cesta 39, 1000 Ljubljana, Slovenia; § Department of Structure Analysis, Institute of Physics of the Czech Academy of Sciences, Na Slovance 1999/2, Prague 8, 18221, Czech Republic

## Abstract

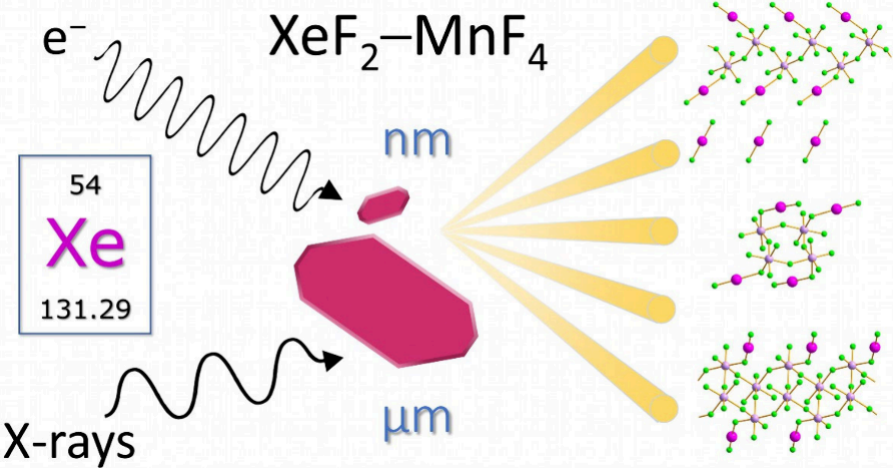

Recent advances in 3D electron diffraction (3D ED) have succeeded in matching the capabilities of single-crystal X-ray diffraction (SCXRD), while requiring only submicron crystals for successful structural investigations. One of the many diverse areas to benefit from the 3D ED structural analysis is main-group chemistry, where compounds are often poorly crystalline or single-crystal growth is challenging. A facile method for loading and transferring highly air-sensitive and strongly oxidizing samples at low temperatures to a transmission electron microscope (TEM) for 3D ED analysis was successfully developed and tested on xenon(II) compounds from the XeF_2_–MnF_4_ system. The crystal structures determined on nanometer-sized crystallites by dynamical refinement of the 3D ED data are in complete agreement with the results obtained by SCXRD on micrometer-sized crystals and by periodic density-functional theory (DFT) calculations, demonstrating the applicability of this approach for structural studies of noble-gas compounds and highly reactive species in general. The compounds 3XeF_2_·2MnF_4_, XeF_2_·MnF_4_, and XeF_2_·2MnF_4_ are rare examples of structurally fully characterized xenon difluoride–metal tetrafluoride adducts and thus advance our knowledge of the diverse structural chemistry of these systems, which also includes the hitherto poorly characterized first noble-gas compound, “XePtF_6_”.

## Abstract 2

A facile sample handling and transfer procedure enabled the crystal structure determinations of highly reactive xenon(II) compounds by 3D electron diffraction on nanometer-sized crystals.

## Introduction

Methods for structural characterization have played a pivotal role in the development of chemistry, materials science, and related fields. X-ray crystallography, particularly single-crystal X-ray diffraction (SCXRD), has assumed a central role in structural elucidation due to the wealth of information it provides about a sample.^1,2^ However, the main obstacle for the characterization by SCXRD is predominantly the unavailability of single crystals of suitable size and quality. Recently, 3D electron diffraction (3D ED) has emerged as an attractive alternative technique, which enables the crystal structure determination on nanometer-sized crystallites.^3−5^ However, the fine powdered form of the sample with a large surface area also possesses a higher risk for inadvertent reactions with both air and the grid materials. While the 3D ED analysis of air-stable and nonreactive samples is gaining prominence, only scarce attention was devoted to the study of reactive species.^6^ In this work, a facile low-temperature method for loading and transferring highly air-sensitive and strongly oxidizing samples into the TEM for 3D ED analysis was developed and tested on xenon compounds.

At present, the chemistry of xenon is the most explored and the richest of all the noble gases, with more than 150 reported crystal structures of xenon-containing compounds. However, the first noble-gas compound, “XePtF_6_”, remains a notable exception. It was first synthesized in 1962 in a landmark experiment by Neil Bartlett that finally disproved the dogmatic assumption that noble gases cannot form true chemical compounds.^7,8^ Although discovered more than 60 years ago, the first noble-gas compound remains poorly structurally characterized, and in the reinvestigation of the reaction of Xe with PtF_6_, it has been proposed that the product formed is a mixture of Xe^II^Pt^IV^F_6_, [Xe^II^F][Pt^V^F_6_], and PtF_5_, the ratio depending on the reactant stoichiometry.^9−13^ Although the crystal structure of “XePtF_6_” was not elucidated, it has been suggested that it may be XeF_2_·PtF_4_ adduct containing the [PtF_5_]^−^ anion, which might take the form of either an infinite polymer or a discrete cyclic oligomer.^10,11^

To gain insight into the structural characteristics of “XePtF_6_”, investigations of analogous systems were carried out, revealing the existence of several other *a*XeF_2_–*b*MF_4_ adducts^14^ with M = Ti,^15−17^ Cr,^15,18−20^ Mn,^15,21−23^ Rh,^9^ Pd,^10^ Sn,^24^ and Pt.^9−11^ In particular, a close structural relation of XeF_2_·PtF_4_ (“XePtF_6_”) and XeF_2_·2PtF_4_ adducts to the corresponding palladium analogs was established.^10,11^ However, structural characterization of the majority of these compounds was again hampered by their amorphous or poorly crystalline nature and the challenges associated with crystal growth, so that only four crystal structures have been reported to date, namely XeF_2_·CrF_4_,^19^ XeF_2_·2CrF_4_,^20^ [Xe_2_F_3_][Ti_8_F_33_] (2XeF_2_·8TiF_4_), and [XeF]_2_[Ti_9_F_38_] (2XeF_2_·9TiF_4_).^17^ Particularly noteworthy is the structure of XeF_2_·CrF_4_, which consists of [CrF_6_] octahedra, each with coordinated XeF_2_ unit and connected by *trans*-vertices to form polymeric chains, and thus represents a structural model for “XePtF_6_”.^11^ The crystallographically characterized XeF_2_–MF_4_ compounds are structurally diverse and exhibit different degrees of XeF_2_ ionization.^25^ Previous published work on the XeF_2_–MnF_4_ system reported the syntheses of XeF_2_·MnF_4_ and XeF_2_·2MnF_4_ and the measurements of their infrared spectra and magnetic susceptibility.^21,22^ Despite the incomplete structural characterization, XeF_2_·MnF_4_ was clearly established as a Mn^IV^ compound by the magnetic susceptibility measurements, thus marking it as a potential analog of “XePtF_6_”.

In this work, the XeF_2_–MnF_4_ system was re-examined. The adducts 3XeF_2_·2MnF_4_, XeF_2_·MnF_4_, and XeF_2_·2MnF_4_ have been synthesized and fully characterized. The structural determination of these strongly oxidizing and highly air-sensitive compounds was performed by 3D ED, and the results were benchmarked against SCXRD data and periodic DFT calculations. This work represents the first case of structural elucidation of noble-gas compounds by 3D ED. The successful transfer of the highly reactive and air-sensitive noble-gas compounds into a transmission electron microscope (TEM) and 3D ED analysis demonstrates the feasibility and broad applicability of structural determination of reactive species using 3D ED. This procedure opens the path to characterizing other challenging compounds that have evaded crystal structure determination.

## Results and Discussion

### Crystal Structure Determination by 3D ED, SCXRD, and Periodic DFT Calculations

**Figure 1:**
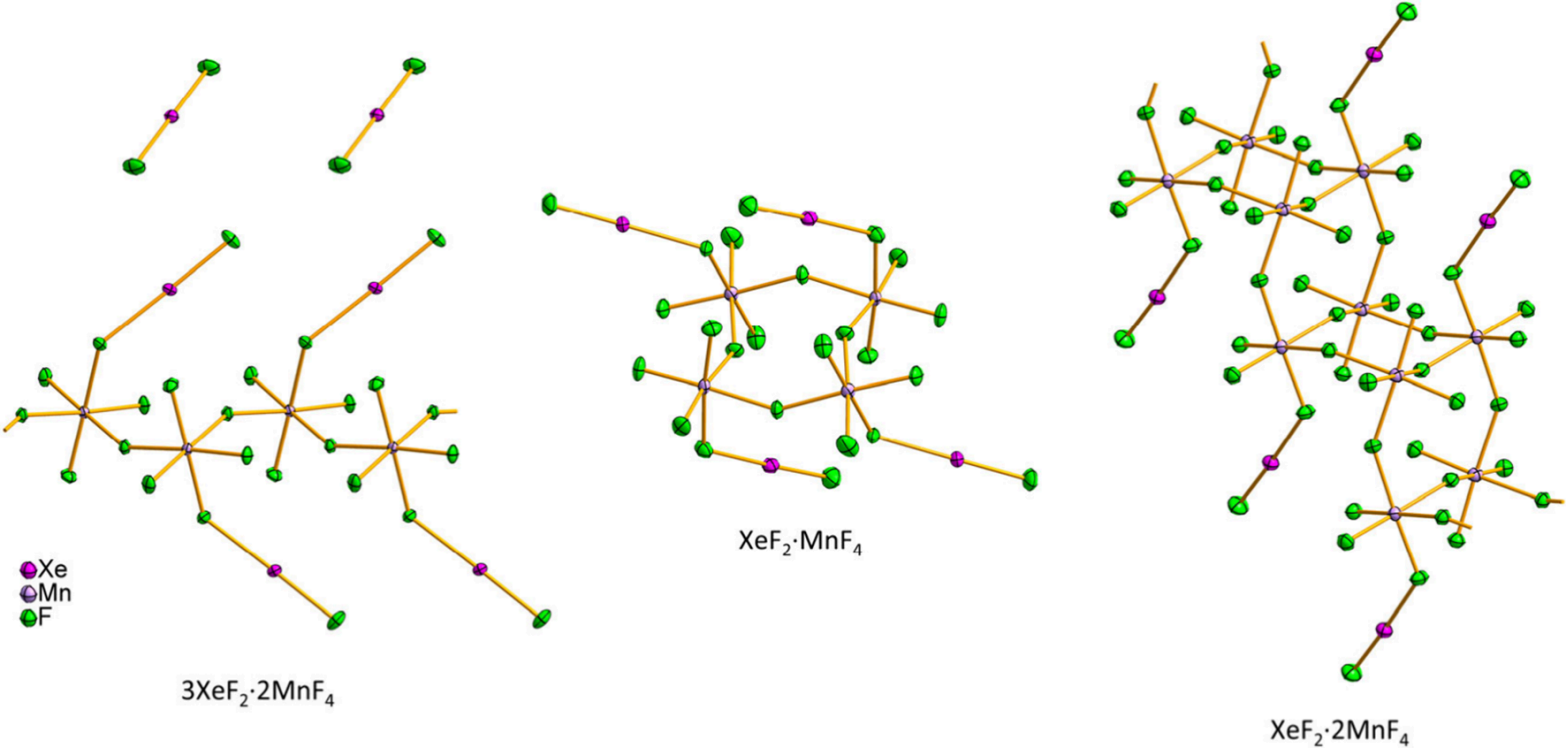
X-ray crystal structures of 3XeF_2_·2MnF_4_ (left), XeF_2_·MnF_4_ (center), and XeF_2_·2MnF_4_ (right). Thermal ellipsoids are drawn at the 50% probability level.

**Figure 2:**
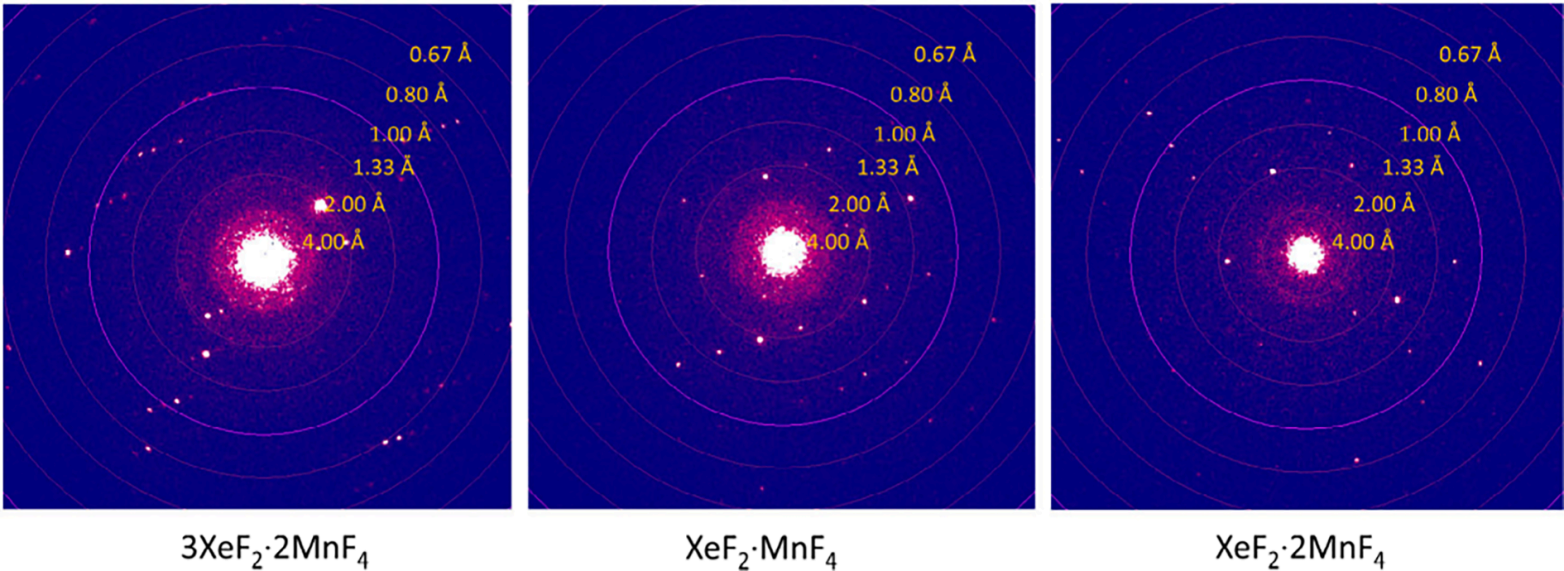
Frames at the beginning of the 3D ED data collection containing reflections to the highest resolution. For 3XeF_2_·2MnF_4_ and XeF_2_·2MnF_4_, the maximum resolution was consistently high (*d*_min_ = 0.71 Å) throughout the data collection.

The crystal structures of all three XeF_2_–MnF_4_ adducts were determined using 3D ED data and SCXRD (Figures 1, S1–S3; Tables 1, S1–S4). Despite the strongly oxidizing nature of Xe^II^ and Mn^IV^ (MnF_4_ is the highest binary fluoride of manganese),^26^ the compounds 3XeF_2_·2MnF_4_ and XeF_2_·2MnF_4_ proved to be surprisingly stable under the electron beam and produced high-resolution diffraction patterns (*d*_min_ = 0.71 Å) throughout the entire tilt range (Figure 2, Tables S1, S3). However, this was not the case for XeF_2_·MnF_4_, as its crystals decomposed after only a few seconds of electron beam irradiation, resulting in a significant decrease in the diffraction resolution. To address this issue, diffraction patterns from different crystals were collected at various tilt angles (Table S2) and merged to obtain complete information for the structure solution. The same merging process was performed for the 3XeF_2_·2MnF_4_ to obtain more complete data (Table S1). The structures were initially refined kinematically (Tables S1–S3), ignoring the contribution of dynamical effects to the diffracted intensities. Further refinement of the structures by accounting for multiple scattering using dynamical refinement theory^27,28^ led to much lower figures of merit and more precise lattice parameters and bond lengths (Tables 1 and 2).

**Table 1:** Crystal Data and Refinement Results for 3XeF_2_·2MnF_4_, XeF_2_·MnF_4_, and XeF_2_·2MnF_4_^a^

Compound	3XeF_2_·2MnF_4_			XeF_2_·MnF_4_			XeF_2_·2MnF_4_		
Method	SCXRD	3D ED	DFT/PBE-D	SCXRD	3D ED	DFT/PBE-D	SCXRD	3D ED	DFT/PBE-D
Space group	*P*2_1_/*n*			*P*2_1_/*n*			*P*2_1_/*n*		
*a* (Å)	10.24504(11)	10.2668(19)	10.134	9.6430(5)	9.6505(9)	9.635	5.18640(8)	5.2046(4)	5.213
*b* (Å)	4.99654(5)	5.0036(3)	4.982	10.9859(4)	11.108(2)	10.976	9.88546(13)	9.9679(8)	9.715
*c* (Å)	12.44149(14)	12.4668(9)	12.439	9.7927(3)	9.7900(11)	9.570	14.6809(2)	14.6946(18)	14.534
β (deg)	96.5287(10)	96.551(11)	95.8	96.979(4)	96.858(8)	96.28	96.8660(14)	96.789(6)	96.47
*V* (Å^3^)	632.747(12)	636.25(13)	624.76	1029.72(7)	1042.0(3)	1006.05	747.292(19)	756.99(13)	731.44
*Z*	2			8			4		
*M*_r_ (g mol^–1^)	769.78			300.24			431.18		
*D*_calcd_ (g cm^–3^)	4.040	4.017		3.873	3.827		3.832	3.783	
*T* (K)	100	100		100	100		100	100	
*R*_1_	0.0226	0.1320		0.0378	0.1180		0.0350	0.0770	
*wR*_2_**/***wR*_all_	0.0600	0.1480		0.0864	0.1326		0.0775	0.0848	
Δρ_max_, Δρ_min_ (e Å^–3^) **/**Δ*V*_max_, Δ*V*_min_ (e Å^–1^)	1.577, −1.956	1.96, −1.94		6.666, −1.608	0.91, −0.83		2.015, −1.399	0.68, −0.63	

a Experimental determinations were performed at a temperature of 100 K.

**Table 2:** Selected Bond Lengths (Å) and Angles (deg) in the Crystal Structures of 3XeF_2_·2MnF_4_, XeF_2_·MnF_4_, and XeF_2_·2MnF_4_^a^

	3XeF_2_·2MnF_4_			XeF_2_·MnF_4_			XeF_2_·2MnF_4_		
	SCXRD	3D ED	DFT/PBE-D	SCXRD	3D ED	DFT/PBE-D	SCXRD	3D ED	DFT/PBE-D
Xe–F_t_	1.9204(7), 1.9933(7)^b^	1.900(8), 1.985(8)^b^	1.981, 2.042^b^	1.906(3), 1.910(3)	1.917(18), 1.922(13)	1.973, 1.976	1.8946(18)	1.877(9)	1.950
Xe–F_b_	2.1695(6)	2.176(6)	2.181	2.176(3), 2.180(3)	2.189(15), 2.200(14)	2.193, 2.198	2.2829(16)	2.286(7)	2.266
Mn–F_b_(Xe)	1.9133(6)	1.896(9)	1.953	1.902(3), 1.928(3)	1.887(17), 1.960(17)	1.940, 1.984	1.8181(16)	1.823(5)	1.865
Mn–F_b_(Mn)	1.9011(6), 1.9058(6)	1.893(6), 1.908(5)	1.915, 1.920	1.887(3)–1.903(3)	1.862(12)–1.929(12)	1.910–1.925	1.8499(15)–1.9330(16)	1.872(5)–1.921(6)	1.891–1.948
Mn–F_t_	1.7264(6)–1.7358(8)	1.686(9)–1.728(6)	1.745–1.752	1.720(3)–1.741(3)	1.65(2)–1.79(2)	1.744–1.753	1.7253(17)–1.7340(16)	1.713(5)–1.752(6)	1.742–1.749
Mn–F–Mn	149.60(4)	150.4(4)	146.0	147.93(17), 149.24(17)	146.0(9), 147.7(9)	143.4, 143.9	138.81(9)–147.19(9)	138.7(4)–148.1(3)	133.5–142.6
*cis*-F–Mn–F	85.08(3)–94.31(4)	85.6(3)–94.4(3)	83.4–95.3	82.96(13)–94.42(15)	82.7(6)–98.0(9)	82.8–95.4	86.47(7)–93.39(8)	85.9(2)–93.4(3)	86.6–94.5
*trans-*F–Mn–F	174.13(3)–176.14(3)	174.3(3)–176.4(3)	172.6–175.2	172.62(15)–175.23(15)	168.9(9)–175.3(7)	171.6–174.3	173.99(8)–177.71(8)	174.1(3)–177.0(3)	172.9–178.4
F–Xe–F	177.47(3), 180.00(5)^b^	177.0(3), 180^b^	176.7, 180.0^b^	177.48(14), 178.52(14)	178.2(7), 179.2(6)	176.9, 177.6	176.84(8)	176.8(2)	177.6

a F_t_ and F_b_ denote the terminal and bridging fluorine atoms, respectively.

b Cocrystallized centrosymmetric XeF_2_ molecule in 3XeF_2_·2MnF_4_.

**Figure 3:**
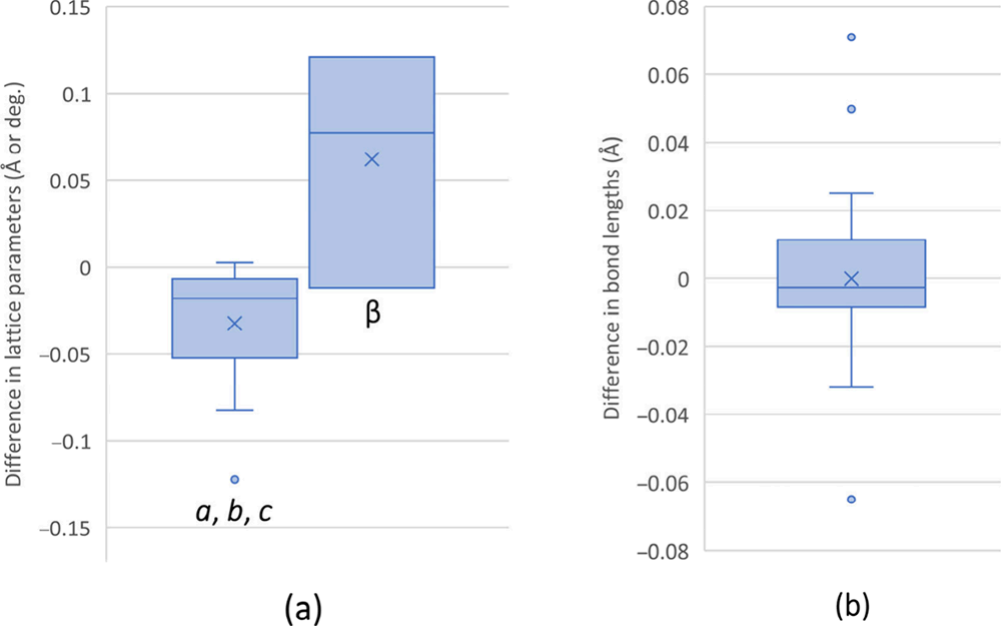
Box and whisker plots showing the difference in (a) lattice parameters and (b) bond lengths of the three XeF_2_–MnF_4_ compounds between SCXRD and 3D ED refinement results. The differences were calculated as the value of the parameter obtained from SCXRD minus the value of the parameter determined by 3D ED. The average difference is marked with a symbol × and the outliers are shown as circles.

The values of the agreement parameter *R*_1_ for the 3D ED structures are considerably higher than their SCXRD counterparts (Table 1), but these values are nevertheless good for 3D ED.^29^ The lattice parameters determined from 3D ED are generally believed to be of very low accuracy, and compared to the unit cell parameters obtained from SCXRD, there is a deviation of up to −1.1%, with a maximum difference amounting to −0.122 Å/0.12° and an average difference of −0.03 Å/0.06° (Figure 3a). These discrepancies are comparable to differences observed when comparing lattice parameters from SCXRD and values calculated by DFT. The PBE-D functional slightly underestimates lattice parameters and volumes, with deviations ranging from −0.5% to +2.3%. Moreover, comparing the unit cell parameters, which were obtained in several independent SCXRD structure determinations, differences of up to −0.4% are observed (Table S4). This indicates that the systematic differences in the values of the unit cell parameters obtained by 3D ED when compared to SCXRD and DFT/PBE-D are reasonably small. Such a good agreement could be obtained owing to the careful determination of the lattice parameters from 3D ED using the program *PETS2*,^30^ and employing the refinement of the optical distortions along with the refinement of the lattice parameters.^31^ It is also evident that 3D ED lattice parameters are, on average, slightly larger than their counterparts derived from SCXRD (Table 1). This difference, and consequently, the higher unit cell volumes of all structures determined by 3D ED compared to SCXRD results suggest that the deviation of the 3D ED results might be due to the radiation damage of the samples exerted by the electron beam. The unit-cell volume expansion is a frequently observed global radiation damage effect.^32,33^

A comparison of bond lengths and angles of the elucidated crystal structures and DFT calculations (Table 2, Figure 3b) gives a better understanding of the accuracy of the results. The bond parameters derived from the two methods are in very good agreement. Typically, the differences are not significant (88.4% of all bond distances and angles are within the “3σ rule”; Tables S5–S7) and are within the range of ±0.08 Å and ±5°. The terminal Mn–F_t_ bond lengths showed the largest discrepancies (exceeding the 3σ threshold) between the bond lengths determined by SCXRD and 3D ED. In the crystal structures of 3XeF_2_·2MnF_4_ and XeF_2_·MnF_4_ determined by 3D ED, one of the Mn–F_t_ bonds was significantly shorter than the corresponding bonds in structures determined by SCXRD (1.686(9) Å vs 1.7358(8) Å in 3XeF_2_·2MnF_4_; 1.65(2) Å vs 1.721(3) Å in XeF_2_·MnF_4_). Similarly, in the 3D ED structures of XeF_2_·MnF_4_ and XeF_2_·2MnF_4_, one of the Mn–F_t_ bonds is elongated compared to the SCXRD results (1.79(2) Å vs 1.725(3) Å in XeF_2_·MnF_4_; 1.752(6) Å vs 1.7282(16) Å in XeF_2_·2MnF_4_). Significant discrepancies between the 3D ED and SCXRD results are also observed in the Mn–F_b_(Mn) bond lengths in the crystal structure of XeF_2_·2MnF_4_, where a shift in the position of the fluorine atom F5 leads to the elongation of one bond (1.872(5) Å vs 1.8499(15) Å) and shortening of the other Mn–F_b_(Mn) bond (1.909(6) Å vs 1.9330(16) Å). Similar to the observed increase in lattice parameters and unit cell volume, the most likely reason for the differences in bond distances is the effect of site-specific radiation damage, which is typically manifested in particular fragments or sensitive functional groups.^32,33^ In spite of these small differences, the overall results are in good agreement and validate each other. As the results from X-ray diffraction have better figures of merit and, in general, smaller standard uncertainties, the structure parameters from SCXRD are discussed in the subsequent description of the structures, with the note that the same overall picture and conclusions are obtained also on the basis of the 3D ED data.

**Figure 4:**
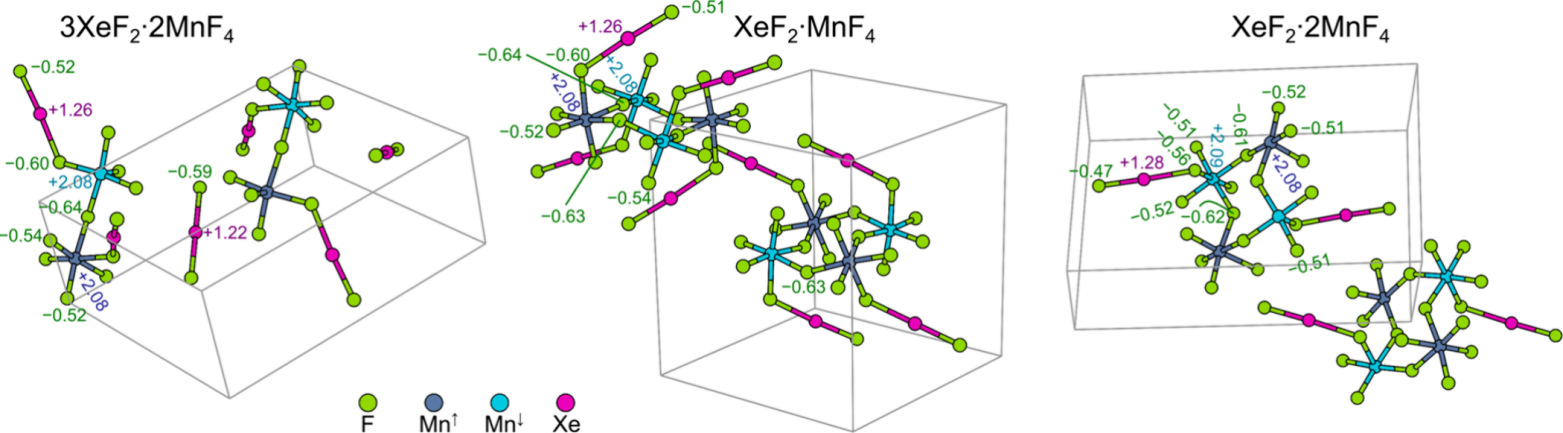
DFT-modeled crystal structures of 3XeF_2_·2MnF_4_, XeF_2_·MnF_4_, and XeF_2_·2MnF_4_, with the utilized antiferromagnetic arrangements shown by using different colors for the spin-up (Mn^↑^) and spin-down (Mn^↓^) polarized Mn ions. Calculated atomic Bader charges are also stated (for comparison, the Bader charges of Xe and F ions in the XeF_2_ crystal are +1.21 and −0.61, respectively).

Crystal structures were modeled with DFT as antiferromagnetic, resulting in zero total magnetization (the antiferromagnetic arrangements of the Mn ions within the unit cell of the three considered crystal structures are depicted in Figure 4). However, the absolute magnetization is about 3 μ_B_/Mn-ion for all three crystal structures (Table 3), in fair agreement with experimental magnetic measurements.^21,22^ These values are consistent with a 3d^3^ valence electronic configuration, confirming the +4 oxidation state of Mn. In contrast, the calculated Bader charges of Mn ions are considerably smaller than +4, being around +2.1 (Table 3 and Figure 4). This finding is not surprising because atomic charge is not synonymous with the formal oxidation state. It is known that even ions that are formally without valence electrons, such as V^V^, display a substantial valence electron density.^34^ Bader charges of Xe are about +1.3 and those of F atoms range from about −0.5 to −0.6, consistent with the formal oxidation states of +2 and −1 for xenon and fluorine, respectively. These values are similar to the calculated Bader charges of Xe and F atoms in the XeF_2_ crystal, +1.21 and −0.61, respectively.

### Description of the Crystal Structures

#### 3XeF_2_·2MnF_4_

The crystal structure of 3XeF_2_·2MnF_4_ consists of polymeric chains of *cis-*F-bridged [MnF_6_] octahedra with the apically coordinated XeF_2_ molecules (Figure 1) alternating above and below the chain, which can be described by the crystal coordination formula^35^_∞_^1^[MnF_3_F_2/2_(XeF_2_)]. The manganese bridging F atoms lie alternating above and below the plane of metal atoms, thus the chain could also be described as Mn–F–Mn–F–Mn helix, which propagates parallel to the *b*-crystallographic axis (Figure S4). The bond lengths of the terminal F atoms (Mn–F_t_ 1.7264(6)–1.7358(8) Å) are considerably shorter than the bond lengths of the bridging F atoms (Mn–F_b_(Mn) 1.9011(6), 1.9058(6) Å), with the longest bond involving the XeF_2_ ligand (Mn–F_b_(Xe) 1.9133(6) Å). The voids between adjacent chains are occupied by cocrystallized XeF_2_ molecules. The molecular geometry of the cocrystallized XeF_2_ (1.9933(7) Å, 180.00(5)°) is essentially the same as that observed in the crystal structure of pure XeF_2_.^36^ On the other hand, the XeF_2_ ligand coordinated to the Mn center is significantly distorted, with shortened Xe–F_t_ (1.9204(7) Å) and elongated Xe–F_b_ (2.1695(6) Å) bonds. A similar helical polymeric *cis*-chain was observed in the crystal structure of H_3_O^+^[TiF_5_]^−^.^37^

#### XeF_2_·MnF_4_

In contrast to the polymeric structure of 3XeF_2_·2MnF_4_, the building unit of XeF_2_·MnF_4_ crystal structure is a discrete F-bridged cyclic tetramer, _∞_^0^[⟨MnF_3_F_2/2_(XeF_2_)⟩_4_^r^] (Figure 1). The three bridging F atoms of [MnF_6_] octahedron form a *fac* geometry, with XeF_2_ ligand coordinated apically. A pair of ligands is situated above a centrosymmetric ring, and the other pair is below it (Figure S5). The Mn–F_t_ (1.720(3)–1.741(3) Å), Mn–F_b_(Mn) (1.887(3)–1.903(3) Å), and Mn–F_b_(Xe) (1.902(3), 1.928(3) Å) bond distances are comparable to those observed in 3XeF_2_·2MnF_4_, as is the distortion of the XeF_2_ ligand (Xe**–**F_t_ 1.906(3), 1.910(3) Å; Xe–F_b_ 2.176(3), 2.180(3) Å). Interestingly, such a tetrameric ring motif, based on the RhF_5_ tetramer,^38^ has been proposed earlier for the crystal structures of both “XePtF_6_” (XeF_2_·PtF_4_) and XeF_2_·PdF_4_.^10^ The other rare crystallographically characterized examples of anionic M_4_F_20_ rings known to date include the Ti_4_F_20_^4–^ and Ge_4_F_20_^4–^ anions.^39,40^

#### XeF_2_·2MnF_4_

The asymmetric unit of XeF_2_·2MnF_4_ consists of two crystallographically independent [MnF_6_] units, one of which is apically coordinated by XeF_2_. Each [MnF_6_] unit is connected to three neighboring units into a polymeric double chain, _∞_^1^[⟨MnF_2_F_3/1+1_(XeF_2_)⟩⟨MnF_3_F_3/1+1_⟩], so that each XeF_2_-bearing octahedron is connected to three regular [MnF_6_] units and vice versa. The double chain has the form of a corrugated ladder or a staircase-like shape (Figure S6). The Mn–F_t_ bond lengths (1.7253(17)–1.7340(16) Å) are comparable to those observed in the two aforementioned adducts, whereas Mn–F_b_(Mn) distances (1.8499(15)–1.9330(16) Å) span a larger range, and Mn–F_b_(Xe) (1.8181(16) Å) is the shortest bridging bond. The latter is in stark contrast with the structures of 3XeF_2_·2MnF_4_ and XeF_2_·MnF_4_, where the Mn–F bonds involving the XeF_2_ ligand are the longest. Similarly, the distortion of XeF_2_ ligand (Xe**–**F_t_ 1.8946(18) Å; Xe–F_b_ 2.2829(16) Å) is much larger and comparable to the XeF^+^ salts (e.g., [XeF]_2_[Ti_9_F_38_],^17^ [XeF][AF_6_], A = As, Sb, Bi).^36^ Therefore, the XeF_2_·2MnF_4_ compound could be considered a XeF^+^ salt or tight ion pair, [XeF]^+^[Mn_2_F_9_]^−^. An anion with a similar staircase-like double chain structure is [Ti_2_F_9_]^−^,^39,41^ whereas the geometry of the double chain anion in the crystal structure of [O_2_]^+^[Mn_2_F_9_]^−^ is different.^42^

**Table 3:** PBE-D Calculated Absolute Magnetizations (Normalized Per Mn Ion) and Atomic Bader Charges of 3XeF_2_·2MnF_4_, XeF_2_·MnF_4_, and XeF_2_·2MnF_4_^a^

Compound	Abs. mag.^b^ (μ_B_/Mn-ion)	Bader charge of Mn^↑^, Mn^↓^	Bader charge of Xe	Bader charge of F(Mn)	Bader charge of F_t_(Xe), F_b_(Xe), F(Xe)^d^	Charge on XeF_2_
XeF_2_	-	-	1.21	-	–0.605	0
3XeF_2_·2MnF_4_	3.15	2.08, 2.08	1.26,^c^ 1.22^d^	from −0.52 to −0.64	–0.52, −0.60, −0.59	0.14,^c^ 0.04^d^
XeF_2_·MnF_4_	3.15	2.08, 2.08	1.26	from −0.52 to −0.64	–0.51, −0.60	0.15
XeF_2_·2MnF_4_	3.12	2.08, 2.09	1.28	from –0.51 to −0.62	–0.47, −0.56	0.25

a Note that all structures have zero total magnetization, i.e., the modeled structures are perfectly antiferromagnetic. For the illustration of the antiferromagnetic arrangement of the Mn ions within the unit cell and atomic Bader charges, see Figure 4.

b Calculated as *M*_abs_ = 1/*N*_Mn_ ∫_cell_ |*n*^↑^(***r***) – *n*^↓^(***r***)| d***r***, where *N*_Mn_ is the number of Mn ions in the unit cell and *n*^↑^(***r***) and *n*^↓^(***r***) are the spin-up and spin-down electron number densities.

c XeF_2_ coordinated to Mn as F–Xe–F–Mn.

d Cocrystallized XeF_2_.

Xenon atoms in all three crystal structures exhibit a number of nonbonded Xe···F contacts shorter than the sum of van der Waals radii (Figures S7–S9; Tables S8–S10).

### Ionization of XeF_2_

**Figure 5:**
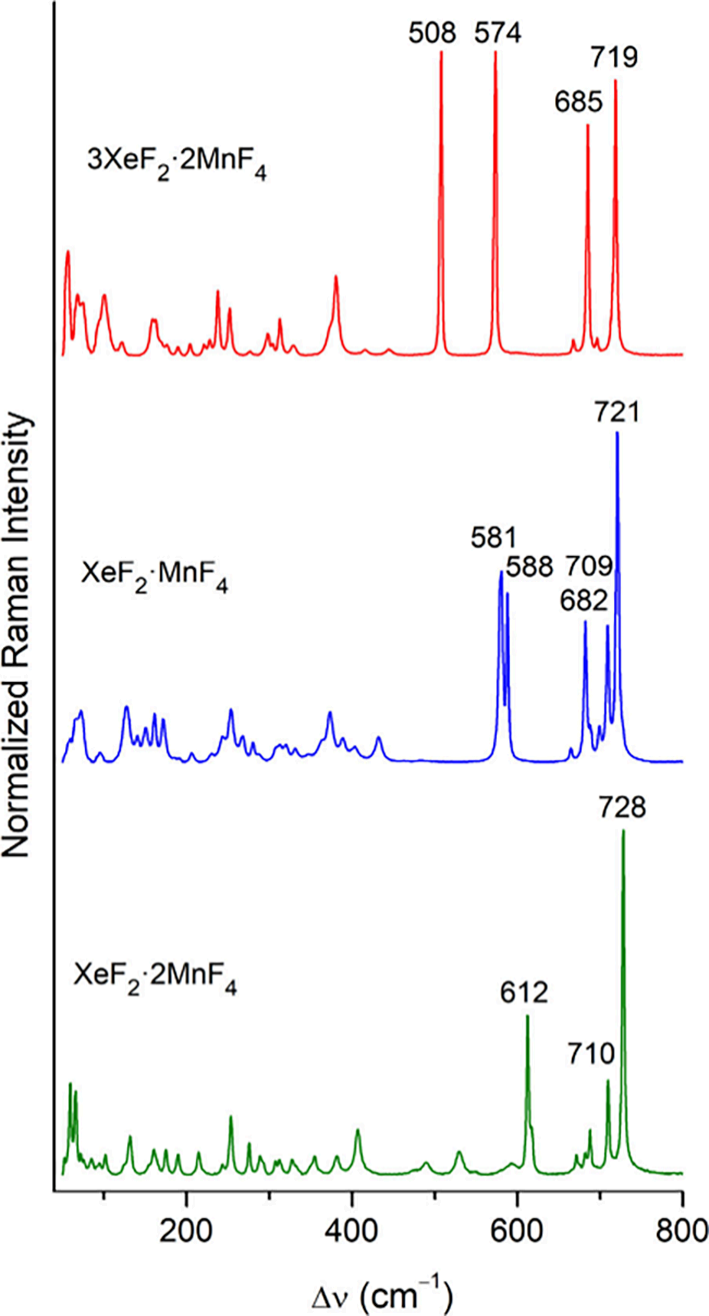
Raman spectra of powdered samples of 3XeF_2_·2MnF_4_, XeF_2_·MnF_4_, and XeF_2_·2MnF_4_ recorded at −150 °C using 785 nm excitation.

Raman spectroscopy is an effective tool for compound identification and monitoring the ionization of the bound XeF_2_, along the ionization pathway XeF_2_ → XeF^+^ + F^–^,^25^ in its Lewis acid–base adducts. As observed for fluoridotitanates(IV),^17,43^ the dimensionality of the anion affects the position of the strong symmetric in-phase M–F_t_ stretching mode. For 3XeF_2_·2MnF_4_ and XeF_2_·MnF_4_, this band is located at 719 and 721 cm^–1^, respectively, whereas for XeF_2_·2MnF_4_ the mode is observed at 728 cm^–1^ (Figures 5, S10; Table S11). The symmetric stretching vibration of cocrystallized XeF_2_ at 508 cm^–1^ observed in the Raman spectrum of 3XeF_2_·2MnF_4_ is only slightly shifted from the position of the band at 497 cm^–1^ in pure XeF_2_.^44^ The strong Xe–F_t_ stretching band of coordinated XeF_2_ shifts to higher wavenumbers with increasing stoichiometry of MnF_4_ and thus increasing Lewis-acid strength, with observed positions of 574 cm^–1^ in 3XeF_2_·2MnF_4_, 581, 588 cm^–1^ in XeF_2_·MnF_4_ and 612 cm^–1^ in XeF_2_·2MnF_4_. In the reported Raman spectra of XeF_2_·PdF_4_ and XeF_2_·PtF_4_, bands that could be attributed to the Xe–F_t_ stretch are observed at 585 and 591 cm^–1^, respectively,^10,11^ suggesting that these compounds exhibit a similar degree of XeF_2_ ionization as XeF_2_·MnF_4_. The position of the Xe–F_t_ band at 612 cm^–1^ in XeF_2_·2MnF_4_ is comparable to other XeF^+^ salts (e.g., 607, 611 cm^–1^ in [XeF][AsF_6_];^36^ 605, 610, 614 cm^–1^ in [XeF][Ta_2_F_11_];^45^ 612 cm^–1^ in [XeF]_2_[Ti_9_F_38_]).^17^ The Raman spectroscopy thus corroborates the high degree of XeF_2_ ionization, as inferred from the observed shortening and elongation of the Xe–F_t_ and Xe–F_b_ bonds, respectively, in the crystal structure of XeF_2_·2MnF_4_. Moreover, the trend in ionization is also confirmed by periodic DFT calculations, which show that Bader charges (Table 3, Figure 4) on Xe atom and XeF_2_ moiety are larger in XeF_2_·2MnF_4_ (+1.28, +0.25) than in 3XeF_2_·2MnF_4_ (+1.26, +0.14) and XeF_2_·MnF_4_ (+1.26, +0.15). The [XeF][Mn_2_F_9_] tight ion pair formulation is therefore justified. For the formation of XeF^+^, the strongest fluoride-ion abstracting Lewis acids, such as pentafluorides AsF_5_ and SbF_5_, are typically required. The [XeF][Mn_2_F_9_] is a rare structurally confirmed example of a highly ionized Xe^II^ species, which formed in interaction with a weaker Lewis acid and exhibits a polymeric anion. The only other examples of this kind are from the XeF_2_–TiF_4_ system: [Xe_2_F_3_][Ti_8_F_33_] and [XeF]_2_[Ti_9_F_38_].^17^

## Conclusions

**Figure 6:**
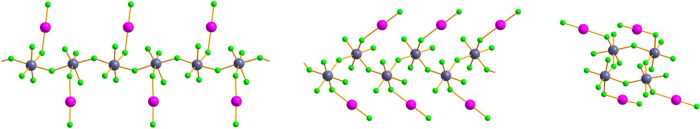
Structural models for “XePtF_6_” (XeF_2_·PtF_4_): *trans*-chain from XeF_2_·CrF_4_ (left),^19^*cis*-chain from 3XeF_2_·2MnF_4_ (center), and tetrameric ring from XeF_2_·MnF_4_ (right).

Despite considerable interest in the structural characteristics of XeF_2_–MF_4_ adducts due to their relationship with the first noble-gas compound, “XePtF_6_”, only four such compounds were crystallographically characterized and reported prior to this study.^17,19,20^ These compounds not only pose a synthetic challenge due to their air sensitivity and oxidizing nature but also present considerable difficulties in the growth of single crystals large enough for SCXRD. In limited aforementioned cases, this could be resolved only by lengthy screening for optimal crystallization conditions^17^ or recrystallization of the powdered products by prolonged exposure to a temperature gradient or the use of supercritical SF_6_.^20^ In this work, the XeF_2_–MnF_4_ system was reinvestigated, leading to the synthesis of 3XeF_2_·2MnF_4_, XeF_2_·MnF_4_, and XeF_2_·2MnF_4_, which were characterized by 3D ED, SCXRD, powder X-ray diffraction (PXRD), vibrational spectroscopy, and periodic DFT calculations. The crystal structures of 3XeF_2_·2MnF_4_ and XeF_2_·2MnF_4_ consist of infinite zigzag chains and staircase-like double chains, respectively, formed by F-bridged [MnF_6_] octahedra (Figure 1). In contrast, the crystal structure of XeF_2_·MnF_4_ consists of discrete tetrameric units and is only the second crystallographically characterized example of a Xe^II^M^IV^F_6_ compound. The structures of 3XeF_2_·2MnF_4_ and XeF_2_·MnF_4_ provide new possible structural models for the currently unknown structure of “XePtF_6_” (Figure 6). In this series of compounds, a progressive increase in the Lewis acidity of the fluoridomanganate(IV) with increasing MnF_4_ content leads to the observed progressive ionization of XeF_2_. The XeF_2_·2MnF_4_ adduct could thus be formulated as a tight ion pair [XeF]^+^[Mn_2_F_9_]^−^—a rare example of [XeF]^+^ salt with polymeric anion.

By developing a novel method for sample loading and transfer under low temperatures and inert conditions, the highly reactive noble-gas compounds could be successfully introduced into a TEM, enabling their structural determination by 3D ED. Despite comparatively poorer agreement parameters and less precise bond distances and angles, the crystal structures obtained by 3D ED are in full agreement with the SCXRD results. This study therefore demonstrates that the crystal structures of reactive, strongly oxidizing, and air-sensitive compounds can be elucidated by 3D ED analysis of nanosized crystallites, sidestepping the time-consuming single-crystal growth attempts. In conjunction with the inert sample transfer method described herein, this renders 3D ED an attractive option in the analytical toolbox for the determination of hitherto unknown structures of noble-gas compounds and other highly reactive or unstable species.

## Methods

### Syntheses

The oxidizing power of XeF_2_ near its melting point is sufficient to oxidize Mn^II^ to Mn^IV^.^21^ In the present study, MnF_2_ was treated with excess XeF_2_ in a nickel reaction vessel heated to 120 °C for 2–3 days, followed by slow cooling and removal of volatiles under dynamic vacuum at room temperature. This procedure resulted in the formation of 3XeF_2_·2MnF_4_ in the form of red needle-shaped single crystals and pink crystalline powder (Figure S11). This result contradicts the earlier reports, where a similar procedure was claimed to result in the formation of XeF_2_·MnF_4_.^21^ The single crystals obtained with this preparation proved suitable for SCXRD, while the powder was used for 3D ED. Attempts to prepare XeF_2_·MnF_4_ or XeF_2_·2MnF_4_ by oxidation of MnF_2_ with a stoichiometric amount of XeF_2_ were unsuccessful, and the Raman spectra of the obtained products indicated that 3XeF_2_·2MnF_4_ and MnF_3_ formed instead. Therefore, thermal reactions between XeF_2_ and MnF_4_ were investigated by heating the reactants in heat-sealed fluorinated ethylene-propylene (FEP) ampules, with XeF_2_ always present in a slight stoichiometric excess to compensate for the loss through the ampule walls. This procedure resulted in the formation of wine-red crystals of XeF_2_·MnF_4_ and XeF_2_·2MnF_4_, which were always covered by a large amount of a purple amorphous solid (Figure S11). The use of FEP ampules for the reactions proved advantageous, since the material that formed in the nickel reactor under the same reaction conditions contained even more of the amorphous phase. The crystals of XeF_2_·MnF_4_ and XeF_2_·2MnF_4_ prepared in FEP ampules always formed agglomerates that could not be completely separated, although some multiple crystals were found to be suitable for structural analysis by SCXRD. Another synthetic path to XeF_2_·MnF_4_ is the solvolysis of 3XeF_2_·2MnF_4_ in anhydrous HF (aHF). Upon prolonged stirring of the initially formed pink solution over the insoluble purple precipitate, pink powdery material began to deposit, replacing the dark precipitate, while the color of the solution changed to a light pink. Such preparation yielded pure microcrystalline XeF_2_·MnF_4_, however, its low solubility in aHF prevented the preparation of single crystals by recrystallization. In one of the experiments, it was found that after two cycles of adding aHF, stirring, and removing the volatiles under a dynamic vacuum, XeF_2_·2MnF_4_ also began to form, as shown by powder X-ray diffraction and Raman spectroscopy (Figure S12). The sample containing both XeF_2_·MnF_4_ and XeF_2_·2MnF_4_ was used in 3D ED studies.

### Sample Loading and Transfer for Observation in TEM

A copper TEM grid with lacey carbon was loaded onto a cryogenic TEM sample holder, which was cooled with liquid N_2_ inside a nitrogen-filled glovebox (Figures S13–S15). After the holder had cooled for about 15 min, a small amount of powdered sample was transferred onto the grid. The excess sample was removed by gently tapping the holder. The cover protecting the grid was moved over the sample, and the holder was then covered with a sleeve to protect it from the atmosphere. Sealed and cooled, the holder was then removed from the glovebox and very quickly transferred into the transmission electron microscope for data acquisition (Table S12). The sleeve was removed only immediately before inserting the holder in the TEM, while the protective cover remained in place during the whole procedure. Throughout the entire process, the maximum temperature of the holder did not exceed ∼125 K, and the exposure to air did not exceed 2 s. Despite all these measures, a significant amount of ice was usually found on the grid. However, due to low temperatures the ice did not react with the sample and did not prevent TEM observation and 3D ED data collection. Ice can be removed by briefly warming the sample to 173 K in the microscope. Detailed information on the setup for the sample loading process in the glovebox is provided in the Supporting Information.

### 3D ED, SCXRD, PXRD, Vibrational Spectroscopy, and Periodic DFT Calculations

Technical details about 3D ED, SCXRD and PXRD measurements, vibrational spectroscopy, and periodic DFT calculations are provided in the Supporting Information.

## Supporting Information Available

Detailed descriptions of experimental and computational procedures, PXRD data, additional crystallographic data (Table S13) and figures, IR spectra (Figures S16, S17; Table S14) and visualizations of the calculated vibrational modes (Figure S18–S20), and the DFT-optimized structure coordinates (PDF)
Crystallographic information files (CIF) for 3XeF_2_·2MnF_4_, XeF_2_·MnF_4_, and XeF_2_·2MnF_4_, determined by both 3D ED and SCXRD (CSD 2368882–2368887) (ZIP-1 and ZIP-2).

## Notes

Prof. B. Žemva sadly passed away on November 2, 2023.
