# Supplementary material for: Reactive Noble-Gas Compounds Explored by 3D Electron Diffraction: XeF2–MnF4 Adducts and a Facile Sample Handling Procedure
Source: ACS Cent Sci. 2024 Aug 14;10(9):1733–41. doi: 10.1021/acscentsci.4c00815 (PMC11428288; doi:10.1021/acscentsci.4c00815)
Supplement: Supplementary file 1 — oc4c00815_si_001.pdf [file oc4c00815_si_001.pdf]

Supporting Information

Reactive noble-gas compounds explored by 3D electron diffraction: XeF_2–MnF_4 adducts and facile sample handling procedure

Klemen Motaln,[1,2] Kshitij Gurung,[3] Petr Brázda,[3] Anton Kokalj,[1,2] Kristian Radan,[1] Mirela Dragomir,[1,2] Boris Žemva,[1,2] Lukáš Palatinus,[3,*] Matic Lozinšek[1,2,*]

[1] *Jožef Stefan Institute, Jamova cesta 39, 1000 Ljubljana, Slovenia*
[2] *Jožef Stefan International Postgraduate School, Jamova cesta 39, 1000 Ljubljana, Slovenia*
[3] *Department of Structure Analysis, Institute of Physics of the Czech Academy of Sciences, Na Slovance 1999/2, Prague 8, 18221, Czech Republic*

*Corresponding authors' e-mail: palat@fzu.cz (L.P.), matic.lozinsek@ijs.si (M.L.)

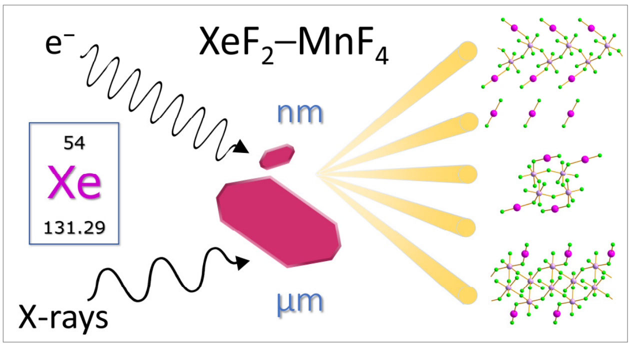

https://doi.org/10.1021/acscentsci.4c00815

Table of Contents

Figure S1. Needle-shaped crystals of $3XeF_2·2MnF_4$ as observed in the TEM S3

Table S1. 3D ED acquisition and refinement information for $3XeF_2·2MnF_4$ S3

Figure S2. Irregularly shaped crystals of $XeF_2·MnF_4$ as observed in the TEM S4

Table S2. 3D ED acquisition and refinement information for $XeF_2·MnF_4$ S4

Figure S3. Rod-shaped $XeF_2·2MnF_4$ crystal as observed in the TEM S5

Table S3. 3D ED acquisition and refinement information for $XeF_2·2MnF_4$ S5

Table S4. Unit cell parameters and refinement statistics from several independent structural determinations and their comparison with 3D ED and periodic DFT results.. .... S6

Table S5. Experimental geometrical parameters, calculated bond valences, and calculations of the significance of the difference between the SCXRD and 3D ED results for $3XeF_2·2MnF_4$........................ S7

Table S6. Experimental geometrical parameters, calculated bond valences, and calculations of the significance of the difference between the SCXRD and 3D ED results for $XeF_2·MnF_4$............................ S8

Table S7. Experimental geometrical parameters, calculated bond valences, and calculations of the significance of the difference between the SCXRD and 3D ED results for $XeF_2·2MnF_4$.......................... S9

Figure S4. The crystal packing and the unit cell of $3XeF_2·2MnF_4$ viewed along the *a*-, *b*-, and *c*-crystallographic axes.. S10

Figure S5. The crystal packing and the unit cell of $XeF_2·MnF_4$ viewed along the *a*-, *b*-, and *c*-crystallographic axes.. S11

Figure S6. The crystal packing and the unit cell of $XeF_2·2MnF_4$ viewed along the *a*-, *b*-, and *c*-crystallographic axes.. S12

Figure S7. Xe1 and Xe2 coordination environment in $3XeF_2·2MnF_4$. S13

Table S8. Non-bonded Xe···F contacts shorter than the sum of van der Waals radii for Xe and F in the crystal structure of $3XeF_2·2MnF_4$ determined by SCXRD. S14

Figure S8. Xe1 and Xe2 coordination environment in $XeF_2·MnF_4$. S15

Table S9. Non-bonded Xe···F contacts shorter than the sum of van der Waals radii for Xe and F in the crystal structure of $XeF_2·MnF_4$ determined by SCXRD. S16

Figure S9. Xe1 coordination environment in $XeF_2·2MnF_4$. S17

Table S10. Non-bonded Xe···F contacts shorter than the sum of van der Waals radii for Xe and F in the crystal structure of $XeF_2·2MnF_4$ determined by SCXRD. S17

Table S11. Observed bands in the low-temperature Raman spectra of $3XeF_2·2MnF_4$, $XeF_2·MnF_4$ and $XeF_2·2MnF_4$ S18

Figure S10. Room temperature Raman spectra of $3XeF_2·2MnF_4$, $XeF_2·MnF_4$ and $XeF_2·2MnF_4$ S19

Experimental Section – Additional Details.. S20

Figure S11. Laboratory X-ray powder diffraction patterns and Rietveld refinement results for $3XeF_2·2MnF_4$, $XeF_2·MnF_4$, and $XeF_2·2MnF_4$ S23

Figure S12. Laboratory X-ray powder diffraction pattern showing the results of Rietveld refinement and quantitative phase analysis, and Raman spectrum of the sample containing both $XeF_2·MnF_4$ and $XeF_2·2MnF_4$ S24

Figure S13. Sample loading procedure for 3D ED measurements.. S25

Figure S14. A polystyrene box setup used for the sample-loading in the glovebox............................. S25

Figure S15. Transfer of the polystyrene box containing liquid N_2 into the glove box............................ S26

Table S12. General microscope information... S27

Table S13. Summary of the crystal data and SCXRD structure refinements... S29

Figure S16. ATR-IR spectra recorded on powdered samples of $3XeF_2{\cdot}2MnF_4$, $XeF_2{\cdot}MnF_4$ and $XeF_2{\cdot}2MnF_4$. S31

Figure S17. Calculated IR spectra of $3XeF_2{\cdot}2MnF_4$, $XeF_2{\cdot}MnF_4$, and $XeF_2{\cdot}2MnF_4$ S31

Table S14. Observed bands in the experimental ATR-IR spectra of $3XeF_2{\cdot}2MnF_4$, $XeF_2{\cdot}MnF_4$ and $XeF_2{\cdot}2MnF_4$. S32

Figure S18. Calculated vibrational modes of $3XeF_2{\cdot}2MnF_4$ with frequencies above 100 cm^{-1}............. S38

Figure S19. Calculated vibrational modes of $XeF_2{\cdot}MnF_4$ with frequencies above 100 cm^{-1}.................. S45

Figure S20. Calculated vibrational modes of $XeF_2{\cdot}2MnF_4$ with frequencies above 100 cm^{-1}................ S58

References S69

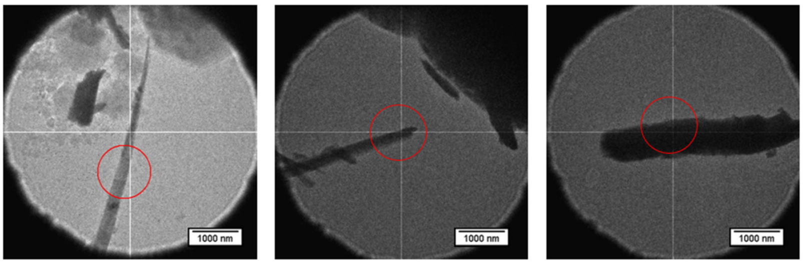

(a) (b) (c)

Figure S1. Needle-shaped crystals of $3XeF_2{\cdot}2MnF_4$ used for structural determination by 3D ED, as observed in the TEM.

Table S1. 3D ED acquisition and refinement information for $3XeF_2{\cdot}2MnF_4$.

**Collection information**			
3D ED collection method	Continuous-rotation data collection from three crystals. All atoms refined anisotropically.		
Tilt information	Crystal label	α_{min}, α_{max}, $\Delta\alpha$ (°)	
	a	−30.21, 29.92, 0.25	
	b	−39.92, 40.32, 0.25	
	c	−60.07, −12.51, 0.25	
Exposure time (ms)	283		
Beam diameter (nm)	1340		
Camera length (mm)	1500		
Electron dose information	Crystal label	Flux density (e^- $Å^{-2}$ s^{-1}) [a]	Fluence (e^- $Å^{-2}$) [b]
	a	0.0170	0.75
	b	0.0170	0.80
	c	0.0170	0.79
**Crystal information**			
Empirical formula	$Xe_3Mn_2F_{14}$		
Formula unit, Z	2		
Space group	$P2_1/n$		
a, b, c (Å)	10.2668(19), 5.0036(3), 12.4668(9)		
α, β, γ (°)	90, 96.551(11), 90		
V ($Å^3$)	636.25(13)		
Apparent mosaicities (°)	0.390, 0.205, 0.012		
Completeness (%)	73.0		
**Refinement statistics**			
	Kinematical refinement	Dynamical refinement	
d_{min} (Å)	0.71	0.71	
N_{obs}, N_{all}	1328, 1375	6129, 7903	
Parameters	38	195	
R_{obs}, wR_{obs} (%)	26.84, 33.99	13.20, 14.68	
R_{all}, wR_{all} (%)	27.15, 34.10	14.27, 14.80	

[a] Defined as particles (electrons) delivered per unit area per unit time [S1].

[b] Defined as particles delivered per unit area [S1].

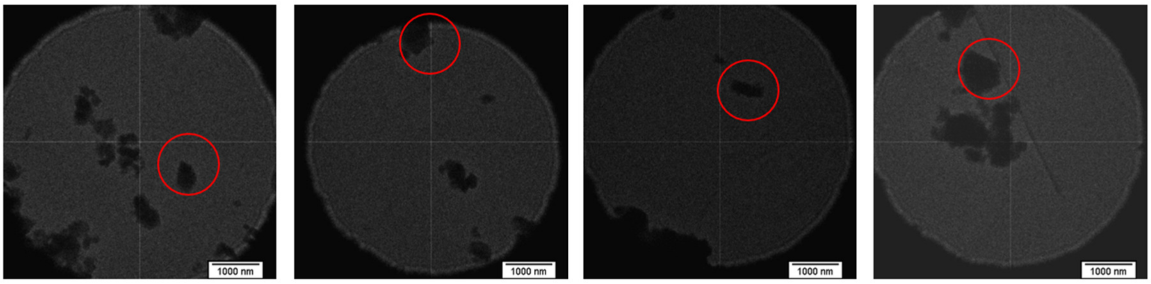

(a) (b) (c) (d)

Figure S2. Irregularly shaped crystals of $XeF_2{\cdot}MnF_4$ used for structural determination by 3D ED, as observed in the TEM.

Table S2. 3D ED acquisition and refinement information for $XeF_2{\cdot}MnF_4$.

**Collection information**			
3D ED collection method	Continuous-rotation data collection from four crystals. Xe was refined anisotropically, the other atoms were refined isotropically.		
Tilt information	Crystal label	α_{min}, α_{max}, $\Delta\alpha$ (°)	
	a	−20.10, 18.17, 0.25	
	b	−21.03, 19.85, 0.25	
	c	−19.60, 20.80, 0.25	
	d	−19.71, 20.20, 0.25	
Exposure time (ms)	354		
Beam diameter (nm)	1200		
Camera length (mm)	1500		
Electron dose information	Crystal label	Flux density (e^- Å^{-2} s^{-1})	Fluence (e^- Å^{-2})
	a	0.0170	0.75
	b	0.0170	0.80
	c	0.0170	0.79
	d	0.0200	0.78
**Crystal information**			
Empirical formula	$XeMnF_6$		
Formula unit, *Z*	8		
Space group	$P2_1/n$		
a, b, c (Å)	9.6505(9), 11.108(2), 9.7900(11)		
α, β, γ (°)	90, 96.858(8), 90		
V (Å^3)	1042.0(3)		
Apparent mosaicities (°)	0.048, 0.180, 0.128, 0.055		
Completeness (%)	87.0		
**Refinement statistics**			
	Kinematical refinement	Dynamical refinement	
d_{min} (Å)	0.71	0.71	
N_{obs}, N_{all}	943, 986	1595, 3917	
Parameters	75	166	
R_{obs}, wR_{obs} (%)	26.70, 35.37	11.80, 11.86	
R_{all}, wR_{all} (%)	27.24, 35.60	19.06, 13.26	

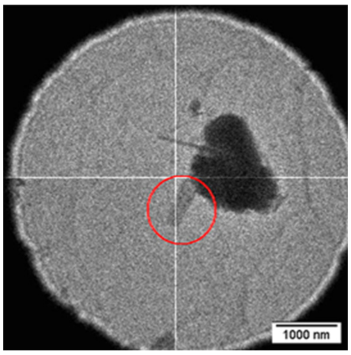

Figure S3. Rod-shaped $XeF_2 \cdot 2MnF_4$ crystal used for structural determination by 3D ED, as observed in the TEM.

Table S3. 3D ED acquisition and refinement information for $XeF_2 \cdot 2MnF_4$.

**Collection information**		
3D ED collection method	Continuous-rotation data collection from three crystals.<br>All atoms were refined anisotropically.	
Tilt information	α_{min}, α_{max}, $\Delta\alpha$ (°)	
	−45.02, 45.10, 0.30	
Exposure time (ms)	289	
Beam diameter (nm)	1120	
Camera length (mm)	1500	
Electron dose information	Flux density (e^- $Å^{-2}$ s^{-1})	Fluence (e^- $Å^{-2}$)
	0.0200	2.02
**Crystal information**		
Empirical formula	$XeMn_2F_{10}$	
Formula unit, *Z*	4	
Space group	$P2_1/n$	
a, b, c (Å)	5.2046(4), 9.9679(8), 14.6946(18)	
α, β, γ (°)	90, 96.789(6), 90	
V ($Å^3$)	756.99(13)	
Apparent mosaicity (°)	0.038	
Completeness (%)	86.0	
**Refinement statistics**		
	Kinematical refinement	Dynamical refinement
d_{min} (Å)	0.71	0.71
N_{obs}, N_{all}	1370, 1903	2285, 4358
Parameters	58	167
R_{obs}, wR_{obs} (%)	24.15, 33.08	7.70, 8.11
R_{all}, wR_{all} (%)	28.08, 34.39	10.66, 8.48

Table S4. Unit cell parameters and refinement statistics from several independent structural determinations and their comparison with 3D ED and periodic DFT results. Values in bold indicate the most precise SCXRD determination, which is discussed further in the publication. Measurements were conducted at 100 K.

Compound	$3XeF_2·2MnF_4$					$XeF_2·MnF_4$					$XeF_2·2MnF_4$			
Method	**SCXRD (Ag Kα)**	SCXRD (Mo Kα)	SCXRD (Mo Kα)	3D ED	DFT/PBE-D	**SCXRD (Mo Kα)**	SCXRD (Ag Kα)	SCXRD (Ag Kα)	3D ED	DFT/PBE-D	**SCXRD (Ag Kα)**	SCXRD (Ag Kα)	3D ED	DFT/PBE-D
a (Å)	**10.24504**	10.2318	10.2241	10.2668	10.134	**9.6430**	9.6335	9.6377	9.6505	9.635	**5.18640**	5.18758	5.2046	5.213
Difference (%)[a]		0.1	0.2	−0.2	1.1		0.1	0.1	−0.1	0.1		0.0	−0.4	−0.5
b (Å)	**4.99654**	4.99500	4.9886	5.0036	4.982	**10.9859**	11.0296	11.0256	11.108	10.976	**9.88546**	9.8868	9.9679	9.715
Difference (%)[a]		0.0	0.2	−0.1	0.3		−0.4	−0.4	−1.1	0.1		0	−0.8	1.7
c (Å)	**12.44149**	12.4326	12.4239	12.4668	12.439	**9.7927**	9.8034	9.8127	9.7900	9.570	**14.6809**	14.6776	14.6946	14.534
Difference (%)[a]		0.1	0.1	−0.2	0.0		−0.1	−0.2	0.0	2.3		0.0	−0.1	1.0
β (°)	**96.5287**	96.542	96.444	96.551	95.8	**96.979**	97.007	96.950	96.858	96.28	**96.8660**	96.891	96.789	96.47
Difference (%)[a]		0.0	0.1	0.0	0.8		0.0	0.0	0.1	0.7		0.0	0.1	0.4
V (Å^3)	**632.747**	631.27	629.66	636.25	624.76	**1029.72**	1033.87	1035.05	1042.0	1006.05	**747.292**	747.35	756.99	731.44
Difference (%)[a]		0.2	0.5	−0.6	1.3		−0.4	−0.5	−1.2	2.3		0.0	−1.3	2.1
R_1	0.0226	0.0279	0.0349	0.1320		0.0378	0.0660	0.0438	0.1180		0.0350	0.0442	0.0770	
wR_2 / wR_{all}	0.0600	0.0660	0.0780	0.1480		0.0864	0.1615	0.1139	0.1326		0.0775	0.0996	0.0848	

[a] Calculated as $100(x_{SCXRD} - x)/x_{SCXRD}$, where x_{SCXRD} is a tabulated parameter obtained from the most precise SCXRD determination and x is the tabulated parameter obtained from other SCXRD determinations, 3D ED or periodic DFT calculations.

Table S5. Experimental geometrical parameters, calculated bond valences [BV][a], and calculations of the significance of the difference[b] between the SCXRD and 3D ED results for $3XeF_2{\cdot}2MnF_4$.

Bond distance (Å)	SCXRD	3D ED	Difference significance	Bond distance (Å)	SCXRD	3D ED	Difference significance
Xe1–F1	1.9933(7) [1.07]	1.985(8) [1.10]	1.03	Mn1–F3	1.9133(6) [0.58]	1.896(9) [0.60]	1.92
Xe1–F1^{i}	1.9933(7) [1.07]	1.985(8) [1.10]	1.03	Mn1–F4	1.9058(6) [0.59]	1.893(6) [0.61]	2.12
	Σ(BV) = 2.14	Σ(BV) = 2.20		Mn1–F4^{ii}	1.9011(6) [0.60]	1.908(5) [0.59]	−1.37
Xe2–F3	2.1695(6) [1.31]	2.176(6) [0.66]	−1.08	Mn1–F5	1.7358(8) [0.93]	1.686(9) [1.07]	5.51
Xe2–F2	1.9204(7) [0.67]	1.900(8) [1.38]	2.54	Mn1–F6	1.7343(6) [0.94]	1.727(5) [0.96]	1.45
	Σ(BV) = 1.98	Σ(BV) = 2.04		Mn1–F7	1.7264(6) [0.96]	1.728(6) [0.95]	−0.27
					Σ(BV) = 4.59	Σ(BV) = 4.77	

Angle (°)	SCXRD	3D ED	Difference significance	Angle (°)	SCXRD	3D ED	Difference significance
F1–Xe1–F1^{i}	180.00(5)	180	0	F6–Mn1–F5	94.31(3)	94.4(3)	−0.30
F2–Xe2–F3	177.47(3)	177.0(3)	1.56	F6–Mn1–F4	89.87(3)	90.0(2)	−0.64
Mn1–F3–Xe2	130.55(3)	130.8(3)	−0.83	F6–Mn1–F4^{ii}	174.13(3)	174.5(3)	−1.23
F4–Mn1–F3	87.41(3)	87.0(3)	1.36	F7–Mn1–F3	89.79(3)	90.5(3)	−2.35
F4^{ii}–Mn1–F3	85.08(3)	85.6(3)	−1.72	F7–Mn1–F4	176.14(3)	176.4(3)	−0.86
F4^{ii}–Mn1–F4	87.690(11)	87.5(2)	0.95	F7–Mn1–F5	94.31(4)	93.6(3)	2.35
F5–Mn1–F4^{ii}	90.95(3)	90.4(3)	1.82	F7–Mn1–F4^{ii}	89.43(3)	89.7(2)	−1.34
F5–Mn1–F4	88.31(3)	88.7(3)	−1.29	F7–Mn1–F6	92.76(3)	92.5(3)	0.86
F5–Mn1–F3	174.27(3)	174.3(3)	−0.10	Mn1^{iii}–F4–Mn1	149.60(4)	150.4(4)	−1.99
F6–Mn1–F3	89.48(3)	89.4(3)	0.27				

Symmetry codes: (i) $-x + 1, -y + 1, -z + 1$; (ii) $-x - 1/2, y - 1/2, -z + 1/2$; (iii) $-x - 1/2, y + 1/2, -z + 1/2$.

[a] BV is given in bond valence units (vu) [S2, S3]. The following parameters have been used for the bond valence calculations: b = 0.37 Å; R_0 = 1.71 Å (Mn^{IV}–F), 2.02 Å (Xe^{II}–F) [S4].

[b] Calculated as $\Delta/\sqrt{(\sigma_{SCXRD}^2 + \sigma_{3D\ ED}^2)}$, where Δ is the difference between the SCXRD and 3D ED parameters ($x_{SCXRD} - x_{3D\ ED}$), and σ_{SCXRD} and $\sigma_{3D\ ED}$ are the standard uncertainties of the SCXRD and 3D ED parameters, respectively.

Table S6. Experimental geometrical parameters, calculated bond valences [BV][a], and calculations of the significance of the difference[b] between the SCXRD and 3D ED results for $XeF_2{\cdot}MnF_4$.

Bond distance (Å)	SCXRD	3D ED	Difference significance	Bond distance (Å)	SCXRD	3D ED	Difference significance
Xe1–F1	1.906(3) [1.36]	1.922(13) [1.30]	−1.20	Xe2–F8	1.910(3) [1.35]	1.917(18) [1.32]	−0.38
Xe1–F2	2.176(3) [0.66]	2.200(14) [0.61]	−1.68	Xe2–F9	2.180(3) [0.65]	2.189(15) [0.63]	−0.59
	Σ(BV) = 2.02	Σ(BV) = 1.91			Σ(BV) = 2.00	Σ(BV) = 1.95	
Mn1–F2	1.902(3) [0.60]	1.887(17) [0.62]	0.87	Mn2–F3	1.900(3) [0.60]	1.898(15) [0.60]	0.13
Mn1–F3	1.889(3) [0.62]	1.896(14) [0.60]	−0.49	Mn2–F4^{i}	1.903(3) [0.59]	1.929(12) [0.55]	−2.10
Mn1–F4	1.887(3) [0.62]	1.862(12) [0.66]	2.02	Mn2–F9	1.928(3) [0.55]	1.960(17) [0.51]	−1.85
Mn1–F5	1.741(3) [0.92]	1.739(15) [0.92]	0.13	Mn2–F10	1.729(3) [0.95]	1.743(12) [0.91]	−1.13
Mn1–F6	1.722(3) [0.97]	1.717(18) [0.98]	0.27	Mn2–F11	1.725(3) [0.96]	1.79(2) [0.81]	−3.21
Mn1–F7	1.721(3) [0.97]	1.65(2) [1.18]	3.51	Mn2–F12	1.720(3) [0.97]	1.732(15) [0.94]	−0.78
	Σ(BV) = 4.69	Σ(BV) = 4.97			Σ(BV) = 4.63	Σ(BV) = 4.33	

Angle (°)	SCXRD	3D ED	Difference significance	Angle (°)	SCXRD	3D ED	Difference significance
F1—Xe1—F2	178.52(14)	179.2(6)	−1.10	F3—Mn2—F9	84.25(13)	83.4(7)	1.19
F8—Xe2—F9	177.48(14)	178.2(7)	−1.01	F4i—Mn2—F9	82.96(13)	82.7(6)	0.42
F6—Mn1—F3	174.66(16)	175.3(7)	−0.89	F11—Mn2—F3	91.03(14)	92.9(8)	−2.30
F6—Mn1—F4	89.27(15)	87.3(7)	2.75	F11—Mn2—F4^{i}	92.27(14)	92.2(7)	0.10
F6—Mn1—F2	90.44(17)	90.9(8)	−0.56	F11—Mn2—F10	93.72(16)	93.0(8)	0.88
F6—Mn1—F5	92.53(16)	93.8(8)	−1.56	F11—Mn2—F9	173.54(14)	173.9(7)	−0.50
F3—Mn1—F2	86.35(13)	89.2(7)	−4.00	F10—Mn2—F3	90.43(15)	93.1(6)	−4.32
F4—Mn1—F3	86.24(13)	88.0(6)	−2.87	F10—Mn2—F4^{i}	172.62(15)	174.7(8)	−2.56
F4—Mn1—F2	85.85(13)	84.7(6)	1.87	F10—Mn2—F9	90.73(16)	92.1(7)	−1.91
F5—Mn1—F3	91.64(14)	90.9(7)	1.04	F12—Mn2—F3	172.69(14)	170.4(8)	2.82
F5—Mn1—F4	173.82(14)	168.9(9)	5.40	F12—Mn2—F4^{i}	89.76(14)	88.8(6)	1.56
F5—Mn1—F2	88.23(14)	84.2(8)	4.96	F12—Mn2—F11	94.42(15)	94.8(9)	−0.42
F7—Mn1—F6	93.92(18)	93.2(9)	0.78	F12—Mn2—F10	94.08(16)	92.1(7)	2.76
F7—Mn1—F3	89.15(14)	86.5(8)	3.26	F12—Mn2—F9	89.93(14)	88.4(8)	1.88
F7—Mn1—F4	92.28(14)	93.0(7)	−1.01	Mn1—F3—Mn2	149.24(17)	147.7(9)	1.68
F7—Mn1—F2	175.23(15)	175.2(7)	0.04	Mn1—F4—Mn2^{i}	147.93(17)	146.0(9)	2.11
F7—Mn1—F5	93.49(15)	98.0(9)	−4.94	Mn1—F2—Xe1	127.80(15)	129.7(7)	−2.65
F3—Mn2—F4^{i}	85.14(13)	85.2(6)	−0.10	Mn2—F9—Xe2	121.48(15)	120.9(7)	0.81

Symmetry code: (i) −x + 1, −y + 1, −z + 1.

[a] BV is given in bond valence units (vu) [S2, S3]. The following parameters have been used for the bond valence calculations: b = 0.37 Å; R_0 = 1.71 Å (Mn^{IV}–F), 2.02 Å (Xe^{II}–F) [S4].

[b] Calculated as $\Delta/\sqrt{(\sigma_{SCXRD}^2 + \sigma_{3D\ ED}^2)}$, where Δ is the difference between the SCXRD and 3D ED parameters ($x_{SCXRD} - x_{3D\ ED}$), and σ_{SCXRD} and $\sigma_{3D\ ED}$ are the standard uncertainties of the SCXRD and 3D ED parameters, respectively.

Table S7. Experimental geometrical parameters, calculated bond valences [BV][a], and calculations of the significance of the difference[b] between the SCXRD and 3D ED results for $XeF_2{\cdot}2MnF_4$.

Bond distance (Å)	SCXRD	3D ED	Difference significance	Bond distance (Å)	SCXRD	3D ED	Difference significance
Xe1–F1	1.8946(18) [1.40]	1.877(9) [1.47]	1.92	Mn2–F3^{i}	1.9068(16) [0.59]	1.913(4) [0.58]	−1.44
Xe1–F2	2.2829(16) [0.49]	2.286(7) [0.49]	−0.43	Mn2–F4^{ii}	1.9230(16) [0.56]	1.921(6) [0.57]	0.32
	Σ(BV) = 1.89	Σ(BV) = 1.96		Mn2–F5	1.9330(16) [0.55]	1.909(6) [0.58]	3.86
Mn1–F2	1.8181(16) [0.75]	1.823(5) [0.74]	−0.93	Mn2–F8	1.7340(16) [0.94]	1.738(5) [0.93]	−0.76
Mn1–F3	1.8907(16) [0.61]	1.891(5) [0.61]	−0.06	Mn2–F9	1.7282(16) [0.95]	1.752(6) [0.89]	−3.83
Mn1–F4	1.8789(15) [0.63]	1.885(6) [0.62]	−0.99	Mn2–F10	1.7268(16) [0.96]	1.731(6) [0.94]	−0.68
Mn1–F5	1.8499(15) [0.69]	1.872(5) [0.65]	−4.23		Σ(BV) = 4.54	Σ(BV) = 4.49	
Mn1–F6	1.7278(16) [0.95]	1.730(7) [0.95]	−0.31				
Mn1–F7	1.7253(17) [0.96]	1.713(5) [0.99]	2.33				
	Σ(BV) = 4.59	Σ(BV) = 4.56					

Angle (°)	SCXRD	3D ED	Difference significance	Angle (°)	SCXRD	3D ED	Difference significance
F1—Xe1—F2	176.84(8)	176.8(2)	0.19	F10—Mn2—F4^{ii}	176.33(8)	176.0(3)	1.06
F7—Mn1—F3	176.51(8)	177.0(3)	−1.58	F10—Mn2—F9	93.35(8)	93.4(3)	−0.16
F7—Mn1—F6	92.30(8)	91.2(3)	3.54	F10—Mn2—F5	89.64(8)	89.2(3)	1.42
F7—Mn1—F4	90.26(8)	91.1(3)	−2.71	F3i—Mn2—F4^{ii}	86.47(7)	85.9(2)	2.69
F7—Mn1—F5	90.20(8)	91.8(2)	−7.43	F3i—Mn2—F5	87.04(7)	87.2(2)	−0.76
F7—Mn1—F2	91.88(8)	91.0(2)	4.09	F8—Mn2—F3^{i}	173.99(8)	174.1(3)	−0.35
F6—Mn1—F3	90.75(8)	91.7(3)	−3.06	F8—Mn2—F4^{ii}	88.59(7)	89.0(2)	−1.93
F6—Mn1—F4	176.81(8)	176.5(2)	1.44	F8—Mn2—F5	89.33(8)	89.5(3)	−0.55
F6—Mn1—F5	91.28(8)	92.0(3)	−2.32	F4ii—Mn2—F5	87.29(7)	87.4(3)	−0.36
F6—Mn1—F2	89.58(8)	89.0(3)	1.87	F9—Mn2—F3^{i}	90.86(7)	91.0(2)	−0.66
F4—Mn1—F3	86.75(7)	86.0(2)	3.54	F9—Mn2—F8	92.52(8)	92.1(3)	1.35
F5—Mn1—F3	88.04(7)	87.7(2)	1.60	F9—Mn2—F4^{ii}	89.66(7)	89.9(3)	−0.78
F5—Mn1—F4	90.62(7)	90.6(3)	0.06	F9—Mn2—F5	176.39(7)	176.9(3)	−1.66
F2—Mn1—F3	89.83(8)	89.4(2)	2.00	Mn1—F3—Mn2^{i}	138.81(9)	138.7(4)	0.27
F2—Mn1—F4	88.43(7)	88.2(3)	0.75	Mn1—F4—Mn2^{iii}	147.19(9)	148.1(3)	−2.90
F2—Mn1—F5	177.71(8)	176.9(3)	2.61	Mn1—F5—Mn2	144.58(9)	145.7(3)	−3.58
F10—Mn2—F3^{i}	91.37(8)	91.8(2)	−2.00	Mn1—F2—Xe1	126.25(8)	126.8(3)	−1.77
F10—Mn2—F8	93.39(8)	93.1(3)	0.93				

Symmetry codes: (i) $-x, -y + 1, -z$; (ii) $x - 1, y, z$; (iii) $x + 1, y, z$.

[a] BV is given in bond valence units (vu) [S2, S3]. The following parameters have been used for the bond valence calculations: b = 0.37 Å; R_0 = 1.71 Å (Mn^{IV}–F), 2.02 Å (Xe^{II}–F) [S4].

[b] Calculated as $\Delta/\surd(\sigma_{SCXRD}^2 + \sigma_{3D\,ED}^2)$, where Δ is the difference between the SCXRD and 3D ED parameters ($x_{SCXRD} - x_{3D\,ED}$), and σ_{SCXRD} and $\sigma_{3D\,ED}$ are the standard uncertainties of the SCXRD and 3D ED parameters, respectively.

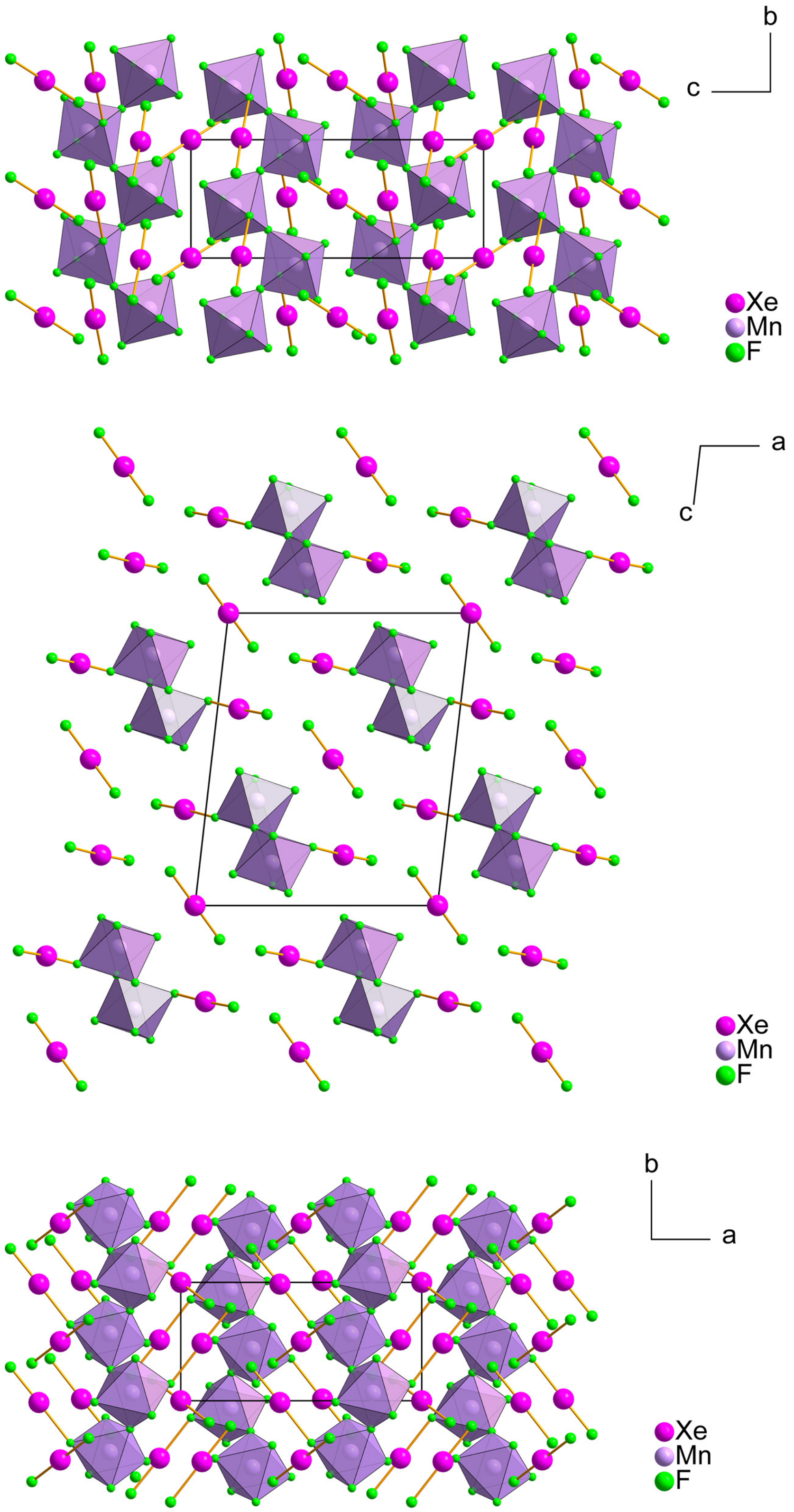

Figure S4. The crystal packing and the unit cell of $3XeF_2{\cdot}2MnF_4$ viewed along the *a*- (top), *b*- (middle), and *c*-crystallographic axis (bottom).

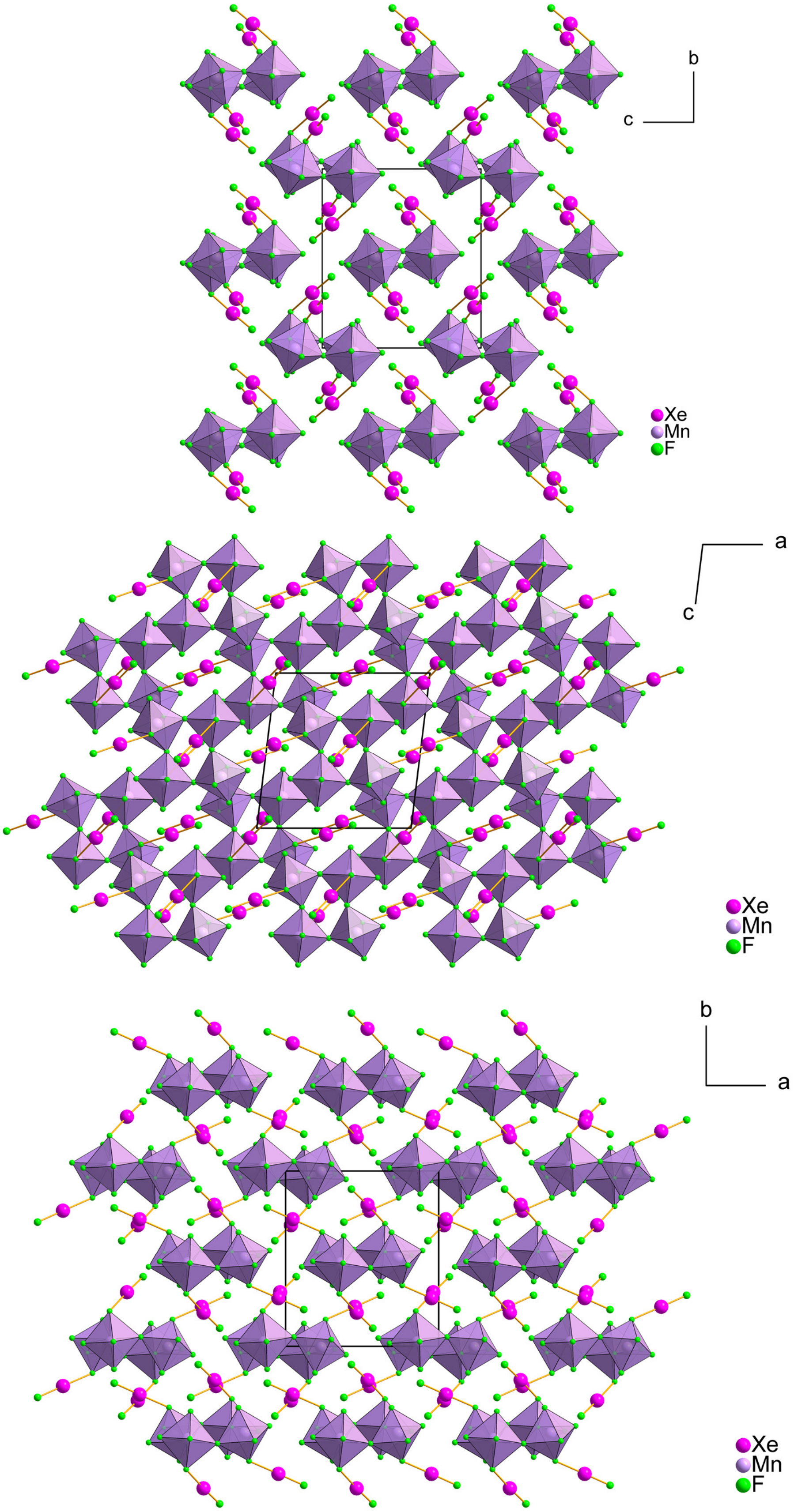

Figure S5. The crystal packing and the unit cell of $XeF_2 \cdot MnF_4$ viewed along the *a*- (top), *b*- (middle), and *c*-crystallographic axis (bottom).

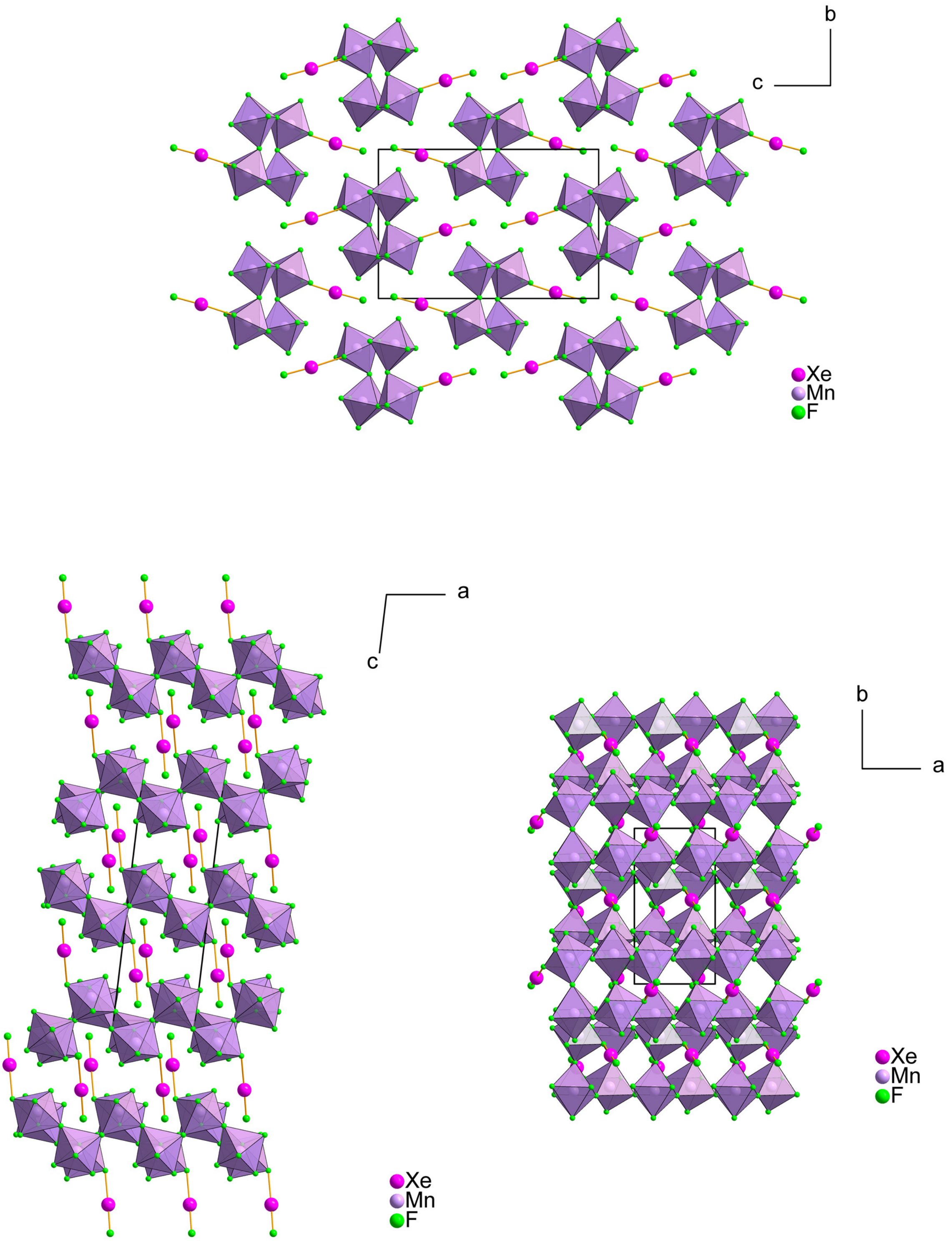

Figure S6. The crystal packing and the unit cell of $XeF_2{\cdot}2MnF_4$ viewed along the *a*- (top), *b*- (bottom left), and *c*-crystallographic axis (bottom right).

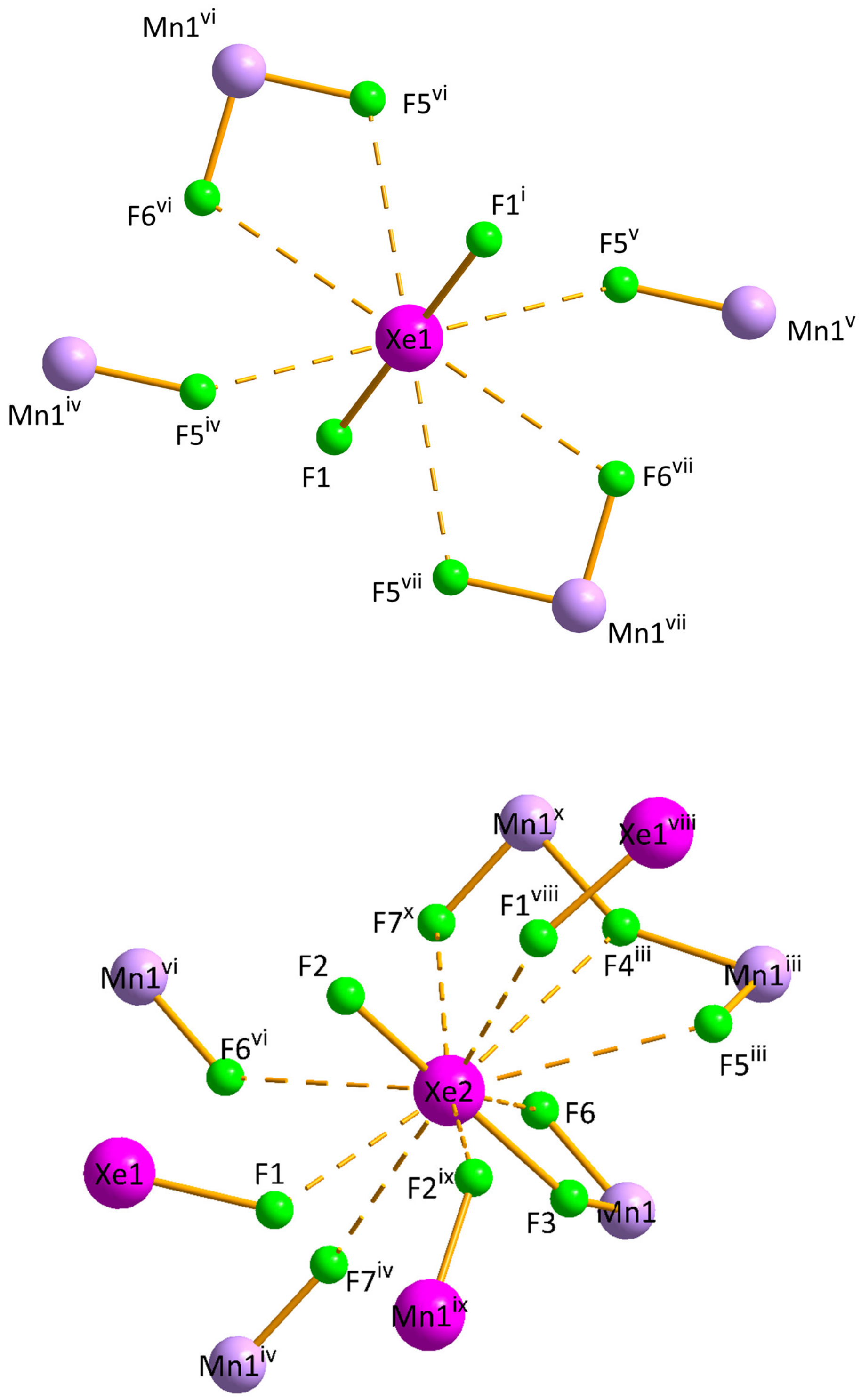

Figure S7. Xe1 (top) and Xe2 (bottom) coordination environment (including contacts up to the sum of van der Waals radii of Xe and F) in $3XeF_2{\cdot}2MnF_4$.

Table S8. Non-bonded Xe···F contacts shorter than the sum of van der Waals radii for Xe and F (3.52 Å) [S5] in the crystal structure of $3XeF_2{\cdot}2MnF_4$ determined by SCXRD.

Atom Pair	Distance (Å)
Xe1···F5^{iv}	3.2028(7)
Xe1···F5^{v}	3.2028(7)
Xe1···F5^{vi}	3.2991(7)
Xe1···F5^{vii}	3.2991(7)
Xe1···F6^{vi}	3.4726(7)
Xe1···F6^{vii}	3.4726(7)
Xe2···F1	3.1296(8)
Xe2···F6^{vi}	3.1488(6)
Xe2···F1^{viii}	3.1553(7)
Xe2···F7^{iv}	3.2112(6)
Xe2···F6	3.3100(7)
Xe2···F2^{ix}	3.3352(8)
Xe2···F4^{iii}	3.3690(6)
Xe2···F5^{iii}	3.4116(8)
Xe2···F7^{x}	3.4344(8)

Symmetry codes: (i) −*x* + 1, −*y* + 1, −*z* + 1; (ii) −*x* − 1/2, *y* – 1/2, −*z* + 1/2; (iii) −*x* − 1/2, *y* + 1/2, −*z* + 1/2; (iv) −*x*, −*y*, −*z* + 1; (v) 1 + *x*, 1 + *y*, *z*; (vi) −*x*, −*y* + 1, −*z* + 1; (vii) 1 + *x*, *y*, *z*; (viii) −*x* + 1/2, *y* + 1/2, −*z* + 1/2; (ix) −*x* + 1/2, *y* − 1/2, −*z* + 1/2; (x) *x*, 1 + *y*, *z*.

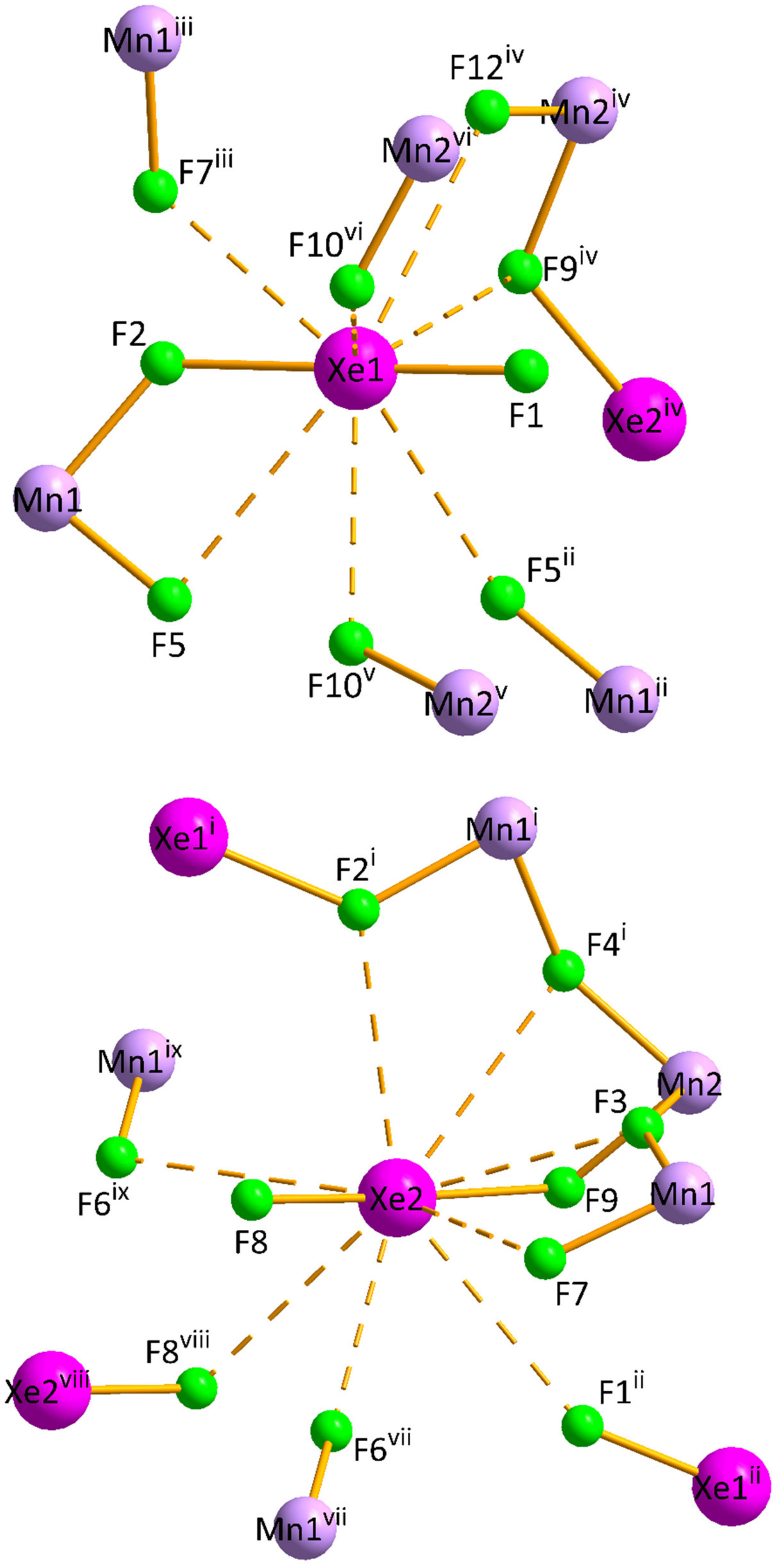

Figure S8. Xe1 (top) and Xe2 (bottom) coordination environment (including contacts up to the sum of van der Waals radii of Xe and F) in $XeF_2{\cdot}MnF_4$.

Table S9. Non-bonded Xe···F contacts shorter than the sum of van der Waals radii for Xe and F (3.52 Å) [S5] in the crystal structure of $XeF_2·MnF_4$ determined by SCXRD.

Atom Pair	Distance (Å)
Xe1···F5^{ii}	3.066(3)
Xe1···F7^{iii}	3.153(3)
Xe1···F5	3.193(3)
Xe1···F12^{iv}	3.205(3)
Xe1···F10^{v}	3.341(3)
Xe1···F11^{vi}	3.329(3)
Xe1···F9^{iv}	3.473(3)
Xe2···F6^{vii}	3.076(3)
Xe2···F3	3.131(3)
Xe2···F4^{i}	3.205(3)
Xe2···F8^{viii}	3.262(3)
Xe2···F2^{i}	3.316(3)
Xe2···F1^{ii}	3.336(4)
Xe2···F7	3.468(3)
Xe2···F6^{ix}	3.475(4)

Symmetry codes: (i) −*x* + 1, −*y* + 1, −*z* + 1; (ii) −*x*, −*y* + 1, −*z* + 1; (iii) −*x* + 1/2, *y* + 1/2, −*z* + 1/2; (iv) −*x* + 1/2, *y* + 1/2, −*z* + 3/2; (v) −*x*, −*y* + 1, −*z* + 1; (vi) *x* − 1/2, −*y* + 3/2, *z* − 1/2; (vii) −*x* + 1/2, *y* − 1/2, −*z* + 1/2; (viii) −*x* + 1, −*y*, −*z* + 1; (ix) *x* + 1/2, −*y* + 1/2, *z* + 1/2.

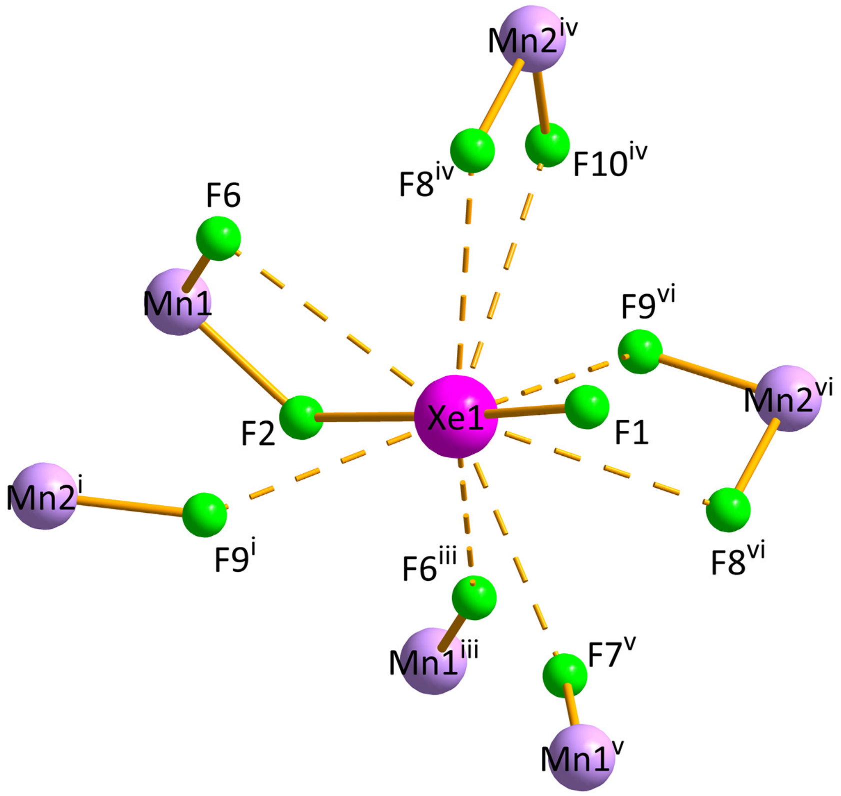

Figure S9. Xe1 coordination environment (including contacts up to the sum of van der Waals radii of Xe and F) in $XeF_2{\cdot}2MnF_4$.

Table S10. Non-bonded Xe···F contacts shorter than the sum of van der Waals radii for Xe and F (3.52 Å) [S5] in the crystal structure of $XeF_2{\cdot}2MnF_4$ determined by SCXRD.

Atom Pair	Distance (Å)
Xe1···$F9^{i}$	3.0143(16)
Xe1···$F8^{iv}$	3.0641(16)
Xe1···$F6^{iii}$	3.1046(16)
Xe1···$F7^{v}$	3.1581(16)
Xe1···F6	3.1747(17)
Xe1···$F8^{vi}$	3.2402(17)
Xe1···$F10^{iv}$	3.2950(17)
Xe1···$F9^{vi}$	3.3515(17)

Symmetry codes: (i) −*x*, −*y* + 1, −*z*; (ii) *x* − 1, *y*, *z*; (iii) *x* + 1, *y*, *z*; (iv) *x* + 1/2, −*y* + 3/2, *z* + 1/2; (v) −*x* + 3/2, −*y* − 1/2, −*z* + 1/2; (vi) *x* + 3/2, −*y* + 3/2, *z* + 1/2.

Table S11. Observed bands in the low-temperature Raman spectra of $3XeF_2{\cdot}2MnF_4$, $XeF_2{\cdot}MnF_4$ and $XeF_2{\cdot}2MnF_4$ (Figure 5) with their relative intensities denoted in parentheses.

$3XeF_2{\cdot}2MnF_4$	$XeF_2{\cdot}MnF_4$	$XeF_2{\cdot}2MnF_4$
Δν (cm^{-1})	Δν (cm^{-1})	Δν (cm^{-1})
57 (34)	60 (7)	54 (4)
68 (20)	73 (16)	60 (26)
75 (17)	96 (3)	66 (24)
101 (20)	128 (17)	72 (6)
122 (4)	141 (8)	85 (5)
159 (12)	151 (11)	95 (3)
163 (12)	162 (15)	102 (6)
176 (4)	172 (13)	132 (11)
190 (3)	207 (3)	161 (7)
204 (4)	231 (2)	175 (7)
221 (4)	244 (8)	190 (6)
228 (5)	254 (16)	215 (7)
238 (21)	268 (8)	244 (3)
252 (15)	281 (6)	254 (17)
277 (1)	313 (5)	276 (9)
298 (7)	320 (5)	289 (5)
304 (4)	332 (4)	308 (4)
313 (12)	348 (2)	313 (4)
329 (3)	374 (15)	328 (4)
381 (26)	389 (7)	355 (5)
416 (2)	404 (5)	382 (5)
445 (2)	433 (8)	407 (13)
508 (100)	581 (57)	490 (4)
574 (100)	588 (51)	530 (7)
587 (1)	665 (4)	593 (3)
668 (5)	682 (41)	612 (46)
685 (76)	700 (11)	672 (6)
696 (6)	709 (41)	683 (6)
719 (91)	721 (100)	688 (13)
		710 (27)
		728 (100)

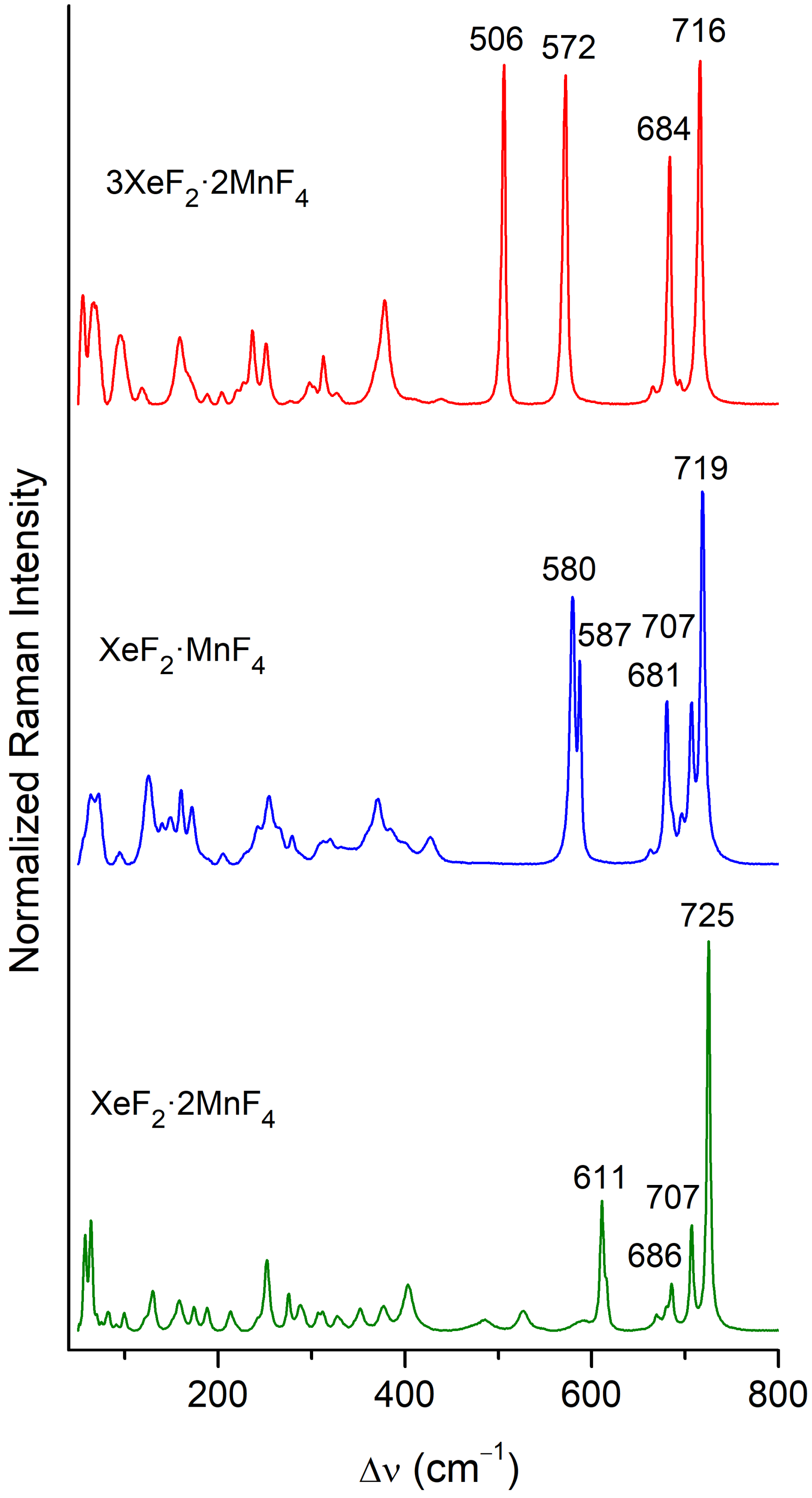

Figure S10. Room temperature Raman spectra of $3XeF_2{\cdot}2MnF_4$, $XeF_2{\cdot}MnF_4$ and $XeF_2{\cdot}2MnF_4$ recorded on powdered samples using 785 nm excitation.

Experimental Section – Additional Details

CAUTION

Anhydrous HF, F_2, XeF_2 and its compounds must be handled with great care in a well-ventilated fume hood, with protective equipment worn at all times.

Apparatus

The air and/or moisture sensitivity of the compounds studied required rigorous anhydrous conditions. Manipulation of volatile materials was performed in a nickel and PTFE (polytetrafluoroethylene) vacuum line, equipped with FEP (copolymer of tetrafluoroethylene and hexafluoropropylene) manifolds, Monel Helicoid pressure gauge, soda lime scrubber, traps cooled with liquid nitrogen, and a two-stage rotary vane pump. Solids were handled in inert atmosphere gloveboxes (MBraun and Vigor), with moisture content kept below 1 ppm at all times.

Syntheses were performed in cylindrical reaction vessels constructed from 6 mm and 16 mm internal diameter FEP tubing, heat sealed at one end and heat flared at the other and connected to the conical end of a PTFE valve, creating a pressure seal. Modified, h-shaped reaction vessels were composed of two FEP tubes, one ("main") constructed from a 16 mm internal diameter (i.d.) tube and the other ("side") arm constructed from a 6 mm i.d. tube angled at 90°. Both arms of the reactor were connected perpendicular to each other to a PTFE T-section by means of two male-to-male PTFE connectors so that the angled "side" arm ended in parallel to the wider "main" arm. The vessel was closed with a PTFE valve attached at an angle of 180° to the "main" arm. All PTFE parts were enclosed in brass housing in order to prevent stress deformation. For the synthesis of $3XeF_2{\cdot}2MnF_4$ a reactor consisting of an argon arc welded nickel vessel equipped with a PTFE-gasketed nickel valve was used. Prior to use, all vessels were passivated with 500–700 Torr of F_2 overnight.

Starting materials

Fluorine gas (Solvay Fluor, 98–99 %) was used without further purification. Commercial anhydrous HF (Linde, 99.995 %) was dried and kept dry by storage over the solid K_2NiF_6 (Advance Research Chemicals, 99.9 %) in an FEP vessel equipped with PTFE valve.

XeF_2 was synthesized by a photochemical reaction between xenon (Messer, 99.99 %) and fluorine in a thoroughly dried glass apparatus at room temperature, aided by UV light emitted from a medium-pressure Hg lamp as previously described [S6].

MnF_4 was prepared by UV irradiation (employing an air-cooled Hg lamp, 1 kW) of MnF_3 suspended in aHF in the presence of excess fluorine gas, contained in an FEP vessel (i.d. 16 mm) equipped with PTFE valve according to the procedure previously described [S7].

Syntheses

Synthesis of $3XeF_2{\cdot}2MnF_4$

In a typical experiment, nickel reaction vessel (V = 6.8 mL) was loaded with XeF_2 (545 mg, 3.219 mmol) and MnF_2 (57 mg, 0.613 mmol) in a glovebox containing an Ar atmosphere. The closed vessel was removed from the glovebox and placed in an oven heated to 120 °C for 70 hours, followed by cooling to 75 °C during the course of 1.5 hours. Excess XeF_2 was removed by pumping at room temperature. Ruby-red single crystals of $3XeF_2{\cdot}2MnF_4$ were recovered upon opening the vessel in the glovebox. Rietveld refinement of the powder X-ray diffraction (PXRD) data revealed that $3XeF_2{\cdot}2MnF_4$ is the only product formed (Figure S11).

Synthesis of $XeF_2{\cdot}MnF_4$

A cylindrical FEP reactor (8 mm o.d., 6 mm i.d.) was loaded with XeF_2 (21 mg, 0.124 mmol) and MnF_4 (14 mg, 0.107 mmol) in a glovebox containing an Ar atmosphere. The vessel was connected to a vacuum line and its contents were cooled to −196 °C by immersion in liquid N_2 and evacuated under dynamic vacuum. The vessel was then removed from the vacuum line and heat sealed to form an ampoule containing the reagents. The ampoule was transferred to the glovebox with the Ar atmosphere and placed in a wider FEP tube (19 mm o.d., 16 mm i.d.), which was sealed with a PTFE cap. This assembly was placed in an oven and heated to 130 °C for 1.5 hours, followed by cooling to 100 °C at a rate of 5 °C/hour. This procedure led to the formation of wine-red single crystals of $XeF_2{\cdot}MnF_4$, surrounded by an amorphous purple solid.

Another synthetic route for the preparation of $XeF_2{\cdot}MnF_4$ is the solvolysis of $3XeF_2{\cdot}2MnF_4$ in aHF at room temperature. In a typical experiment, $3XeF_2{\cdot}2MnF_4$ (146 mg, 0.190 mmol) was loaded into the "main" arm of an h-shaped FEP reaction vessel inside a glovebox containing an N_2 atmosphere. The vessel was connected to the vacuum line and 1.6 mL of aHF was condensed onto the sample under static vacuum. After heating the contents to room temperature and stirring with a PTFE-coated magnetic bar, a solid violet precipitate was formed, and the solution was coloured wine-red. Prolonged stirring of the solution resulted in a slow dissolution of the dark red solid phase and simultaneous precipitation of pink powder, while the colour of the solution became light pink, and after 48 hours of stirring only pink powder precipitate was present. PXRD (Figure S11) and Raman spectroscopy showed that the pink powder consisted of pure microcrystalline $XeF_2{\cdot}MnF_4$, which was not suitable for single-crystal X-ray diffraction.

Synthesis of $XeF_2{\cdot}2MnF_4$

A cylindrical FEP reactor (8 mm o.d., 6 mm i. d.) was loaded with XeF_2 (9 mg, 0.053 mmol) and MnF_4 (14 mg, 0.107 mmol) in Ar-filled glovebox. The vessel was connected to the vacuum line and evacuated under a dynamic vacuum while immersed in liquid N_2. The vessel was then removed from the vacuum line and heat sealed to form an FEP ampoule containing the reagents. The ampoule was placed in an oven heated to 135 °C and maintained at this temperature for 7 hours. The temperature was then lowered at a rate of 2 °C/hour until the temperature reached 109 °C. This resulted in the formation of

dark red crystals of $XeF_2 \cdot 2MnF_4$ surrounded by an amorphous purple phase. Rietveld refinement of the PXRD data (Figure S11) confirmed that the $XeF_2 \cdot 2MnF_4$ adduct was the only crystalline product present.

In an attempt to prepare single crystals of $XeF_2 \cdot MnF_4$ by recrystallization from aHF, it was found that a small amount of $XeF_2 \cdot 2MnF_4$ had also formed. $XeF_2 \cdot MnF_4$ was prepared from $3XeF_2 \cdot 2MnF_4$ (205 mg, 0.266 mmol) by solvolysis in 2.4 mL of aHF in an h-shaped FEP reaction vessel as described above. 2 mL of aHF was added to the formed $XeF_2 \cdot MnF_4$ and the mixture was stirred overnight. It was found that a small portion of the solid dissolved to give the light pink solution, while most of the $XeF_2 \cdot MnF_4$ remained undissolved at the bottom of the reactor. The pink solution was decanted into the side arm of the reactor, and an attempt was made to grow crystals by slowly evaporating the solvent back into the main arm by cooling it in a cryostat (its temperature decreasing from 10 to −10 °C). After all of the aHF was condensed back into the main arm, it was found that only a tiny amount of amorphous red solid formed in the side arm. Upon removal of the solvent under dynamic vacuum, the contents of the main arm were analysed by PXRD and Raman spectroscopy, revealing the presence of smaller amounts of $XeF_2 \cdot 2MnF_4$ in addition to $XeF_2 \cdot MnF_4$ (Figure S12).

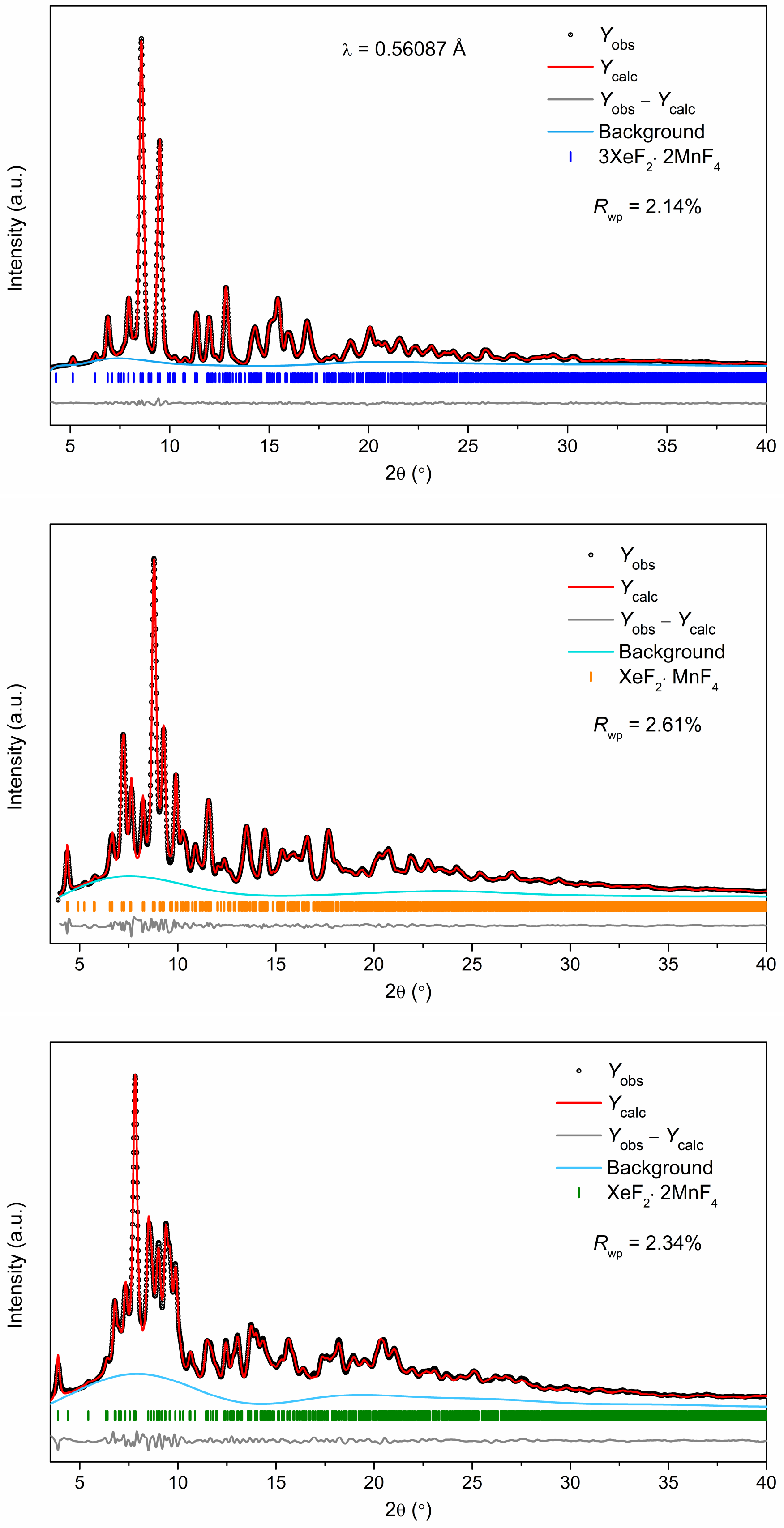

Figure S11. Laboratory powder X-ray diffraction patterns and Rietveld refinement results for samples of $3XeF_2{\cdot}2MnF_4$ (top), $XeF_2{\cdot}MnF_4$ (middle), and $XeF_2{\cdot}2MnF_4$ (bottom).

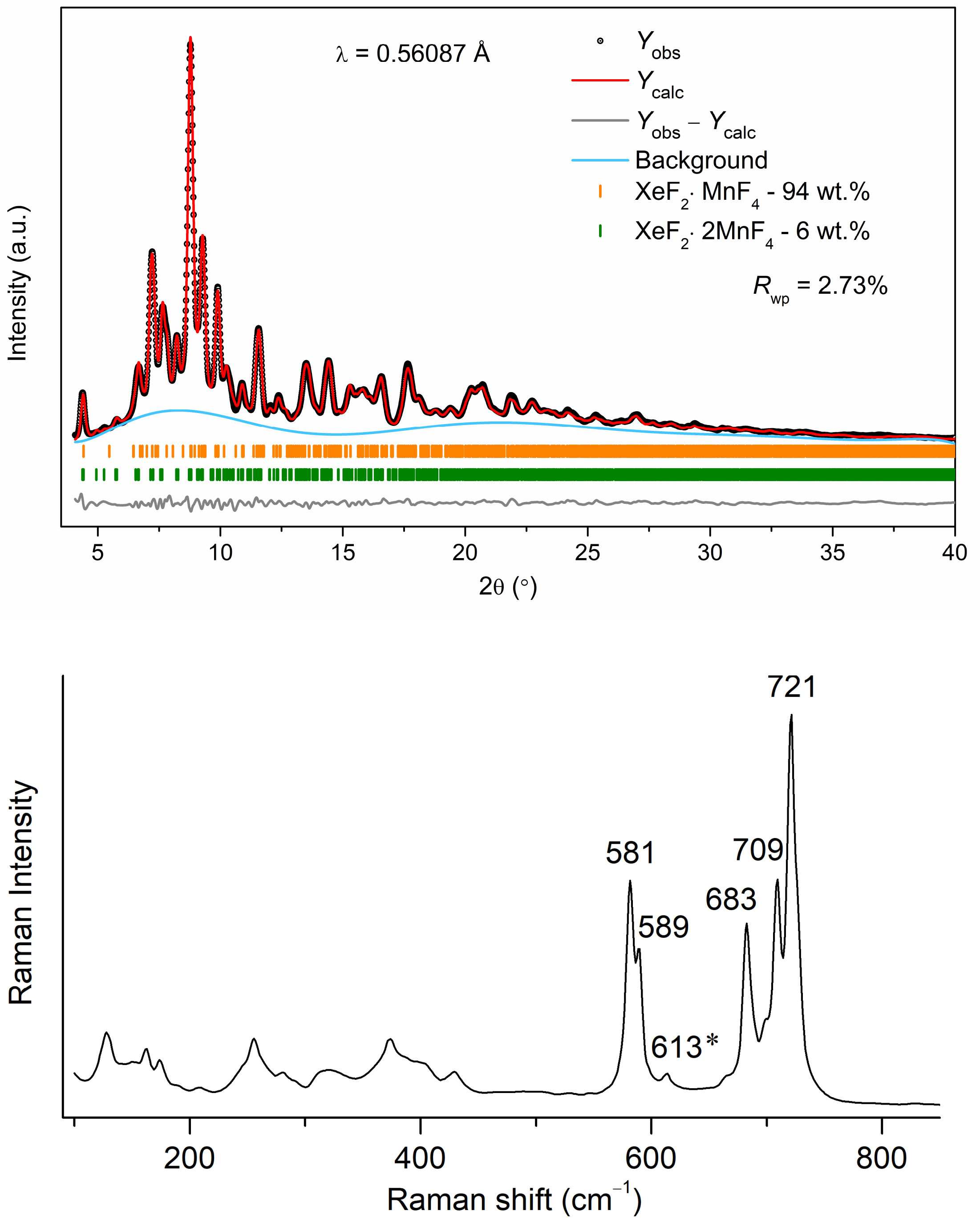

Figure S12. Laboratory powder X-ray diffraction pattern showing the results of Rietveld refinement and quantitative phase analysis (top), and Raman spectrum (bottom) of the sample containing both $XeF_2·MnF_4$ and $XeF_2·2MnF_4$. The presence of $XeF_2·2MnF_4$ on the Raman spectrum was identified by the position of the peak marked by asterisk (*), which belongs to the Xe–F stretch frequency. Raman spectrum was recorded at room temperature on a Horiba Jobin Yvon LabRAM-HR spectrometer coupled with an Olympus BXFM-ILHS microscope using 632.8 nm emission line from a He–Ne laser.

3D electron diffraction (3D ED)

Setup for the sample loading procedure for 3D ED

The main components of the sample handling and loading procedure for the study of air-sensitive compounds are: an acrylic glovebox (MBraun) filled with dry nitrogen gas (Figure S13a), the TEM cryo-transfer holder (Gatan Model 914 high tilt liquid nitrogen cryo-transfer tomography holder) inserted into a polystyrene box construct filled with liquid nitrogen (Figure S13b) and the protective sleeve, which protects the sample-loaded holder when removed from the glovebox (Figure S13c).

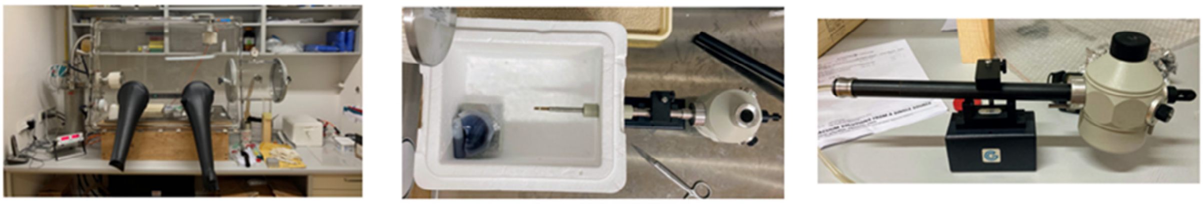

(a) (b) (c)

Figure S13. Sample loading procedure for 3D ED measurements employs: (a) an acrylic N_2-filled glovebox with a humidity meter and a circulation unit, (b) a polystyrene box construct with inserted cryo-transfer holder used for sample transfer inside the glovebox, and (c) loaded cryo-transfer holder, which is protected from the atmosphere by the protective sleeve.

During the experiment, the atmosphere in the glovebox was circulated by a blower through the molecular sieves to remove any moisture. An active moisture probe (MB-NO-SE1 MBraun) that senses moisture in the range of 0 to 500 ppm was used to monitor the environment in the glovebox. Throughout all the experiments, the moisture content in the glovebox did not rise above 1 ppm. Because of the potential risk of moisture getting into the glovebox during the procedure, it is advisable to use a separate, dedicated glovebox for this purpose.

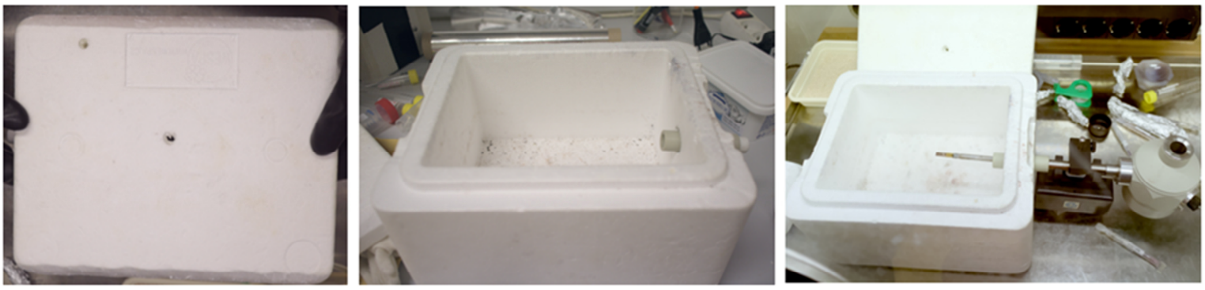

(a) (b) (c)

Figure S14. A polystyrene box setup used for the sample loading, highlighting the (a) hole that allows venting during the antechamber evacuation, (b) PVC tube insert through which the TEM holder is mounted, and (c) the entire setup with the inserted TEM holder in the glovebox.

An expanded polystyrene box with an outer dimension of 280 mm × 230 mm × 180 mm and a 3.7 L volume capacity was used for the cryo-transfer of the samples onto the sample holder (Figures S13b). Unlike the regular cryo-transfer stage, this box is compact enough to fit into the glovebox antechamber, is lighter and easier to handle, and facilitates liquid nitrogen transfer into the glovebox. A small hole was made through the lid of the box (Figure S14a) for venting during the antechamber evacuation. A second hole was made through the side of the box, high enough for the TEM holder to fit in. A PVC tube was fitted through this hole (Figure S14b), through which the TEM holder can be inserted, with the O-ring of the holder sealing the tube (Figure S14c).

To transfer the liquid nitrogen into the glovebox, the polystyrene box was filled with liquid N_2 (LN_2) just below the level of the side opening with the inserted tube. A cap was used to seal the tube to prevent the spillage of LN_2. The box was then covered with a lid and transferred into the antechamber of the glovebox. During the initial evacuation, cloudy white water vapor can be observed coming out of the lid vent (Figure S15a), which disappeared as the evacuation progressed (Figure S15b). Evacuation of the antechamber was done to 0.5 atm followed by a refill with N_2 gas to 1.0 atm. This cycle was performed 3–5 times after which the box was transferred into the glovebox. Upon removing the lid, clear LN_2 liquid was observed (Figure S15c), which is a good indication of a very low moisture level in the glovebox. The amount of liquid nitrogen lost during the evacuation–refill cycles of antechamber is negligible.

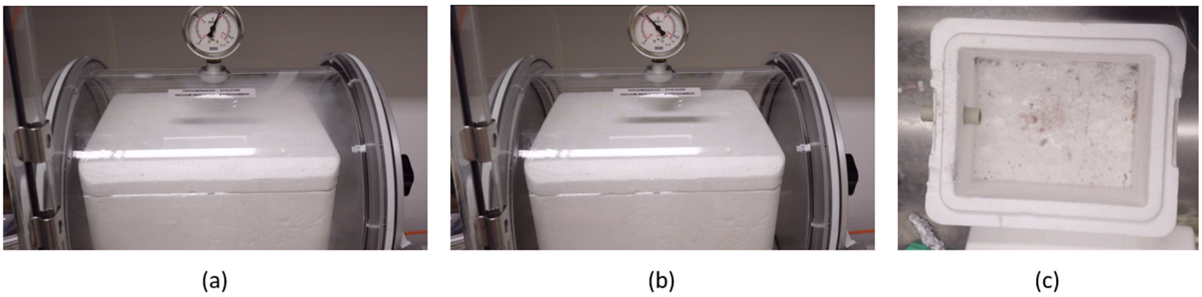

Figure S15. Transfer of the polystyrene box containing liquid N_2 (LN_2) into the glove box during (a) initial evacuation of the antechamber, (b) at the end of the evacuation of the antechamber, and (c) after the transfer of the box into the glovebox.

Once the holder was inserted through the polystyrene box (Figure S13b, S14c), a cup made of expanded polystyrene was used to dispense some of the LN_2 from the box into the Dewar of the cryo-transfer holder, and the holder was allowed to cool for 15 minutes. One benefit of this setup is that inside of the polystyrene box, right above LN_2, a layer of N_2 gas, evaporated from the liquid, is present and it protects the cooled tip of the holder from any possible moisture or other contaminants that might be present in the glovebox. The samples of XeF_2–MnF_4 adducts were very fine powders and did not require grinding before transferring onto the Cu grid (G300 from SPI supplies) on the holder. After the sample was loaded, the coverslip of the holder was closed and a sleeve was placed on the holder to protect the sample from the atmosphere before transferring the holder from the glovebox to the TEM.

Data collection and reduction

The three compounds exhibit different morphologies observed in the TEM (Figures S1–S3): $3XeF_2{\cdot}2MnF_4$ crystals form long rods, $XeF_2{\cdot}2MnF_4$ crystals form short rods, whereas $XeF_2{\cdot}MnF_4$ mostly forms agglomerates consisting of several tiny, irregularly shaped crystals that were unsuitable for structural investigations using 3D ED. However, several larger, isolated crystals of $XeF_2{\cdot}MnF_4$ could be located which proved to be suitable for 3D ED (Figure S2).

The FEI Tecnai G^2 20 transmission electron microscope with a LaB_6 cathode and equipped with a Medipix 3 hybrid pixel detector ASI Cheetah was operated at 200 kV (Table S12). Data sets were collected at a temperature of 100 K using a continuous rotation electron diffraction (cRED) method at certain tilt angle ranges with a step size of 0.25° or 0.30° as reported in Tables S1–S3. This data collection method is suitable for beam-sensitive samples such as the XeF_2–MnF_4 adducts series as it minimizes electron dose on the samples [S8]. Since the crystallinity of the XeF_2–MnF_4 sample series was poor, a major advantage of 3D ED over SCXRD is that the tiny crystals, in this case, a few hundred nanometers in size, can be easily searched for in the TEM. Indexing, determination of lattice parameters, and peak integration were performed using *PETS2* [S9]. The processed data was imported into *Jana2020* [S10] software and the crystal structures were determined using *Sir2014* [S11], which uses the Standard Direct Methods for the *ab initio* phasing of the electron diffraction data. The resulting structures were then refined kinematically and dynamically in *Jana2020*. Additional information pertaining to crystal data and refinement information are listed in Tables S1–S3.

Table S12. General microscope information

Microscope	FEI Tecnai G^2 20 transmission electron microscope
Radiation source	LaB_6
Detector (type)	Medipix 3 hybrid pixel detector ASI Cheetah (512 × 512 pixels, 24-bit dynamic range)
Accelerating voltage (kV)	200
Wavelength, λ (Å)	0.02508
Probe type	Microdiffraction
Detector pixel size (µm)	55 × 55
Calibration constant ($Å^{-1}$/pixel)	0.005664
Measurement temperature, T (K)	100

Single-crystal X-ray Diffraction

Crystal selection and mounting

Crystals of $3XeF_2{\cdot}2MnF_4$ and $XeF_2{\cdot}MnF_4$ were placed on a watch glass and covered by inert perfluorinated oil (ABCR, AB102850, perfluorodecalin, 98%, *cis* and *trans*) in a glovebox. Suitable crystals were selected under a stereomicroscope outside the glovebox and attached to the tip of a MiTeGen dual-thickness polymer loop using Baysilone paste (Bayer-Silicone, mittelviskos) as the adhesive. Immediately as the pin assembly was lifted above the surface of the oil, it was grabbed with cryo pin tongs cooled by liquid nitrogen to −196 °C and rapidly transferred to the magnetic holder on the goniometer head, where the crystal was protected by a stream of cold N_2 (100 K).

Crystals of $XeF_2{\cdot}2MnF_4$ were placed in a capsule consisting of two heat-sealed FEP tubes of different diameters (i.d. of 4.78 mm and 6 mm) in a glovebox with an inert atmosphere. The crystals were quickly transferred from the capsule onto the aluminium trough of the low-temperature crystal mounting apparatus [S12, S13], cooled to −65 °C, where they were protected by a stream of cold, dry N_2 gas. Suitable crystals were selected under a stereomicroscope and attached to the tip of a MiTeGen loop using Fomblin oil (Z25, Solvay). The loop assembly was picked up with cryo pin tongs cooled to −196 °C and quickly transferred to the goniometer head.

X-ray data collection and reduction

Single-crystal X-ray diffraction data for $3XeF_2{\cdot}2MnF_4$ and $XeF_2{\cdot}2MnF_4$ were collected with a Rigaku XtaLAB Synergy-S diffractometer equipped with a Dectris EIGER2 R CdTe 1M hybrid pixel array detector using microfocused Ag Kα radiation (λ = 0.56087 Å). Data collection was performed at a temperature of −173 °C, with the cold N_2 stream provided by an Oxford Cryosystems 800 Series Cryostream. X-ray diffraction data acquisition on single crystals of $XeF_2{\cdot}MnF_4$ was performed on a Rigaku XtaLAB Synergy-DW VHF Flow diffractometer coupled with a HyPix-Arc 150° hybrid pixel array detector using Mo Kα radiation (λ = 0.71073 Å) provided by the rotating anode X-ray source. All datasets were processed with empirical and numerical absorption correction and corrections for Lorentz and polarization effects using *CrysAlisPro* software [S14]. Crystal structures were solved with the charge flipping method using *SUPERFLIP* [S15] and refined with *SHELXL* [S16] within *Olex2* software [S17]. Figures were drawn using the program *DIAMOND* [S18]. Crystal data, data collection, and structure refinement pertaining to the crystal structures determined by SCXRD are summarized in Table S13.

Table S13. Summary of the crystal data and SCXRD structure refinements

Compound	$3XeF_2·2MnF_4$	$XeF_2·MnF_4$	$XeF_2·2MnF_4$
Formula	$Xe_3Mn_2F_{14}$	$XeMnF_6$	$XeMn_2F_{10}$
M_r	769.78	300.24	431.18
T (K)	100.00(10)	100.00(10)	100.00(14)
Crystal system	Monoclinic	Monoclinic	Monoclinic
Space group	$P2_1/n$	$P2_1/n$	$P2_1/n$
a (Å)	10.24504(11)	9.6430(5)	5.18640(8)
b (Å)	4.99654(5)	10.9859(4)	9.88546(13)
c (Å)	12.44149(14)	9.7927(3)	14.6809(2)
α (°)	90	90	90
β (°)	96.5287(10)	96.979(4)	96.8660(14)
γ (°)	90	90	90
V (Å^3)	632.747(12)	1029.72(7)	747.292(19)
Z	2	8	4
D_{calcd} (g/cm^3)	4.040	3.873	3.832
Crystal size (mm)	0.183 × 0.045 × 0.033	0.068 × 0.051 × 0.043	0.106 × 0.048 × 0.04
Radiation type	Ag Kα	Mo Kα	Ag Kα
λ (Å)	0.56087	0.71073	0.56087
Exposure time [0.5°] (s)	25	2	190
μ (mm^{-1})	5.284	9.066	4.168
F(000)	676	1064	776
θ_{max} (°)	36.760	35.205	32.489
Index ranges	$-21 \le h \le 21$	$-15 \le h \le 10$	$-9 \le h \le 9$
	$-10 \le k \le 10$	$-17 \le k \le 17$	$-17 \le k \le 18$
	$-26 \le l \le 25$	$-15 \le l \le 15$	$-27 \le l \le 27$
Reflections collected	56796	28976	73540
Independent reflections	6122	4212	5011
Reflections with $I > 2\sigma(I)$	5611	3285	4114
R_{int}	0.0835	0.0624	0.0769
R_{sigma}	0.0257	0.0437	0.0293
Data/restraints/parameters	6122/0/89	4212/0/145	5011/0/118
S	1.041	1.056	1.066
R_1, wR_2 [$I > 2\sigma(I)$]	0.0226, 0.0589	0.0378, 0.0806	0.0350, 0.0734
R_1, wR_2 [all data]	0.0251, 0.0600	0.0568, 0.0864	0.0492, 0.0775
$\Delta\rho_{min}$, $\Delta\rho_{max}$ (eÅ^{-3})	−1.956, 1.577	−1.608, 6.666	−1.399, 2.015
CSD deposition number [a]	2368885	2368886	2368887

[a] CSD 2368882–2368887 contain the supplementary crystallographic data for this paper. These data can be obtained free of charge from FIZ Karlsruhe via www.ccdc.cam.ac.uk/structures

Powder X-ray diffraction

Powder X-ray diffraction data were obtained on a Rigaku OD XtaLAB Synergy-S diffractometer with Dectris EIGER2 R CdTe 1M detector using Ag *K*α radiation (λ = 0.56087 Å). Gandolfi movement was used to enhance the random orientation of the crystallites. In a typical measurement, 6 frames were recorded with an exposure time of 300 s per frame with the detector positioned 90 mm away from the sample. The data were collected at room temperature. Two-dimensional powder diffraction frames were reduced to standard profiles using *CrysAlisPro* [S14] software and analysed using *Match!* software [S19]. The structural refinements and quantitative phase analyses were performed with the Rietveld method using the program *GSAS-II* [S20].

The samples were powdered in a mortar in an inert atmosphere glovebox and filled into quartz capillaries with a diameter of 400–600 µm, which were thoroughly dried and passivated with F_2 beforehand. The capillaries containing the samples were temporarily sealed with 25-5S halocarbon grease (Halocarbon corporation) before being removed from the glovebox and heat sealed in an H_2/O_2 flame.

Vibrational spectroscopy

Raman spectroscopy

Raman spectra were obtained on a Bruker Senterra II confocal Raman microscope using a 785 nm emission line with the power output measuring 10 mW (room temperature spectra of $XeF_2{\cdot}MnF_4$ and $XeF_2{\cdot}2MnF_4$) or 25 mW (room temperature spectrum of $3XeF_2{\cdot}2MnF_4$ and all low-temperature spectra). The spectra were measured in the 50–1410 cm^{-1} range with a resolution of 1.5 cm^{-1}. Low-temperature spectra were obtained at a temperature of −150 °C, using the Linkam LTS420 low-temperature stage. Samples for analysis were sealed in quartz capillaries as described above.

ATR-Infrared spectroscopy

Absorbance infrared spectra were measured on a Bruker Alpha II FT-IR spectrometer placed inside an N_2 atmosphere glovebox (Vigor) and equipped with a Platinum Diamond-ATR sampling module. Samples of compounds were powdered in an agate mortar prior to being placed on the diamond crystal. Spectra were recorded in the range of 400–4000 cm^{-1} with a resolution of 4 cm^{-1} and 24 scans were taken per measurement.

Infrared spectra obtained in this work (Figure S16; Table S14) are in good agreement with DFT/PBE-D calculated IR spectra (Figure S17). However, in comparison with spectra previously reported [S21], the infrared spectrum of $3XeF_2{\cdot}2MnF_4$ appears to match the spectrum, which was initially attributed to $XeF_2{\cdot}MnF_4$ [S21]. This comparison confirms that the action of molten XeF_2 (in excess) on MnF_2 always leads to the formation of $3XeF_2{\cdot}2MnF_4$. The spectrum that was initially reported for $XeF_2{\cdot}2MnF_4$ [S21] matches neither $XeF_2{\cdot}MnF_4$ nor $XeF_2{\cdot}2MnF_4$.

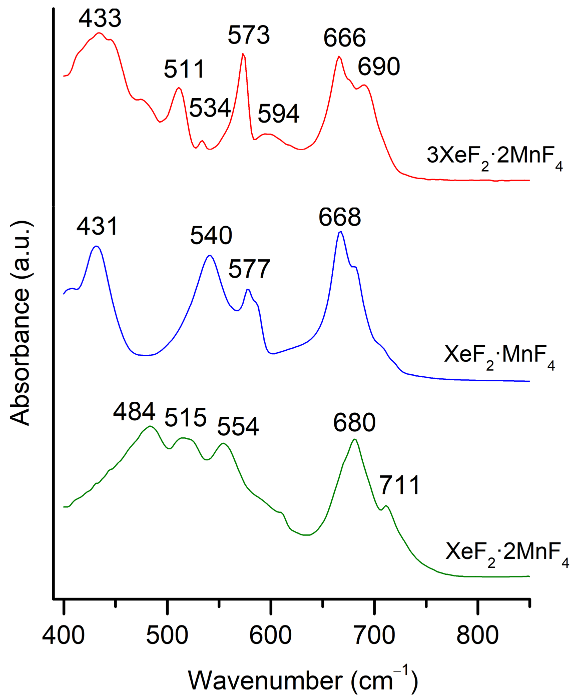

Figure S16. ATR-IR spectra recorded on powdered samples of $3XeF_2{\cdot}2MnF_4$, $XeF_2{\cdot}MnF_4$ and $XeF_2{\cdot}2MnF_4$.

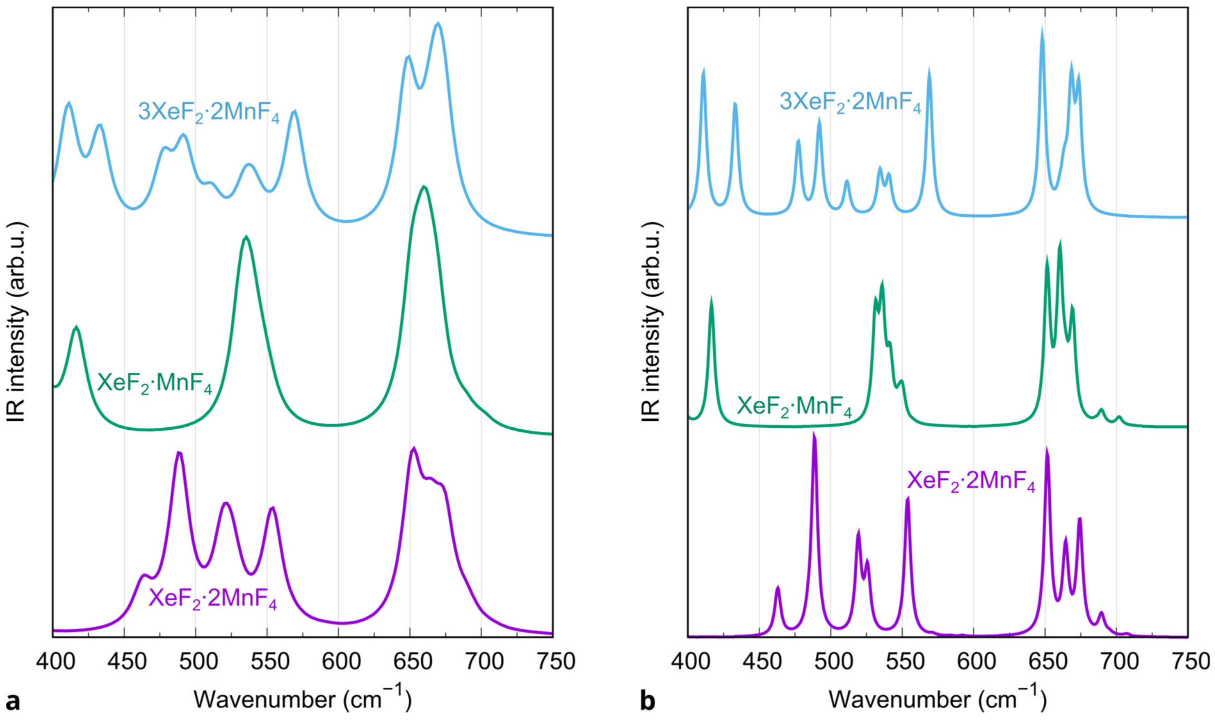

Figure S17. DFT/PBE-D calculated IR spectra of $3XeF_2{\cdot}2MnF_4$, $XeF_2{\cdot}MnF_4$, and $XeF_2{\cdot}2MnF_4$. Spectra are broadened by a Lorentzian function (a) with a fwhm of 17 cm^{-1} to mimic the experimental ATR-IR spectra and (b) with a fwhm of 5 cm^{-1}.

Table S14. Observed bands in the experimental ATR-IR spectra of $3XeF_2{\cdot}2MnF_4$, $XeF_2{\cdot}MnF_4$ and $XeF_2{\cdot}2MnF_4$.

$3XeF_2{\cdot}2MnF_4$	$XeF_2{\cdot}MnF_4$	$XeF_2{\cdot}2MnF_4$
Wavenumber	Wavenumber	Wavenumber
433	431	484
474	540	515
511	577	554
534	580 (sh)	605
573	668	680
594	680 (sh)	711
666		
690		

sh = shoulder

DFT calculations

Crystal structure DFT calculations were performed with the *Quantum ESPRESSO* (QE) open-source software package [S22, S23], using the PBE-D method that consists of the Perdew–Burke–Ernzerhof (PBE) functional [S24] and the D2 empirical dispersion correction of Grimme [S25]. Kohn–Sham orbitals were expanded with a plane-wave basis set using a wave-function kinetic-energy cutoff of 60 Ry; the cutoff for the charge density was 720 Ry. Core electrons were described implicitly using the projected-augmented-wave (PAW) potentials [S26], which were taken from pslibrary [S27] (files: F.pbe-n-kjpaw_psl.1.0.0.UPF, Mn.pbe-spn-kjpaw_psl.0.2.3.UPF, Xe.pbe-dn-kjpaw_psl.1.0.0.UPF). All degrees of freedom, including the unit cell size and shape, were relaxed using a variable-cell Broyden−Fletcher−Goldfarb−Shanno algorithm. Brillouin-zone integrations were performed using the 1 × 2 × 1, 1 × 1 × 1, and 2 × 1 × 1 k-meshes for the $3XeF_2{\cdot}2MnF_4$, $XeF_2{\cdot}MnF_4$, and $XeF_2{\cdot}2MnF_4$, respectively. The reason for this choice is that $3XeF_2{\cdot}2MnF_4$ (38 atoms in the unit cell) consists of chains extending along the b-crystallographic direction, $XeF_2{\cdot}MnF_4$ (64 atoms in the unit cell) consists of discrete rings, and $XeF_2{\cdot}2MnF_4$ (52 atoms in the unit cell) consists of double chains extending in the *a*-crystallographic direction.

The crystal structures of $3XeF_2{\cdot}2MnF_4$, $XeF_2{\cdot}MnF_4$, and $XeF_2{\cdot}2MnF_4$ were modeled with collinear spin-polarized antiferromagnetic calculations; the antiferromagnetic arrangement of the Mn ions within the unit cell of the three considered structures are depicted in Figure 4 in the main article.

Vibrational properties (normal modes, frequencies, IR spectra; Figures S17–20) were calculated at the Gamma q-point using the density-functional perturbation theory [S28] as implemented in the QE's PHonon code. Bader charges were calculated using the Bader code [S29, S30], and molecular graphics of the calculated structures were produced by the *XCrySDen* graphical package [S31].

DFT-optimized crystal structures in the *XCrySDen* structure file format

$3XeF_2{\cdot}2MnF_4$

```
CRYSTAL
PRIMVEC angstrom
  10.13412237  0.00000000   0.09779888
   0.00000000  4.98254370   0.00000000
  -1.37118080  0.00001013  12.36243685
PRIMCOORD angstrom
38
  Xe   -0.68559061     0.00000489    6.18121804
   F    0.36694259    -1.03078406    7.59425455
   F   -1.73812361     1.03079422    4.76818260
  Xe    5.06706126     2.49127209    0.04889981
   F    4.01453004     1.46048731   -1.36413785
   F    6.11959201     3.52205635    1.46193698
  Xe   -0.22373222     2.41354191   10.24840095
  Xe   -6.90009937     4.90482464    8.24635534
  Mn   -3.24921952     0.31846179   10.55635287
 Mn2   -3.87461159     2.80973867    7.93839877
   F   -1.48449841     0.68932478    9.80298168
   F   -5.63933241     3.18060691    8.69177467
   F   -3.97594897     1.24453109    9.04353480
   F   -3.14788458    -1.24674443    9.45122148
   F   -2.49270670    -0.58729762   11.84165221
   F   -4.63112478     1.90398327    6.65310327
   F    0.98408179     3.95252052   10.56067688
   F   -8.10791888     6.44380064    7.93407635
   F   -4.89113192    -0.07647455   11.02217267
   F   -2.23270088     2.41479876    7.47258461
   F   -3.18878007     1.82323347   11.44863977
   F   -3.93504973     4.31451257    7.04611795
  Xe   -1.14744792     2.56901293    2.11403677
  Xe    5.52891806     0.07772890    4.11608188
 Mn2    1.87803993     4.66409172    1.80608002
  Mn    2.50343049     2.17281485    4.42403715
   F    0.11331754     4.29322891    2.55945478
   F    4.26815079     1.80194704    3.67066218
   F    2.60476704     3.73802301    3.31890141
```

```
  F    1.77670290     6.22929778    2.91121491
  F    1.12152636     5.56985162    0.52078492
  F    3.25994435     3.07857033    5.70933344
  F   -2.35526226     1.03003315    1.80176038
  F    6.73673834    -1.46124658    4.42836091
  F    3.51995062     5.05902814    1.34026555
  F    0.86151975     2.56775537    4.88985323
  F    1.81760028     3.15932047    0.91379797
  F    2.56386985     0.66804123    5.31631934
```

$XeF_2 \cdot MnF_4$

```
CRYSTAL
PRIMVEC angstrom
   9.634672940    0.000000000    0.065604000
   0.000000000   10.975859890   -0.000000000
  -1.106278590   -0.000000000    9.505093240
PRIMCOORD angstrom
64
  Xe     3.865323420    2.116830030    5.407410180
  Xe     4.663070920    8.859029870    4.163287050
  Xe    -0.066110990    7.952169290    4.351423190
  Xe     8.594505340    3.023690610    5.219274050
  Mn     2.245081670    5.398350530    3.126288770
  Mn     6.283312680    5.577509360    6.444408470
 Mn2     2.670095480    5.249418830    6.744064600
 Mn2     5.858298870    5.726441060    2.826632640
   F     0.627109690    4.955854010    3.635477530
   F     7.901284650    6.020005880    5.935219700
   F     2.969721260    5.034505630    4.859879850
   F     5.558673090    5.941354260    4.710817380
   F     4.556416970    4.909706960    6.913271230
   F     3.971977370    6.066152930    2.657426010
   F     1.913771130    7.186423060    3.800112920
   F     6.614623220    3.789436830    5.770584310
   F    -1.813818720    8.672117850    4.916801350
   F    10.342213060    2.303742040    4.653895890
   F     1.701523060    5.856911780    1.531233780
   F     6.826871280    5.118948110    8.039463460
```

F	5.077585320	1.174066410	4.163424170
F	3.450809030	9.801793480	5.407273070
F	2.566450340	3.266964130	6.754994390
F	5.961944010	7.708895760	2.815702850
F	2.914354100	6.979416430	6.603375980
F	5.614040250	3.996443460	2.967321260
F	2.672162850	3.764187570	2.668806070
F	5.856231500	7.211672320	6.901891170
F	0.942905860	5.341384650	6.493193780
F	7.585488490	5.634475240	3.077503460
F	2.548235560	5.244532740	8.484248510
F	5.980158790	5.731327150	1.086448720
Xe	-0.707405140	7.604760400	8.883031800
Xe	-1.505152040	14.346959380	10.127154680
Xe	3.224030200	13.440098810	9.939018830
Xe	-5.436587380	8.511620970	9.071167650
Mn2	0.912836590	10.886281030	11.164152730
Mn2	-3.125393770	11.065438750	7.846033750
Mn	0.487823220	10.737348890	7.546377170
Mn	-2.700380400	11.214370900	11.463809310
F	2.530808530	10.443784030	10.654964300
F	-4.743365720	11.507935760	8.355222180
F	0.188197180	10.522435550	9.430562310
F	-2.400754360	11.429284230	9.579624170
F	-1.398498630	10.397636760	7.377170650
F	-0.814058550	11.554083030	11.633015830
F	1.244147440	12.674352940	10.490328900
F	-3.456704630	9.277366840	8.519857580
F	4.971737400	14.160047720	9.373640490
F	-7.184294580	7.791672060	9.636545990
F	1.456395460	11.344841660	12.759207810
F	-3.668952650	10.606878120	6.250978670
F	-1.919666780	6.661996350	10.127017710
F	-0.292890400	15.289723440	8.883168770
F	0.591468350	8.754893950	7.535447470
F	-2.804025530	13.196825840	11.474739010
F	0.243564360	12.467346510	7.687065910
F	-2.456121550	9.484373280	11.323120570
F	0.485755830	9.252117900	11.621635560
F	-2.698313020	12.699601880	7.388550920

```
F      2.215012840   10.829314480    7.797248140
F     -4.427570020   11.122405300   11.212938340
F      0.609683130   10.732462760    5.806193250
F     -2.822240310   11.219257020   13.203993230
```

$XeF_2 \cdot 2MnF_4$

```
CRYSTAL
PRIMVEC angstrom
   5.21312761    0.00000000    0.023010980
   0.00000000    9.71528699    0.000000000
  -1.70096150    0.00000000   14.434309420
PRIMCOORD angstrom
52
  Xe      2.34145185    5.24562787   11.60779657
   F      2.46526705    4.77453879   13.49632513
  Xe      1.17071426    4.46965911    2.84952382
   F      1.04689906    4.94074820    0.96099527
  Xe     -0.58536880    0.38798438   -4.37913637
   F     -0.70918399   -0.08310471   -6.26766493
  Xe      0.58536880   -0.38798438    4.37913637
   F      0.70918399    0.08310471    6.26766493
  Mn      0.27214214    2.97151824    8.25491304
  Mn      3.24002397    6.74376874    6.20240736
 Mn2      0.58384867    6.45851780    8.66969538
 Mn2      2.92831744    3.25676919    5.78762501
   F      1.61280795    7.09162916    7.21496380
   F      1.89935818    2.62365782    7.24235645
   F      0.17897645    1.25056642    8.55122514
   F      3.33318967    8.46472057    5.90609526
   F      1.24122253    3.24598449    9.67980694
   F      2.27094358    6.46930250    4.77751346
   F      0.46342213    4.81229340    7.71202981
   F      3.04874398    4.90299359    6.74529059
   F     -0.99833735    7.01567631    7.85252517
   F     -0.70262415    2.69961067    6.58178426
   F     -0.30224959    5.76976773   10.00766550
   F      3.81441570    3.94551925    4.44965490
   F     -1.23452010    3.32808650    9.05427876
```

F	4.74668622	6.38720048	5.40304164
F	0.70939921	8.01100929	9.46066639
F	2.80276691	1.70427769	4.99665400
F	2.18217285	5.88235937	9.43946575
F	1.32999326	3.83292762	5.01785465
Mn	1.48394092	-1.88612525	-1.02625284
Mn	-1.48394092	1.88612525	1.02625284
Mn2	1.17223438	1.60087430	-1.44103519
Mn2	-1.17223438	-1.60087430	1.44103519
F	0.14327512	2.23398567	0.01369625
F	-0.14327512	-2.23398567	-0.01369625
F	1.57710661	-3.60707707	-1.32256494
F	-1.57710661	3.60707707	1.32256494
F	0.51486053	-1.61165900	-2.45114674
F	-0.51486053	1.61165900	2.45114674
F	1.29266093	-0.04535009	-0.48336961
F	-1.29266093	0.04535009	0.48336961
F	2.75442041	2.15803282	-0.62386497
F	2.45870720	-2.15803282	0.64687594
F	2.05833265	0.91212424	-2.77900530
F	-2.05833265	-0.91212424	2.77900530
F	2.99060316	-1.52955699	-1.82561856
F	-2.99060316	1.52955699	1.82561856
F	1.04668385	3.15336580	-2.23200620
F	-1.04668385	-3.15336580	2.23200620
F	-0.42608979	1.02471588	-2.21080555
F	0.42608979	-1.02471588	2.21080555

$3XeF_2{\cdot}2MnF_4$

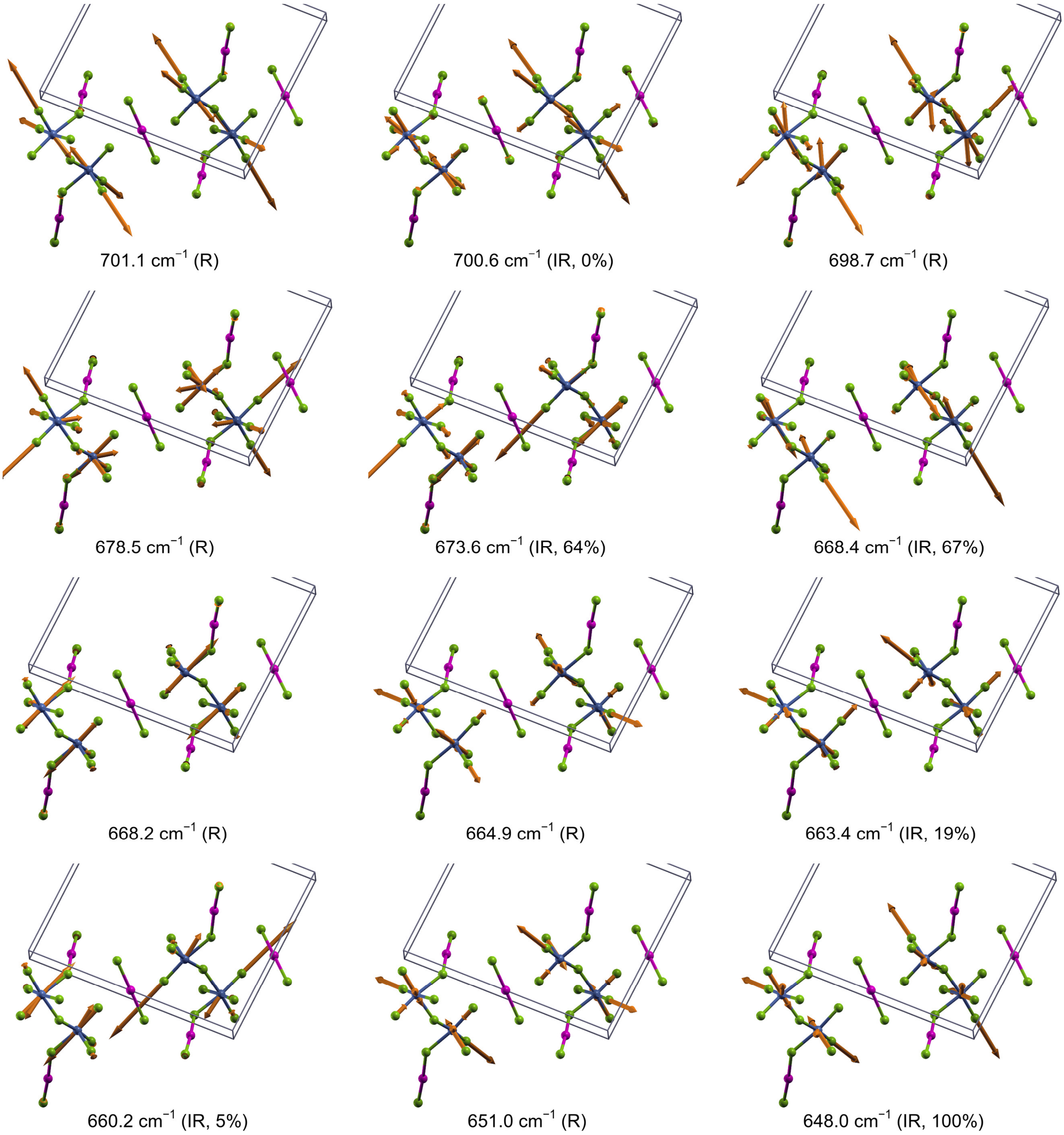

Figure S18. DFT/PBE-D calculated vibrational modes of $3XeF_2{\cdot}2MnF_4$ with frequencies above 100 cm^{-1} (sorted from high to low frequencies). The (IR) and (R) labels indicate whether a mode is IR or Raman active; for the IR active modes, normalized intensities (as percentages) are also stated. Displacement patterns are represented with arrows, whose lengths are proportional to atomic displacement magnitudes. The figure continues on the next pages.

$3XeF_2·2MnF_4$ – continued (part-2)

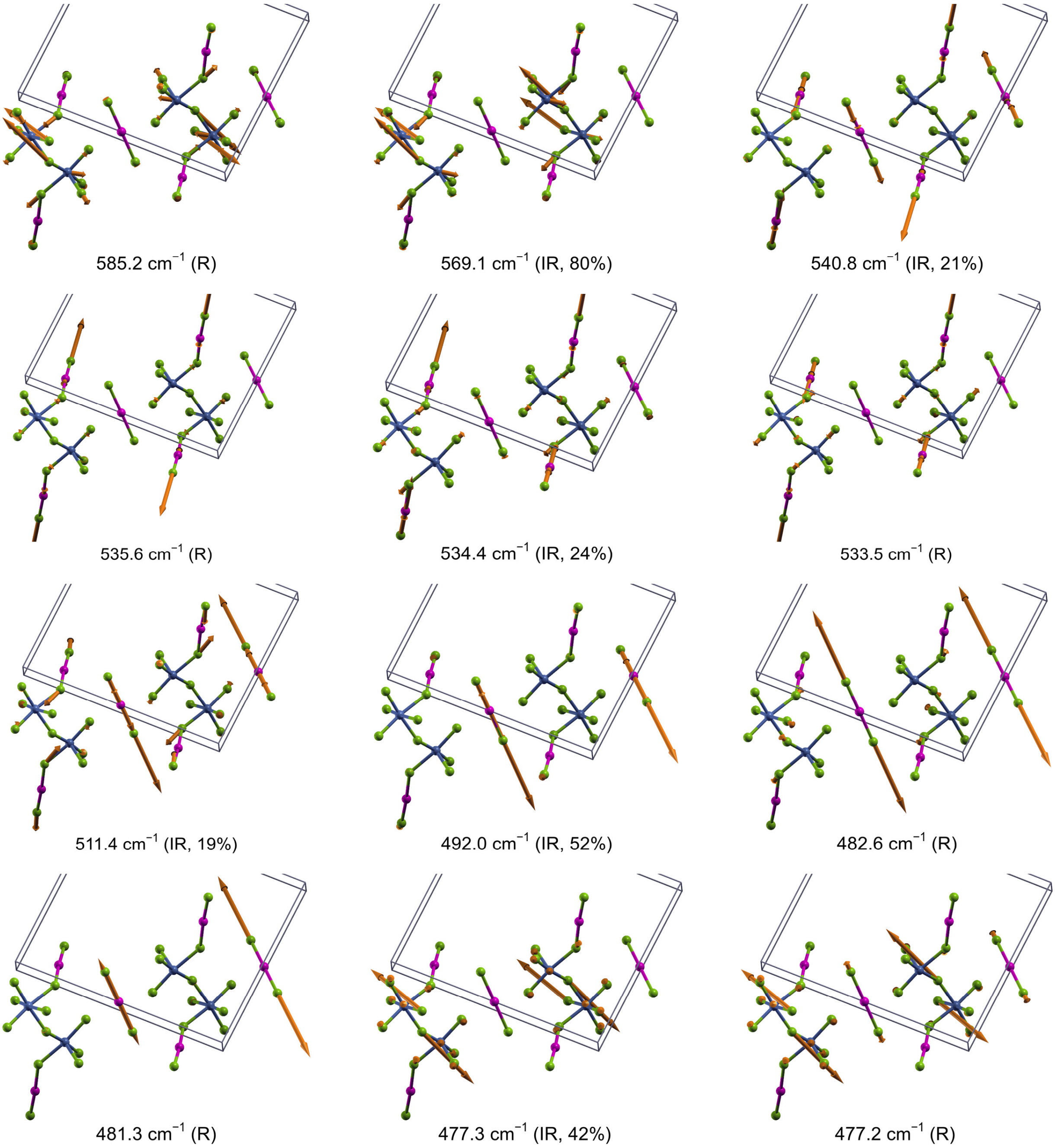

Figure S18 – continued.

$3XeF_2{\cdot}2MnF_4$ – continued (part-3)

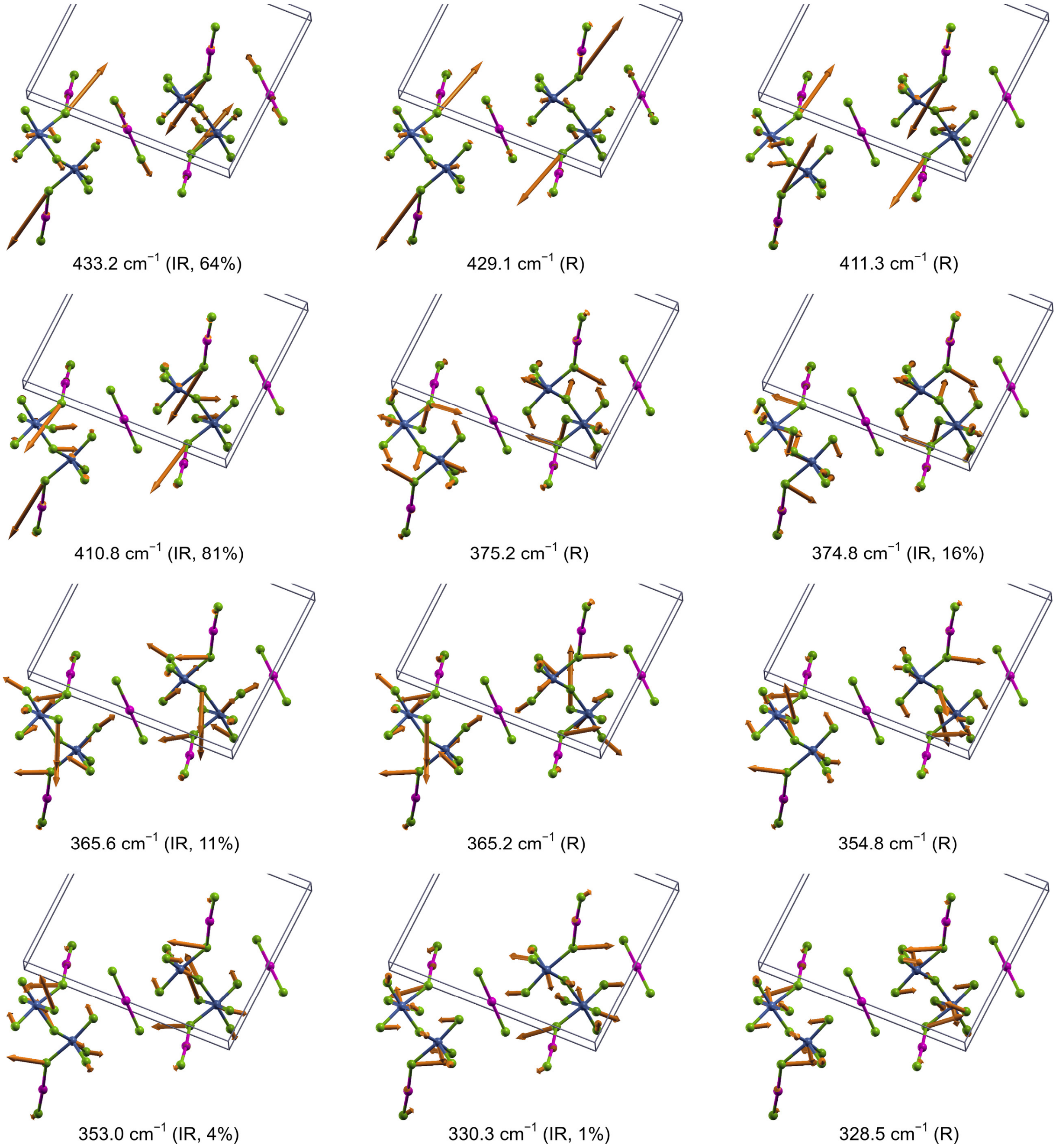

Figure S18 – continued.

$3XeF_2 \cdot 2MnF_4$ – continued (part-4)

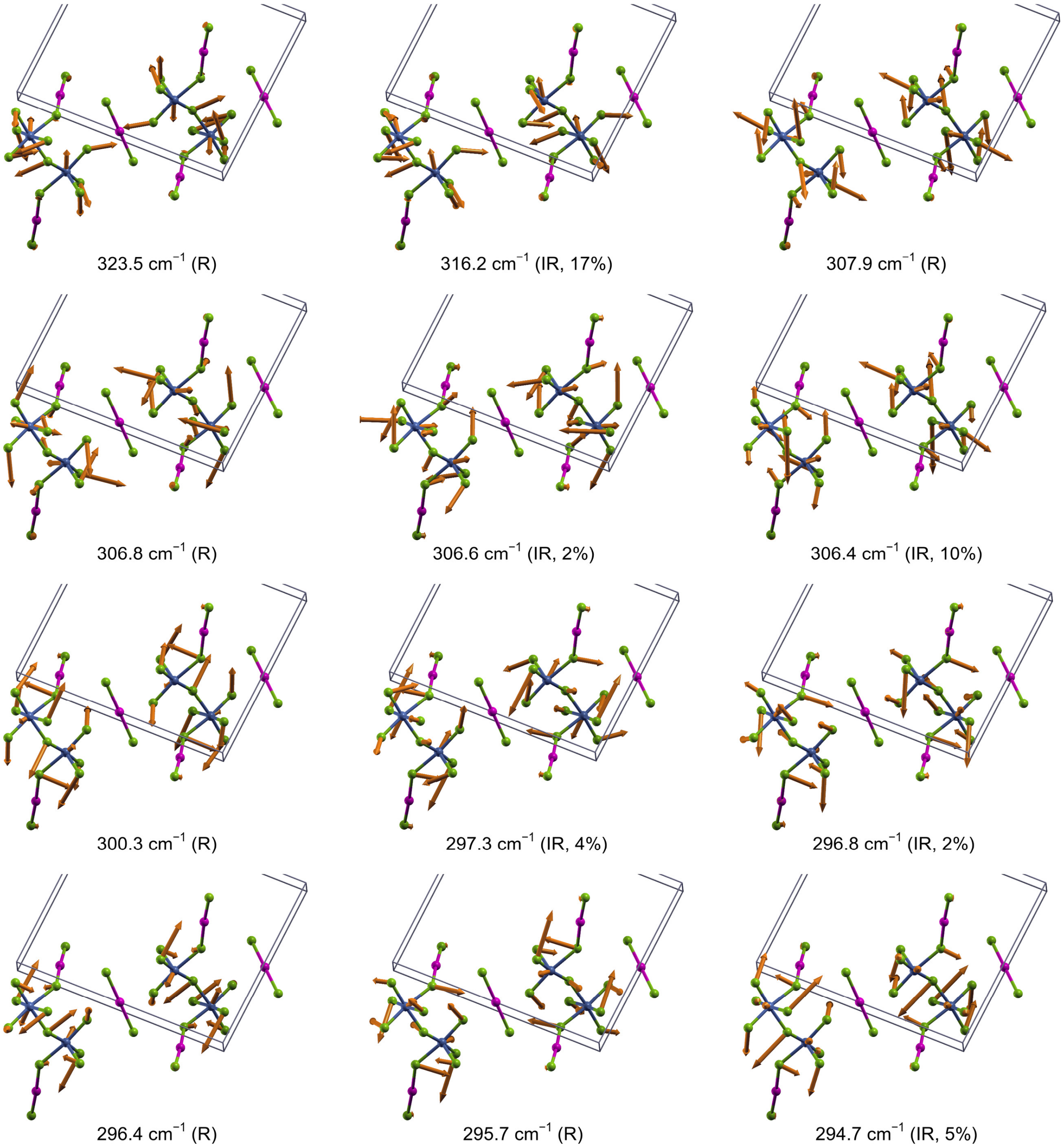

Figure S18 – continued.

$3XeF_2{\cdot}2MnF_4$ – continued (part-5)

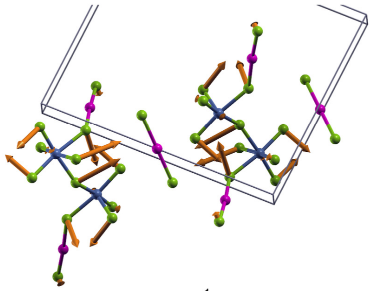

271.5 cm^{-1} (R)

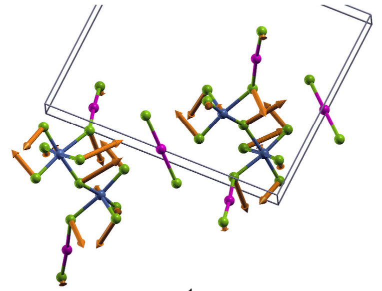

266.1 cm^{-1} (IR, 1%)

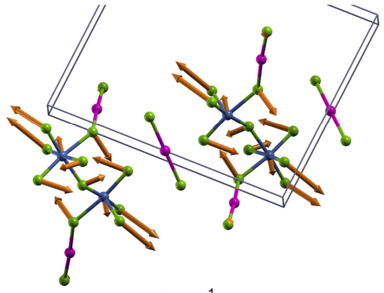

250.6 cm^{-1} (R)

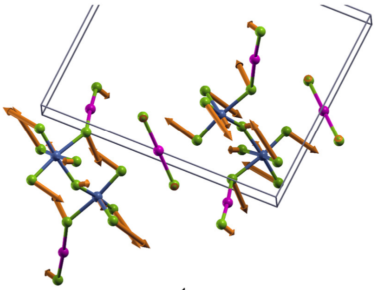

250.3 cm^{-1} (IR, 2%)

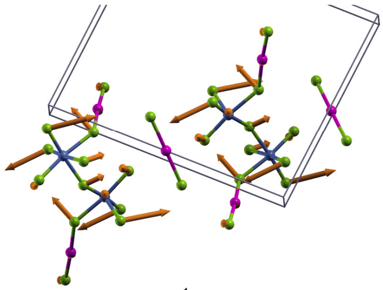

237.8 cm^{-1} (IR, 1%)

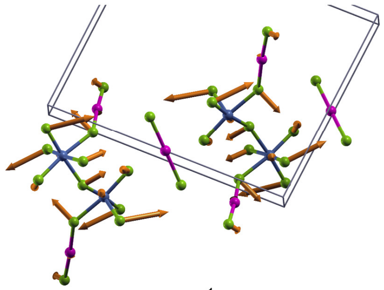

237.7 cm^{-1} (R)

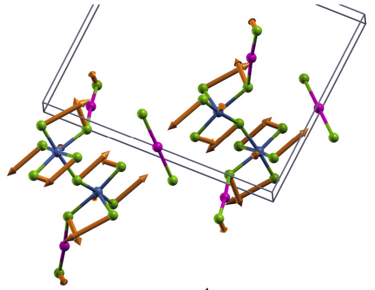

225.6 cm^{-1} (R)

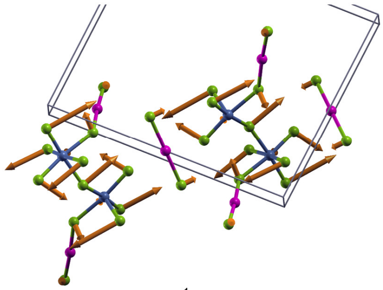

222.7 cm^{-1} (IR, 0%)

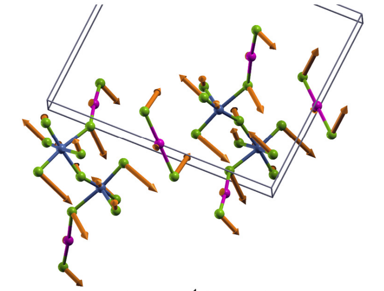

220.4 cm^{-1} (IR, 3%)

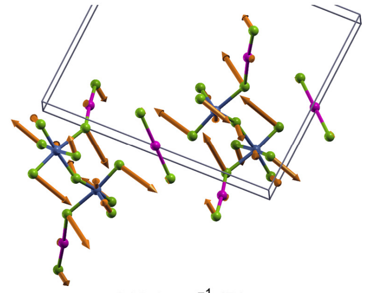

216.3 cm^{-1} (R)

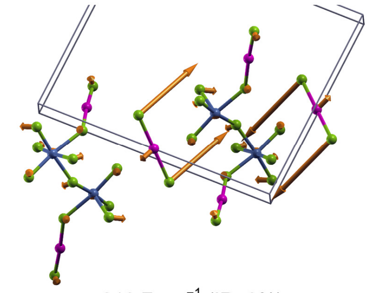

213.7 cm^{-1} (IR, 0%)

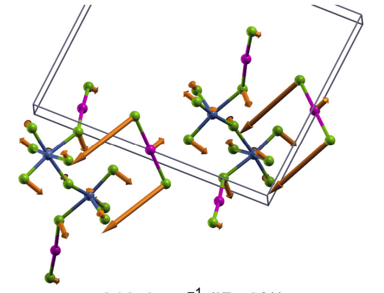

212.4 cm^{-1} (IR, 9%)

Figure S18 – continued.

$3XeF_2 \cdot 2MnF_4$ – continued (part-6)

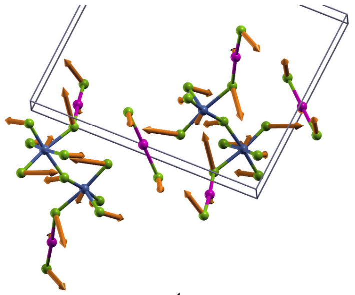

201.6 cm^{-1} (IR, 1%)

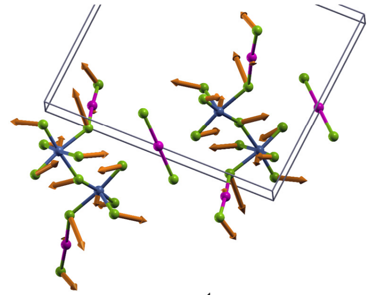

199.9 cm^{-1} (R)

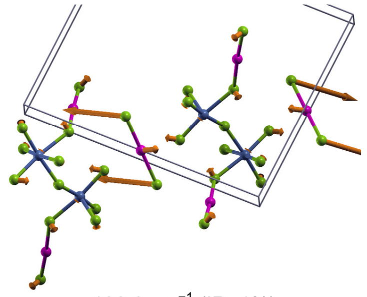

198.0 cm^{-1} (IR, 1%)

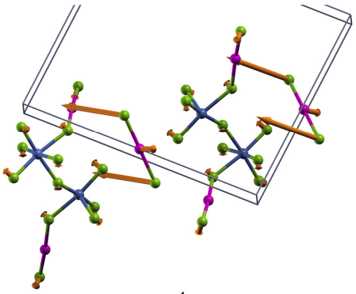

196.4 cm^{-1} (IR, 4%)

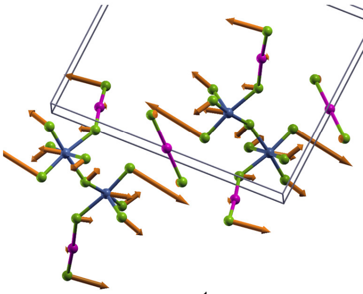

191.0 cm^{-1} (R)

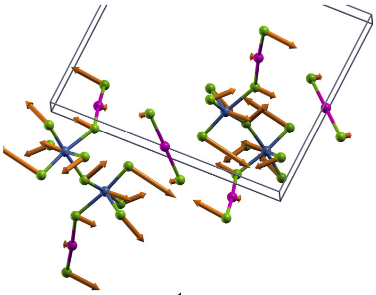

184.8 cm^{-1} (IR, 0%)

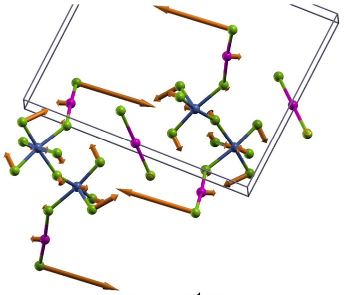

176.9 cm^{-1} (R)

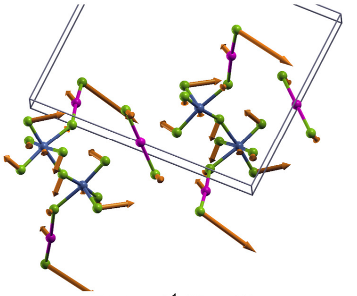

170.6 cm^{-1} (IR, 6%)

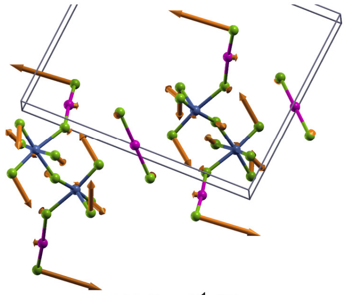

169.7 cm^{-1} (R)

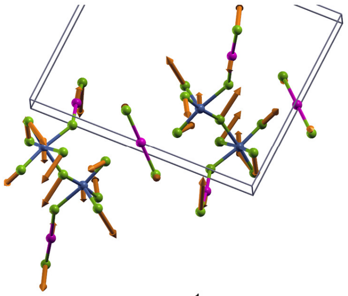

167.6 cm^{-1} (R)

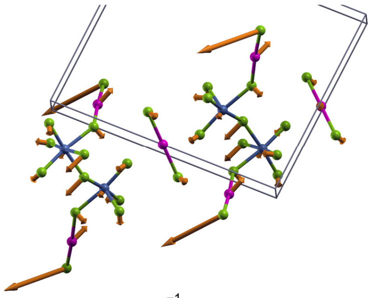

164.2 cm^{-1} (IR, 5%)

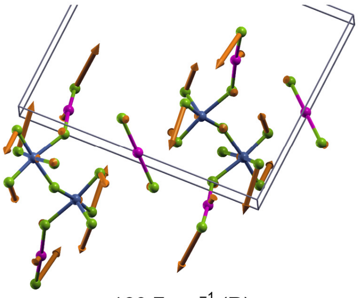

160.7 cm^{-1} (R)

Figure S18 – continued.

$3XeF_2·2MnF_4$ – continued (part-7)

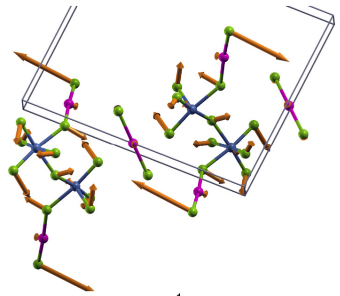
157.5 cm^{-1} (IR, 0%)

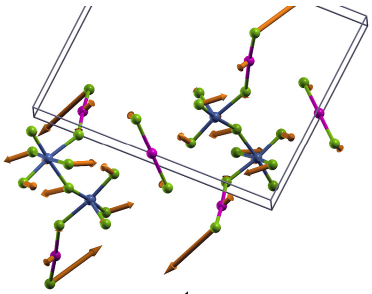
152.0 cm^{-1} (IR, 2%)

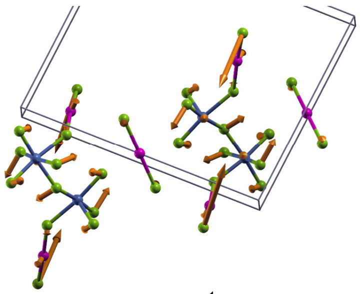
151.9 cm^{-1} (R)

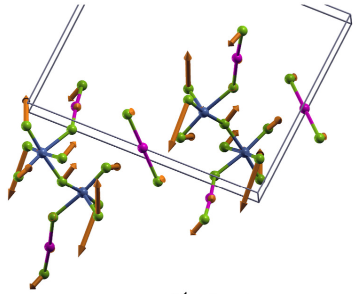
149.2 cm^{-1} (IR, 0%)

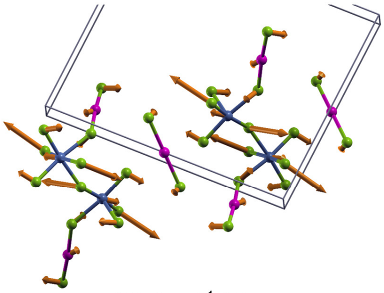
139.1 cm^{-1} (R)

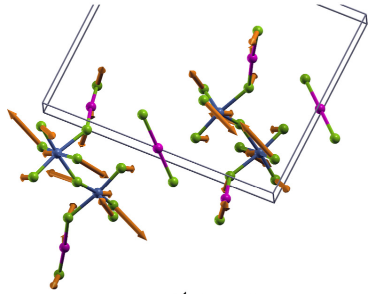
137.7 cm^{-1} (IR, 0%)

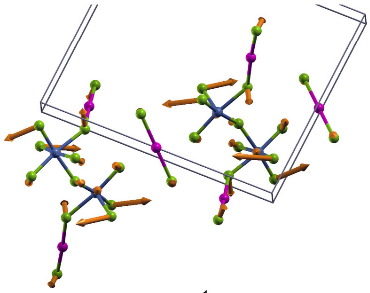
134.5 cm^{-1} (R)

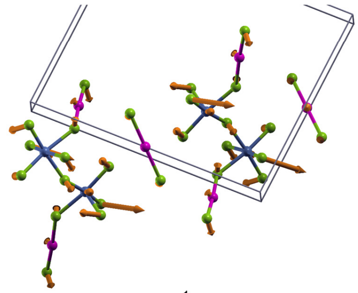
132.3 cm^{-1} (IR, 1%)

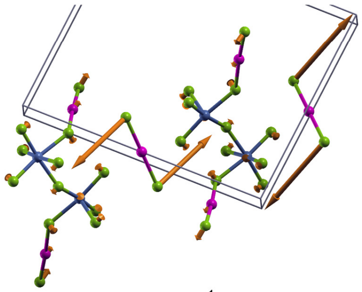
115.7 cm^{-1} (R)

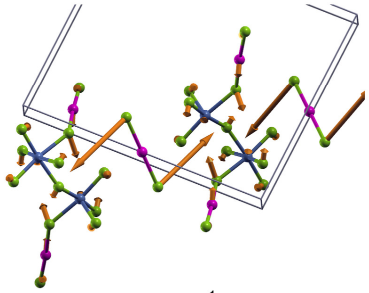
113.7 cm^{-1} (R)

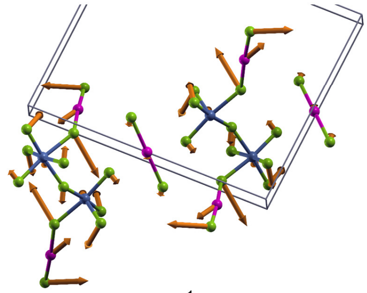
112.7 cm^{-1} (IR, 1%)

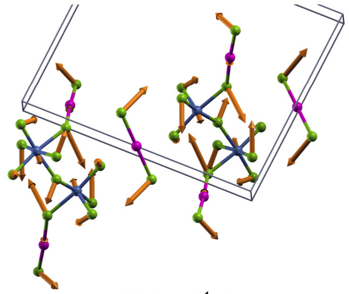
109.0 cm^{-1} (R)

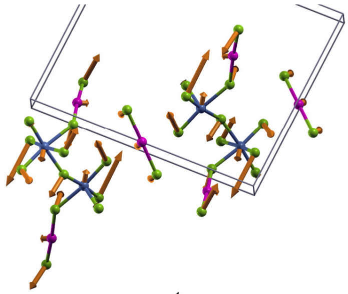
102.7 cm^{-1} (IR, 1%)

Figure S18 – continued.

$XeF_2 \cdot MnF_4$

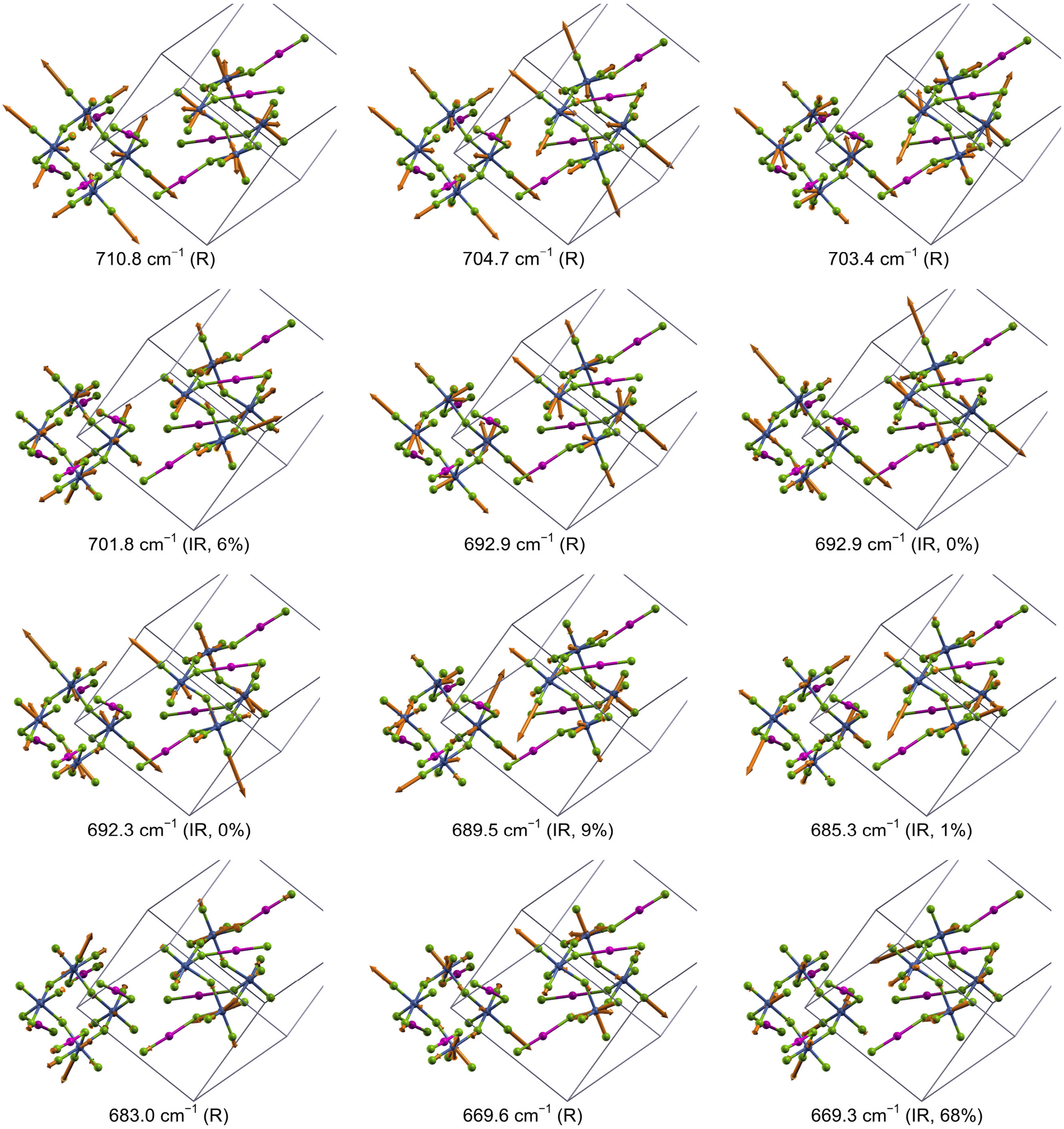

Figure S19. DFT/PBE-D calculated vibrational modes of $XeF_2 \cdot MnF_4$ with frequencies above 100 cm^{-1} (sorted from high to low frequencies). The (IR) and (R) labels indicate whether a mode is IR or Raman active; for the IR active modes, normalized intensities (as percentages) are also stated. The figure continues on the next pages.

$XeF_2 \cdot MnF_4$ – continued (part-2)

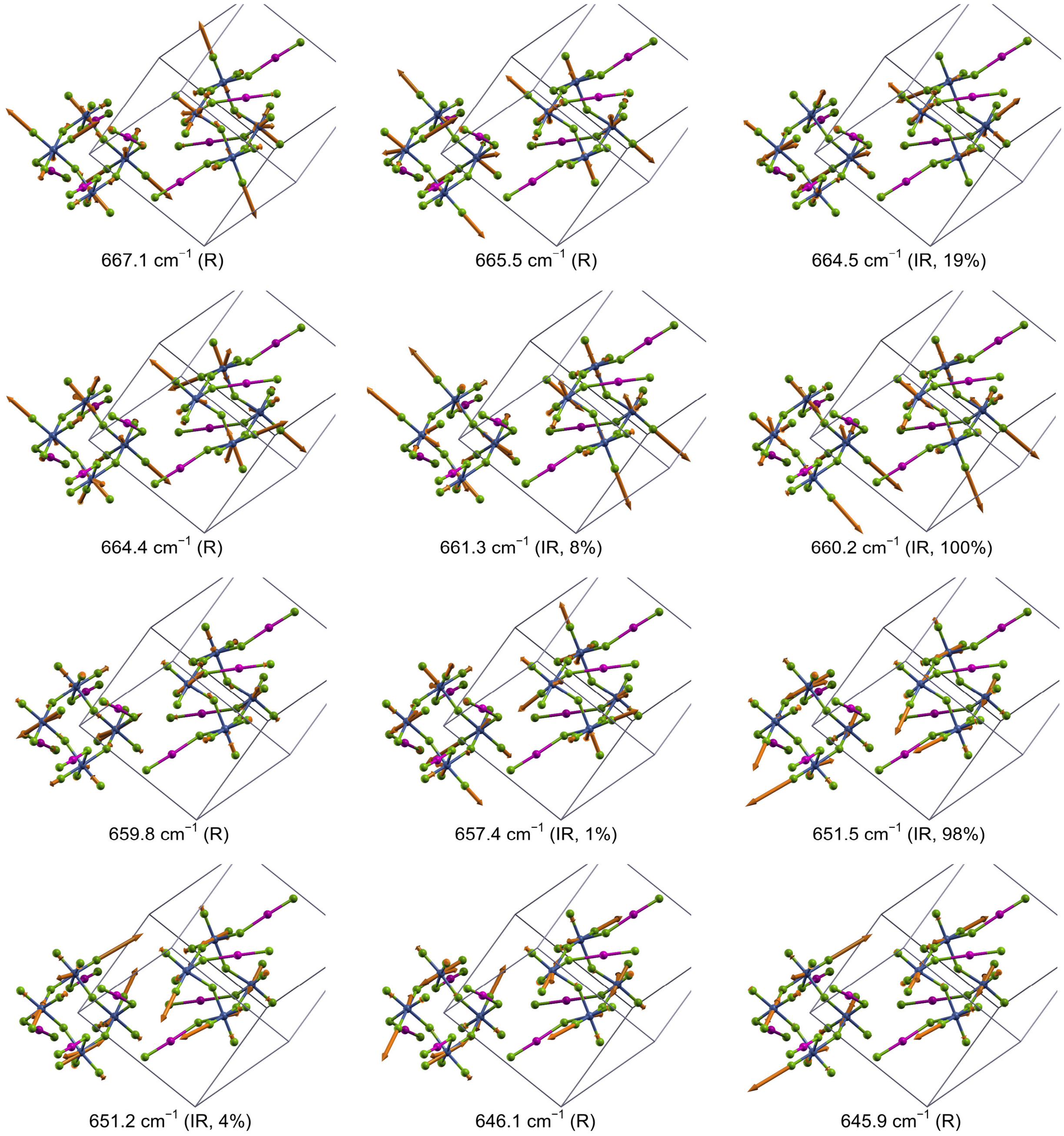

Figure S19 – continued.

$XeF_2 \cdot MnF_4$ – continued (part-3)

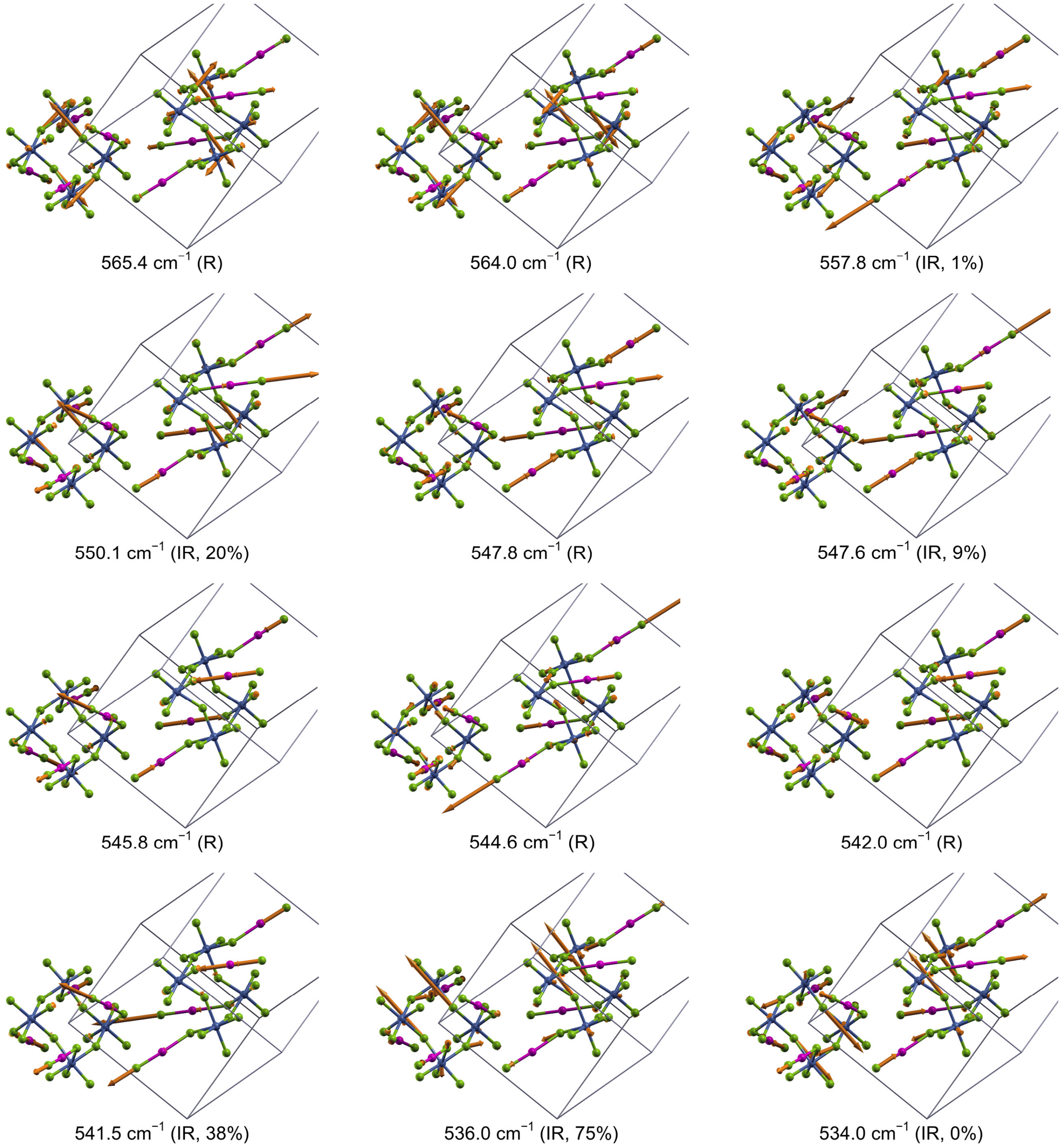

Figure S19 – continued.

$XeF_2 \cdot MnF_4$ – continued (part-4)

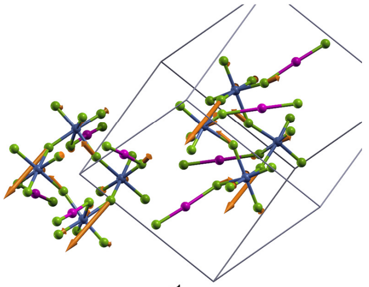

531.3 cm^{-1} (IR, 67%)

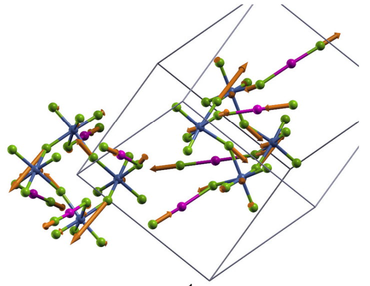

528.8 cm^{-1} (IR, 1%)

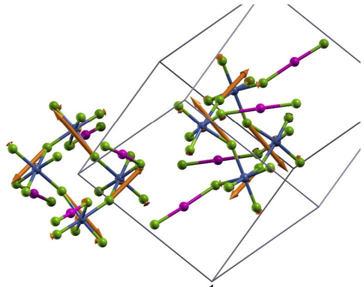

471.5 cm^{-1} (R)

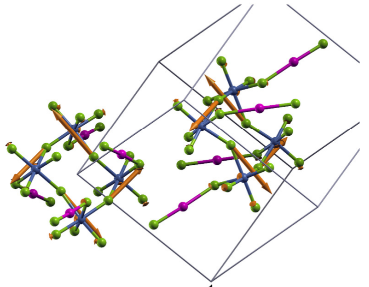

471.5 cm^{-1} (R)

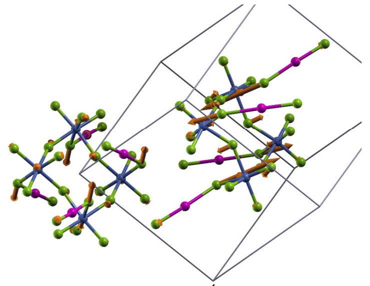

434.9 cm^{-1} (R)

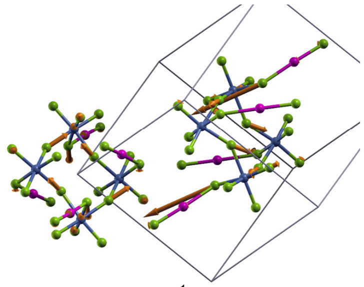

416.8 cm^{-1} (IR, 28%)

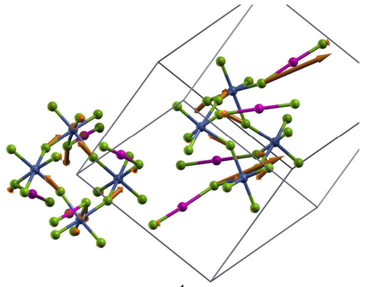

416.4 cm^{-1} (IR, 57%)

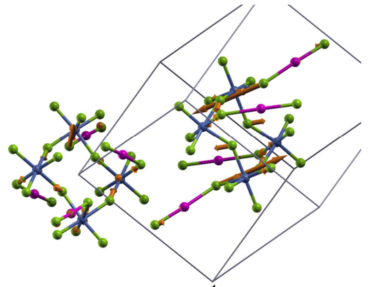

415.9 cm^{-1} (R)

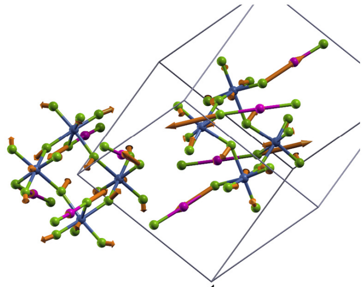

398.4 cm^{-1} (R)

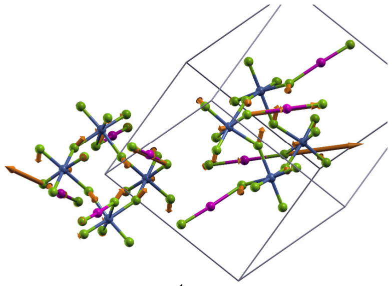

395.3 cm^{-1} (IR, 22%)

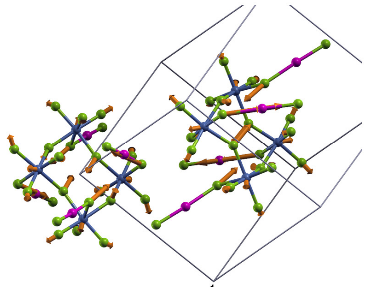

393.7 cm^{-1} (R)

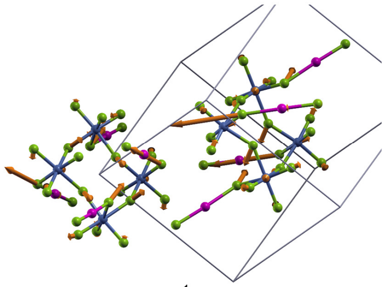

388.7 cm^{-1} (IR, 7%)

Figure S19 – continued.

XeF_2·MnF_4 – continued (part-5)

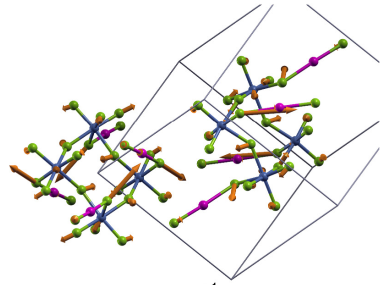

380.3 cm^{-1} (R)

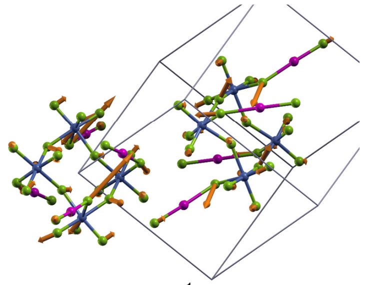

379.6 cm^{-1} (IR, 1%)

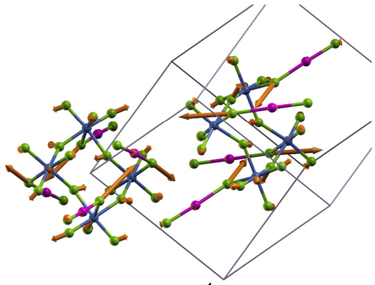

379.6 cm^{-1} (R)

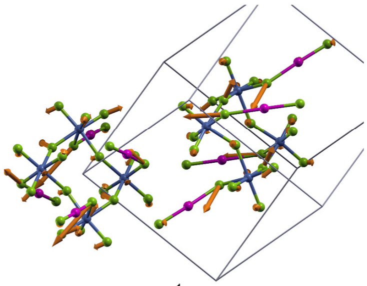

378.4 cm^{-1} (IR, 20%)

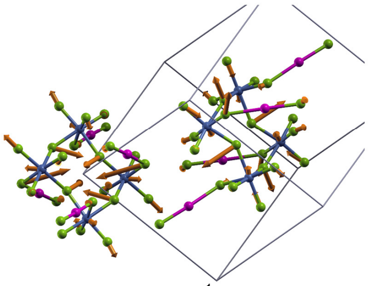

366.1 cm^{-1} (R)

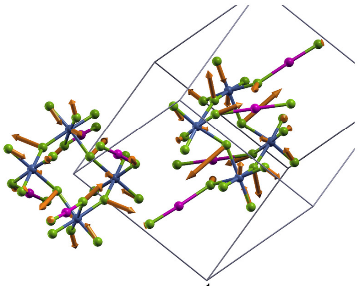

363.1 cm^{-1} (R)

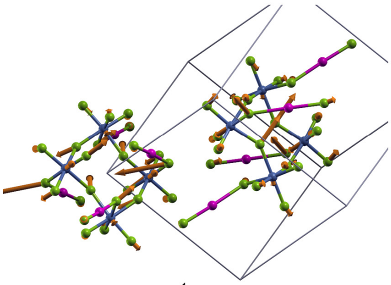

361.5 cm^{-1} (IR, 2%)

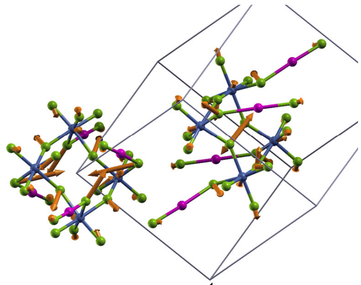

360.5 cm^{-1} (R)

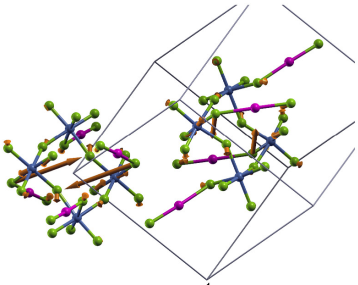

357.0 cm^{-1} (R)

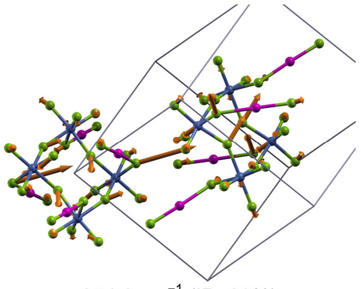

354.2 cm^{-1} (IR, 29%)

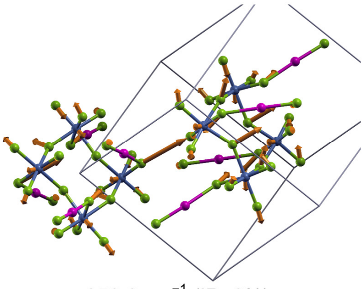

353.0 cm^{-1} (IR, 8%)

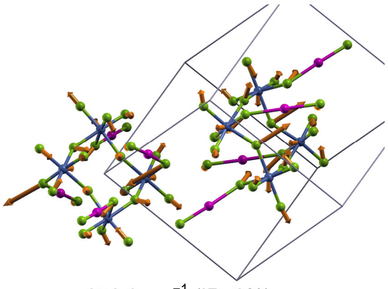

352.9 cm^{-1} (IR, 2%)

Figure S19 – continued.

$XeF_2 \cdot MnF_4$ – continued (part-6)

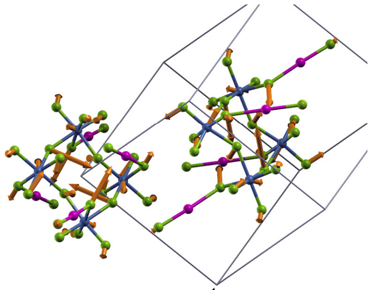

347.7 cm^{-1} (R)

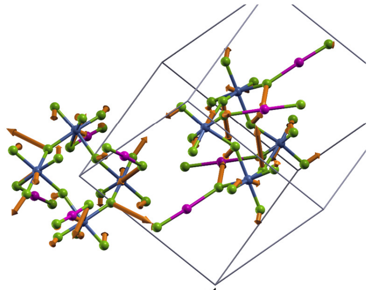

346.5 cm^{-1} (R)

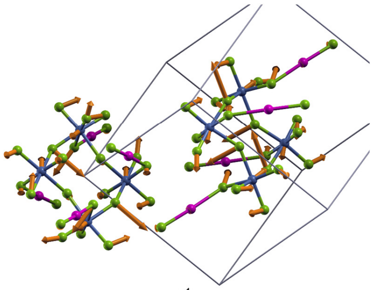

334.7 cm^{-1} (IR, 0%)

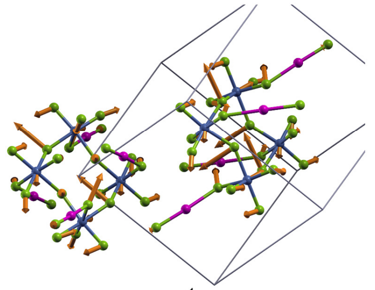

333.8 cm^{-1} (IR, 1%)

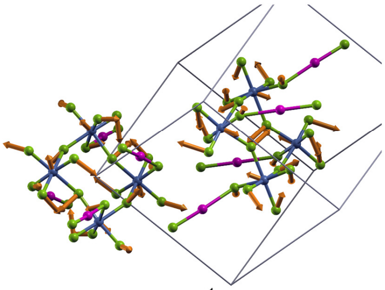

325.0 cm^{-1} (R)

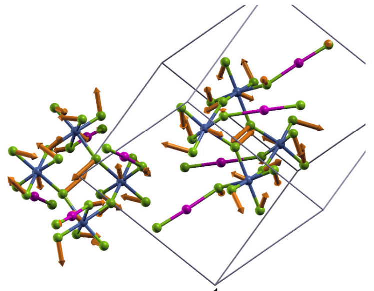

323.1 cm^{-1} (R)

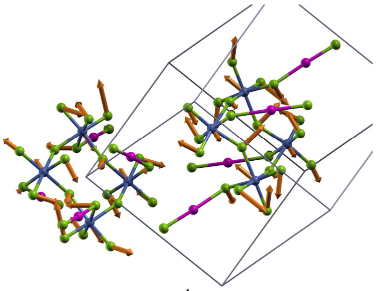

319.1 cm^{-1} (IR, 0%)

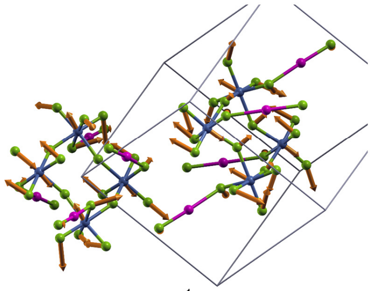

318.8 cm^{-1} (IR, 1%)

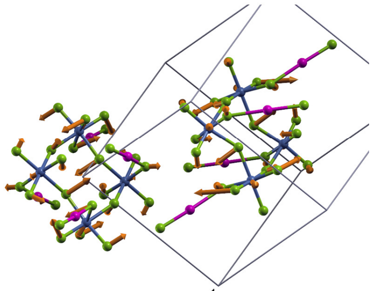

313.1 cm^{-1} (R)

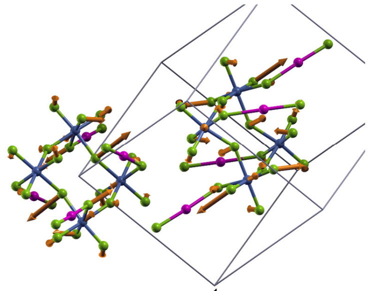

312.8 cm^{-1} (R)

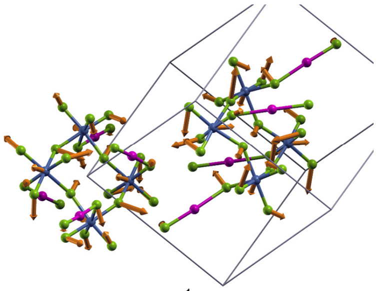

310.6 cm^{-1} (IR, 2%)

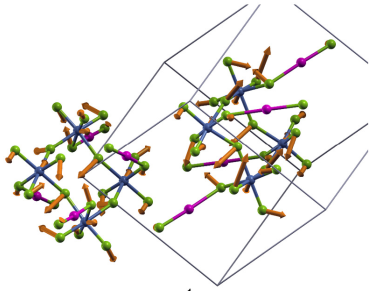

309.4 cm^{-1} (IR, 2%)

Figure S19 – continued.

$XeF_2 \cdot MnF_4$ – continued (part-7)

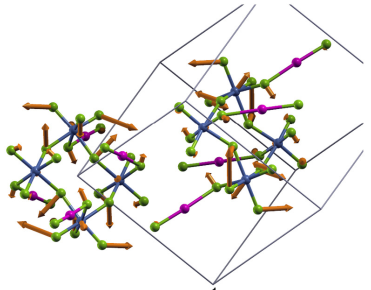
309.1 cm^{-1} (R)

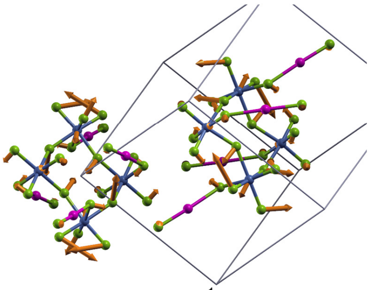
308.1 cm^{-1} (R)

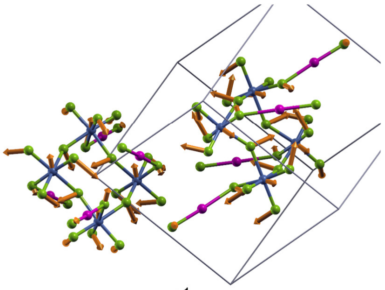
306.4 cm^{-1} (IR, 3%)

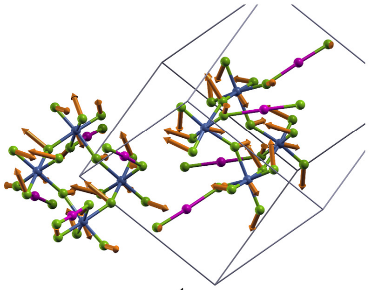
305.1 cm^{-1} (IR, 10%)

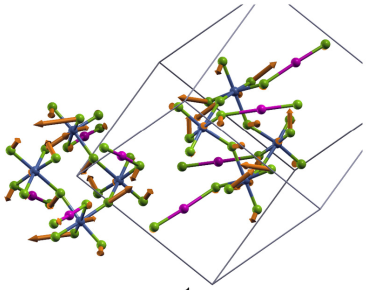
301.5 cm^{-1} (IR, 2%)

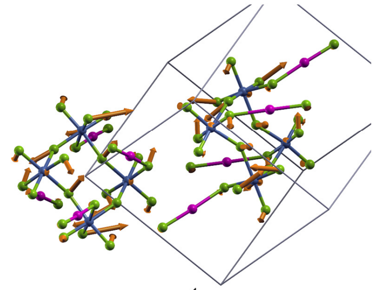
301.4 cm^{-1} (IR, 0%)

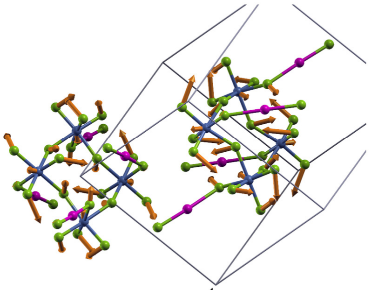
299.7 cm^{-1} (R)

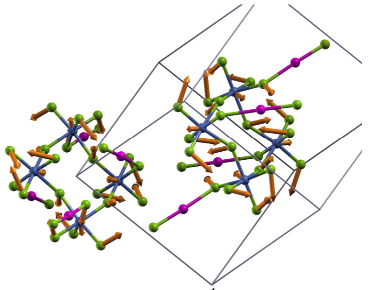
299.3 cm^{-1} (R)

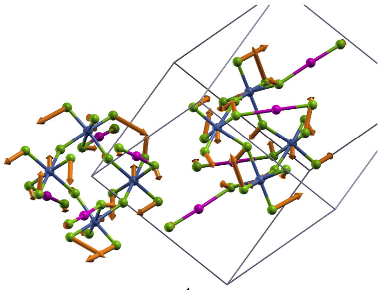
286.5 cm^{-1} (IR, 0%)

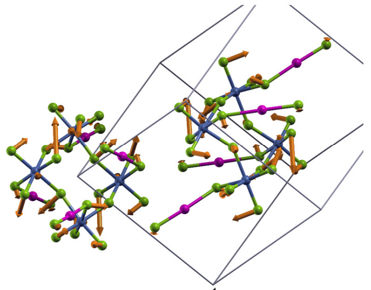
284.2 cm^{-1} (R)

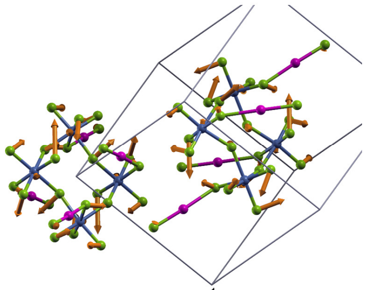
284.2 cm^{-1} (R)

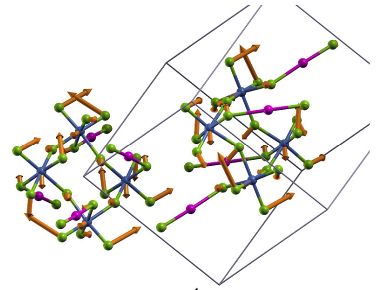
284.1 cm^{-1} (IR, 6%)

Figure S19 – continued.

$XeF_2 \cdot MnF_4$ – continued (part-8)

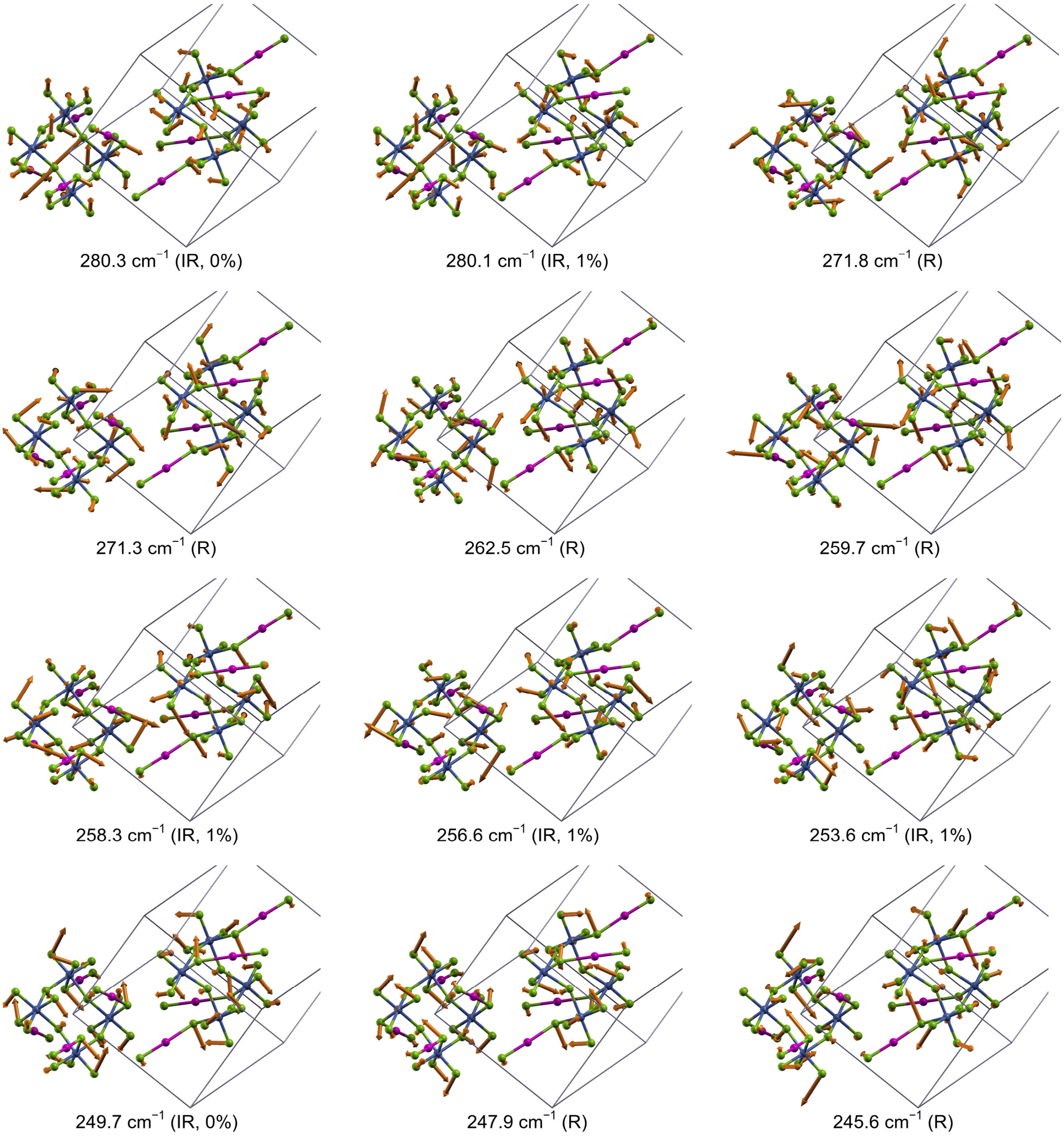

Figure S19 – continued.

$XeF_2 \cdot MnF_4$ – continued (part-9)

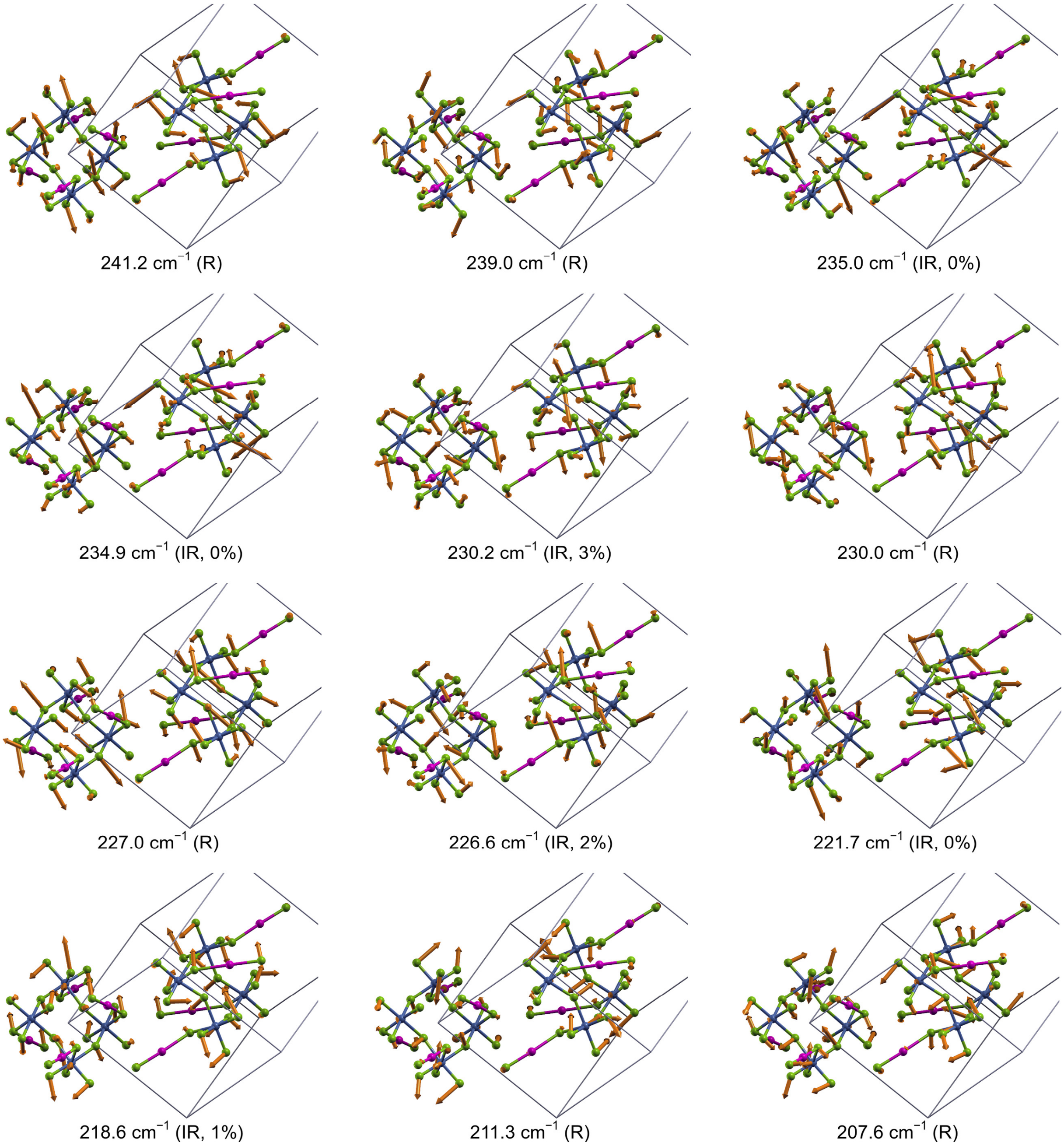

Figure S19 – continued.

$XeF_2 \cdot MnF_4$ – continued (part-10)

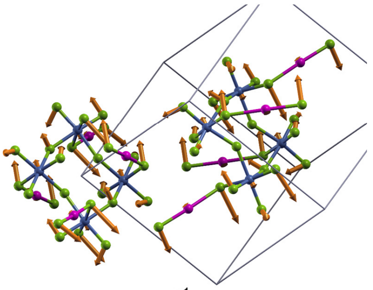
205.0 cm^{-1} (IR, 0%)

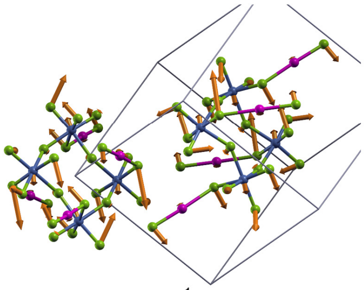
198.1 cm^{-1} (IR, 0%)

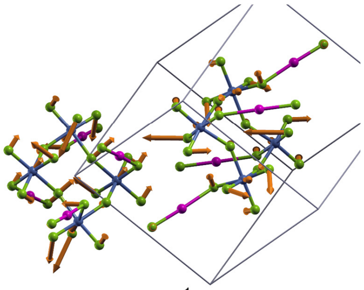
195.4 cm^{-1} (IR, 0%)

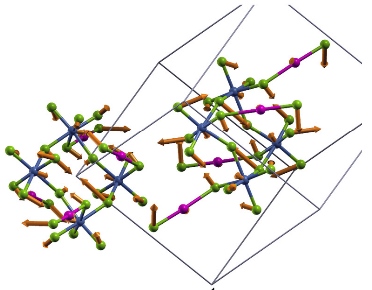
192.7 cm^{-1} (R)

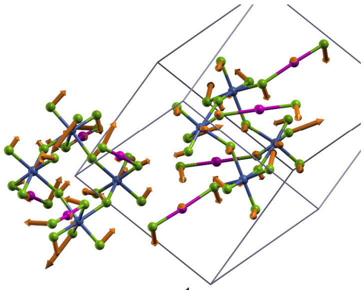
190.2 cm^{-1} (IR, 0%)

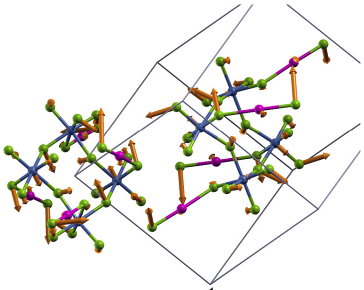
187.9 cm^{-1} (R)

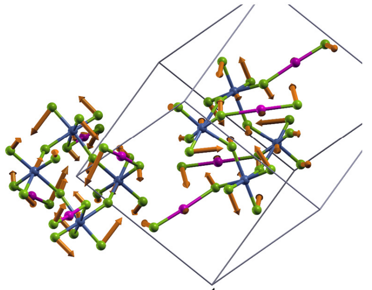
186.8 cm^{-1} (R)

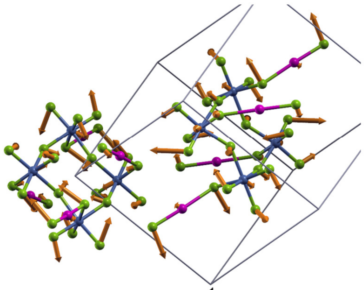
184.6 cm^{-1} (R)

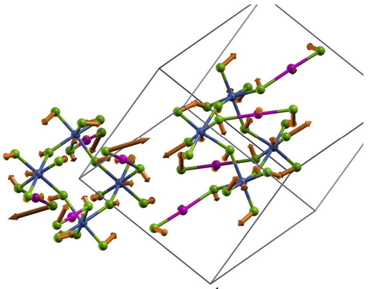
178.9 cm^{-1} (R)

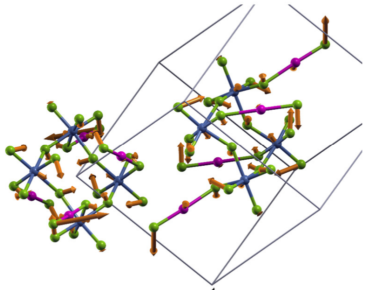
175.4 cm^{-1} (R)

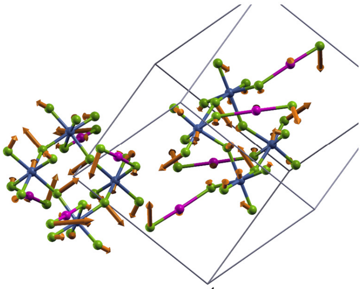
174.2 cm^{-1} (R)

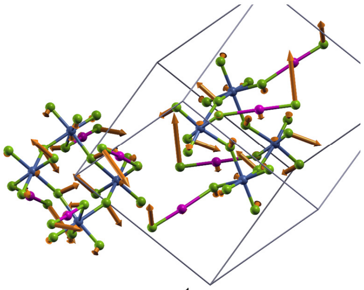
170.7 cm^{-1} (IR, 0%)

Figure S19 – continued.

$XeF_2 \cdot MnF_4$ – continued (part-11)

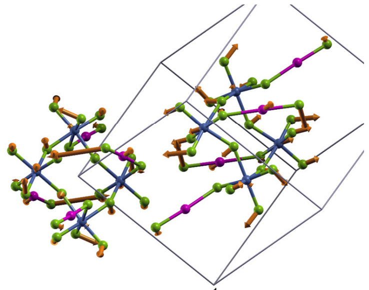

169.1 cm^{-1} (R)

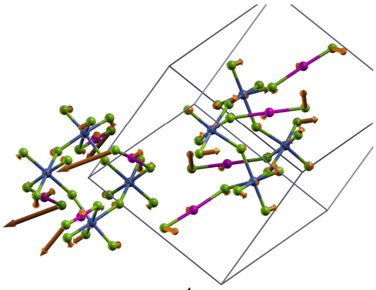

166.7 cm^{-1} (IR, 0%)

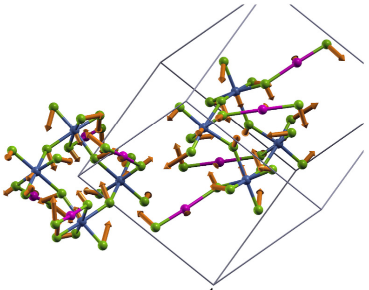

163.4 cm^{-1} (R)

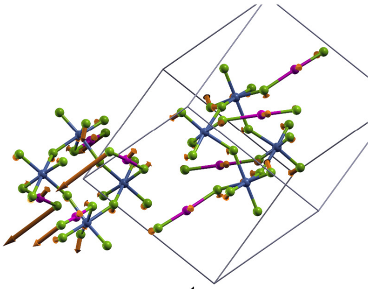

162.1 cm^{-1} (IR, 7%)

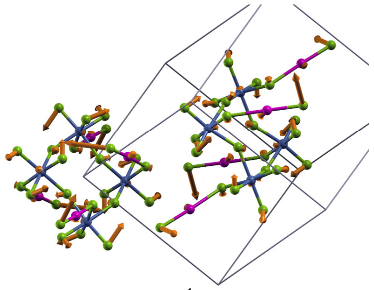

158.3 cm^{-1} (IR, 0%)

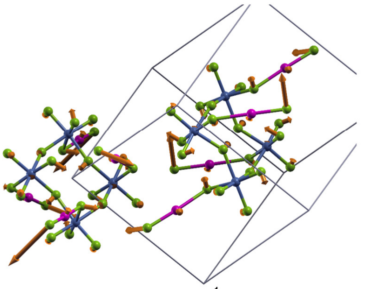

158.3 cm^{-1} (R)

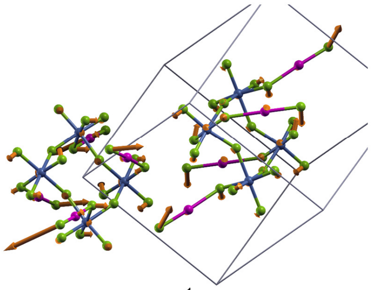

157.0 cm^{-1} (IR, 1%)

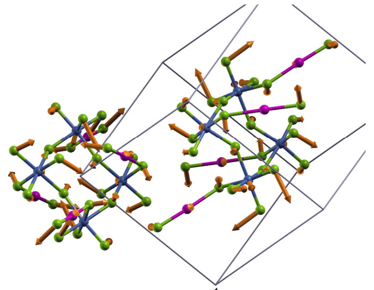

155.0 cm^{-1} (R)

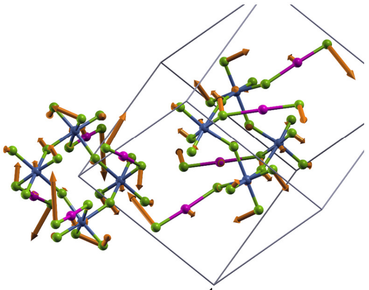

154.2 cm^{-1} (R)

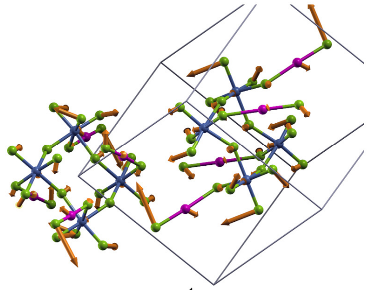

151.2 cm^{-1} (IR, 0%)

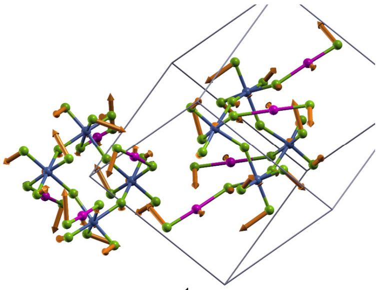

150.6 cm^{-1} (IR, 0%)

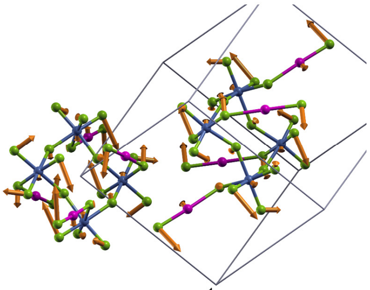

149.0 cm^{-1} (R)

Figure S19 – continued.

$XeF_2 \cdot MnF_4$ – continued (part-12)

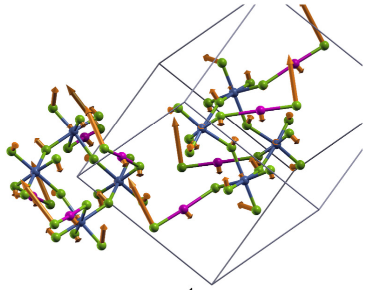

147.2 cm^{-1} (IR, 4%)

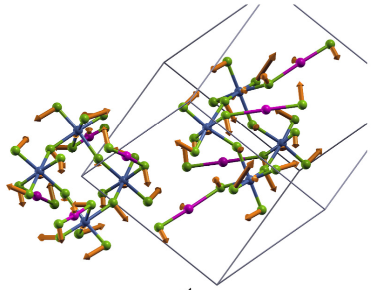

142.3 cm^{-1} (IR, 0%)

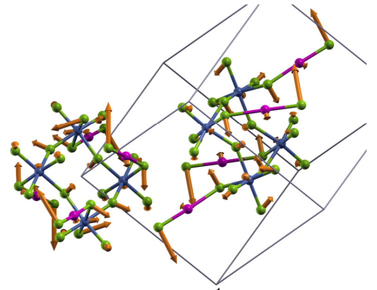

141.1 cm^{-1} (R)

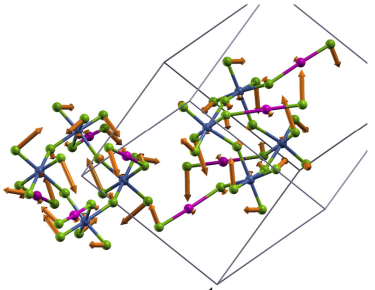

140.6 cm^{-1} (R)

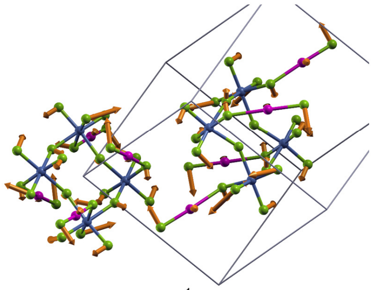

137.4 cm^{-1} (IR, 1%)

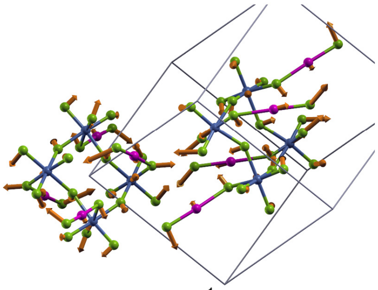

135.0 cm^{-1} (R)

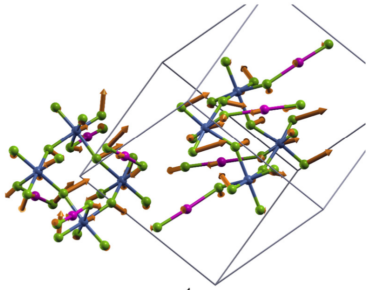

134.9 cm^{-1} (IR, 2%)

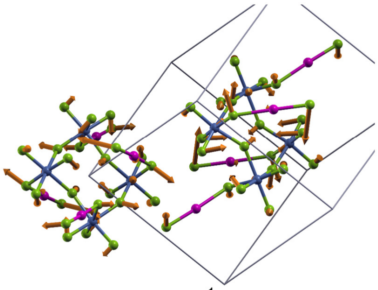

130.7 cm^{-1} (R)

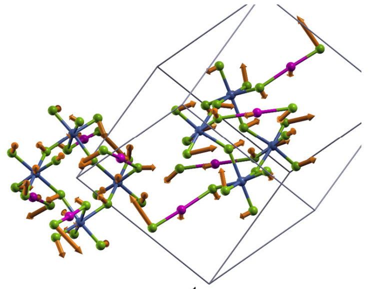

129.5 cm^{-1} (IR, 1%)

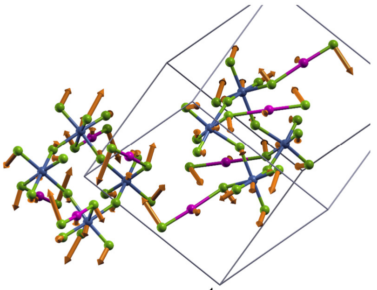

127.7 cm^{-1} (R)

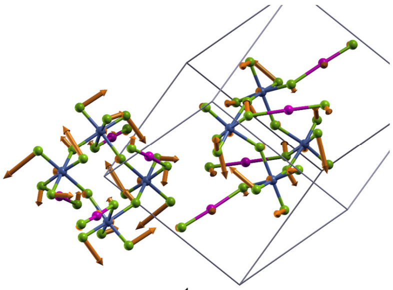

121.3 cm^{-1} (IR, 0%)

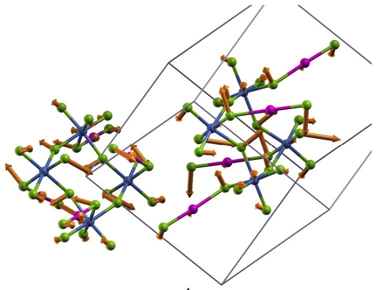

115.3 cm^{-1} (IR, 0%)

Figure S19 – continued.

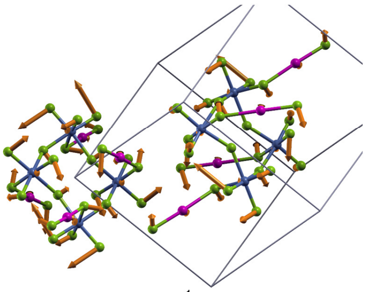

109.3 cm^{-1} (IR, 1%)

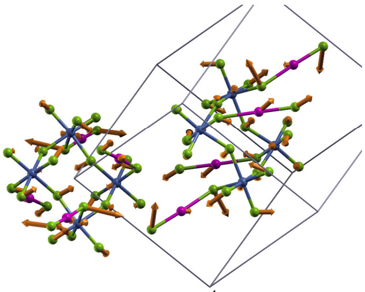

107.4 cm^{-1} (R)

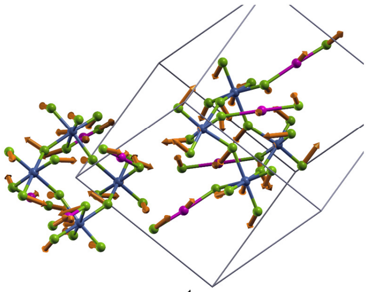

106.4 cm^{-1} (IR, 1%)

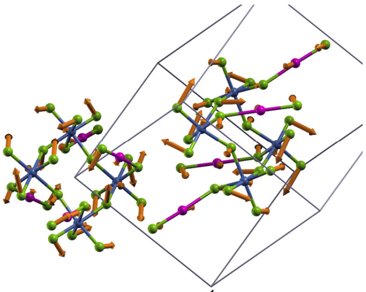

104.5 cm^{-1} (R)

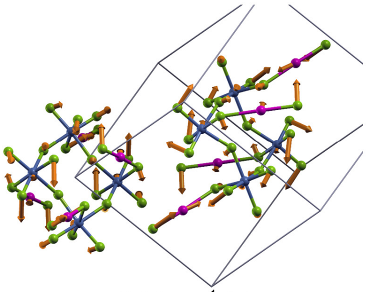

104.3 cm^{-1} (R)

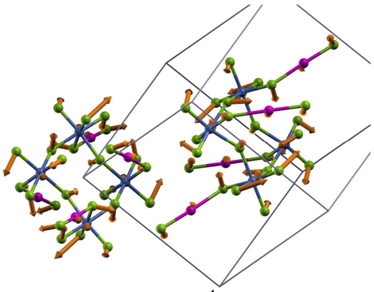

100.8 cm^{-1} (R)

Figure S19 – continued.

$XeF_2 \cdot 2MnF_4$

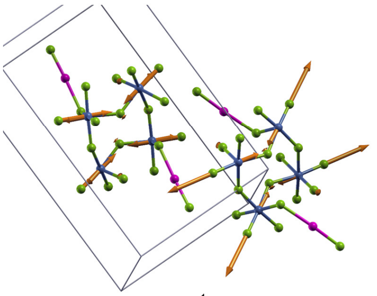

710.5 cm^{-1} (R)

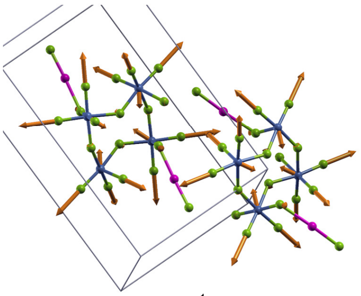

708.5 cm^{-1} (R)

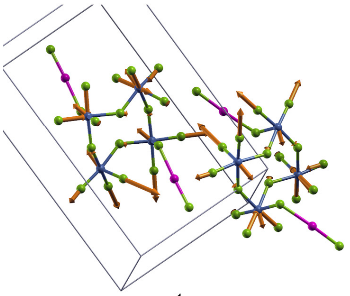

707.3 cm^{-1} (IR, 1%)

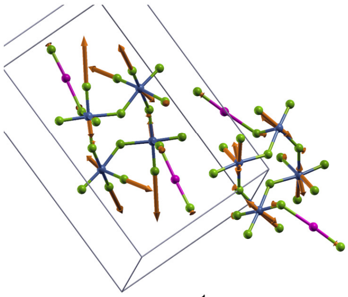

704.3 cm^{-1} (R)

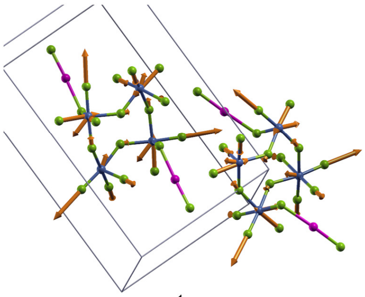

694.9 cm^{-1} (IR, 1%)

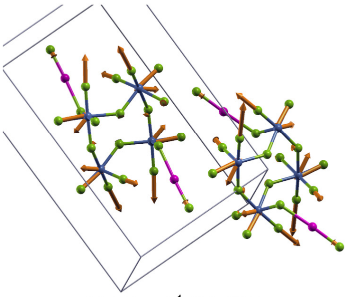

689.7 cm^{-1} (IR, 0%)

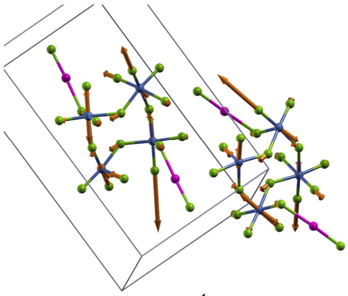

689.6 cm^{-1} (R)

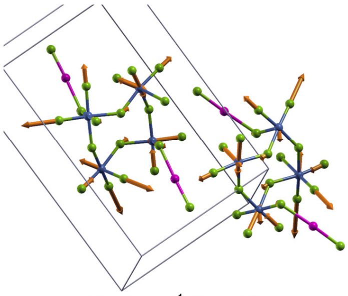

689.0 cm^{-1} (IR, 2%)

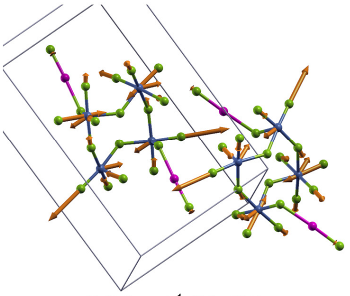

679.8 cm^{-1} (IR, 0%)

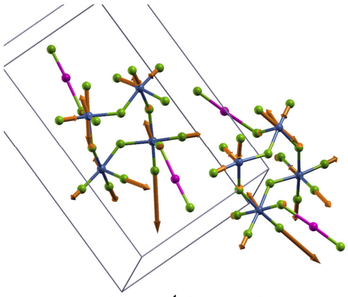

674.3 cm^{-1} (IR, 56%)

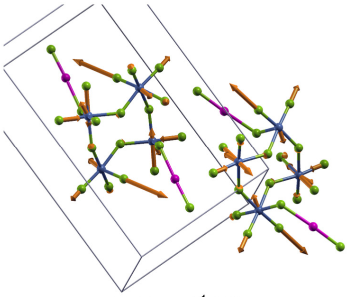

674.0 cm^{-1} (R)

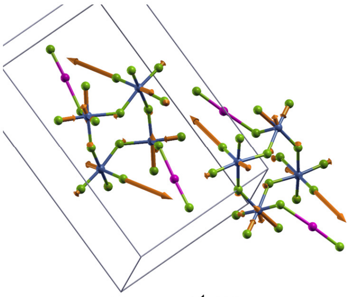

667.7 cm^{-1} (R)

Figure S20. DFT/PBE-D calculated vibrational modes of $XeF_2 \cdot 2MnF_4$ with frequencies above 100 cm^{-1} (sorted from high to low frequencies). The (IR) and (R) labels indicate whether a mode is IR or Raman active; for the IR active modes, normalized intensities (as percentages) are also stated. The figure continues on the next pages.

$XeF_2{\cdot}2MnF_4$ – continued (part-2)

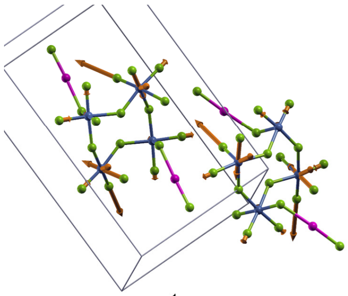

664.4 cm^{-1} (IR, 41%)

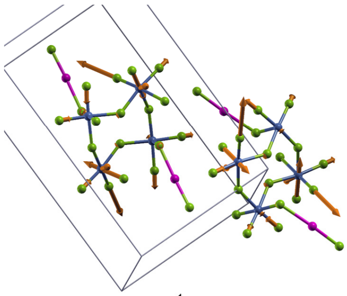

663.9 cm^{-1} (IR, 1%)

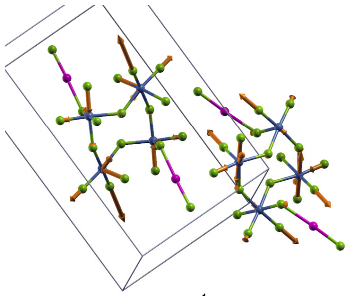

663.1 cm^{-1} (R)

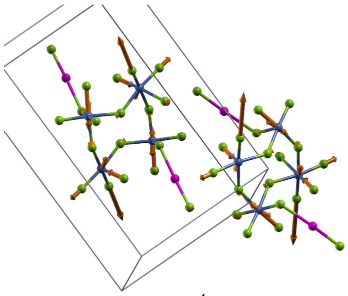

660.8 cm^{-1} (R)

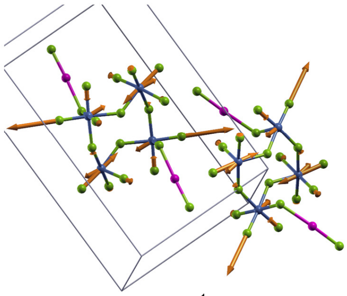

653.3 cm^{-1} (R)

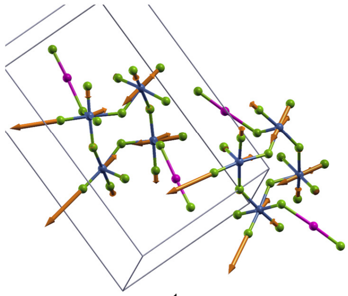

651.7 cm^{-1} (IR, 84%)

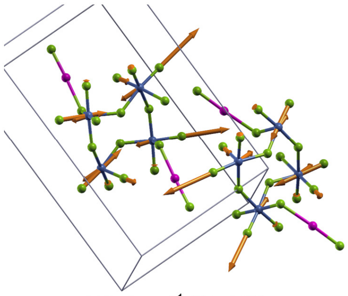

650.6 cm^{-1} (IR, 8%)

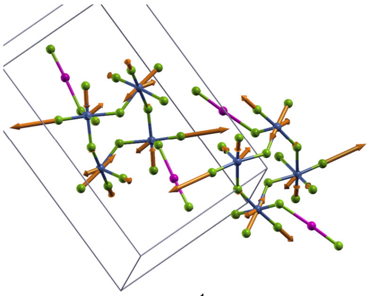

649.6 cm^{-1} (R)

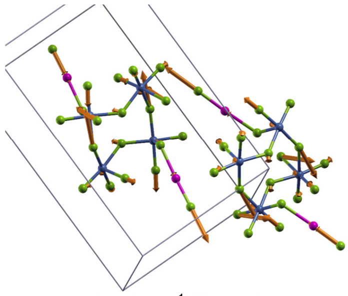

592.2 cm^{-1} (IR, 1%)

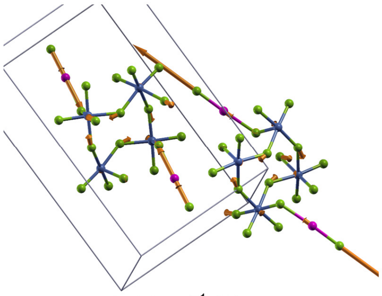

591.0 cm^{-1} (R)

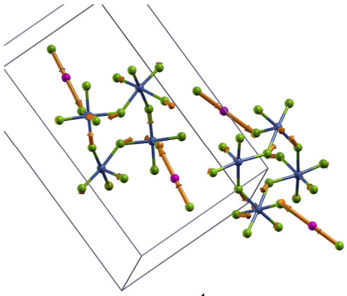

585.0 cm^{-1} (R)

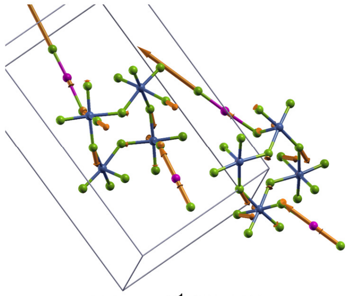

584.0 cm^{-1} (IR, 0%)

Figure S20 – continued.

$XeF_2 \cdot 2MnF_4$ – continued (part-3)

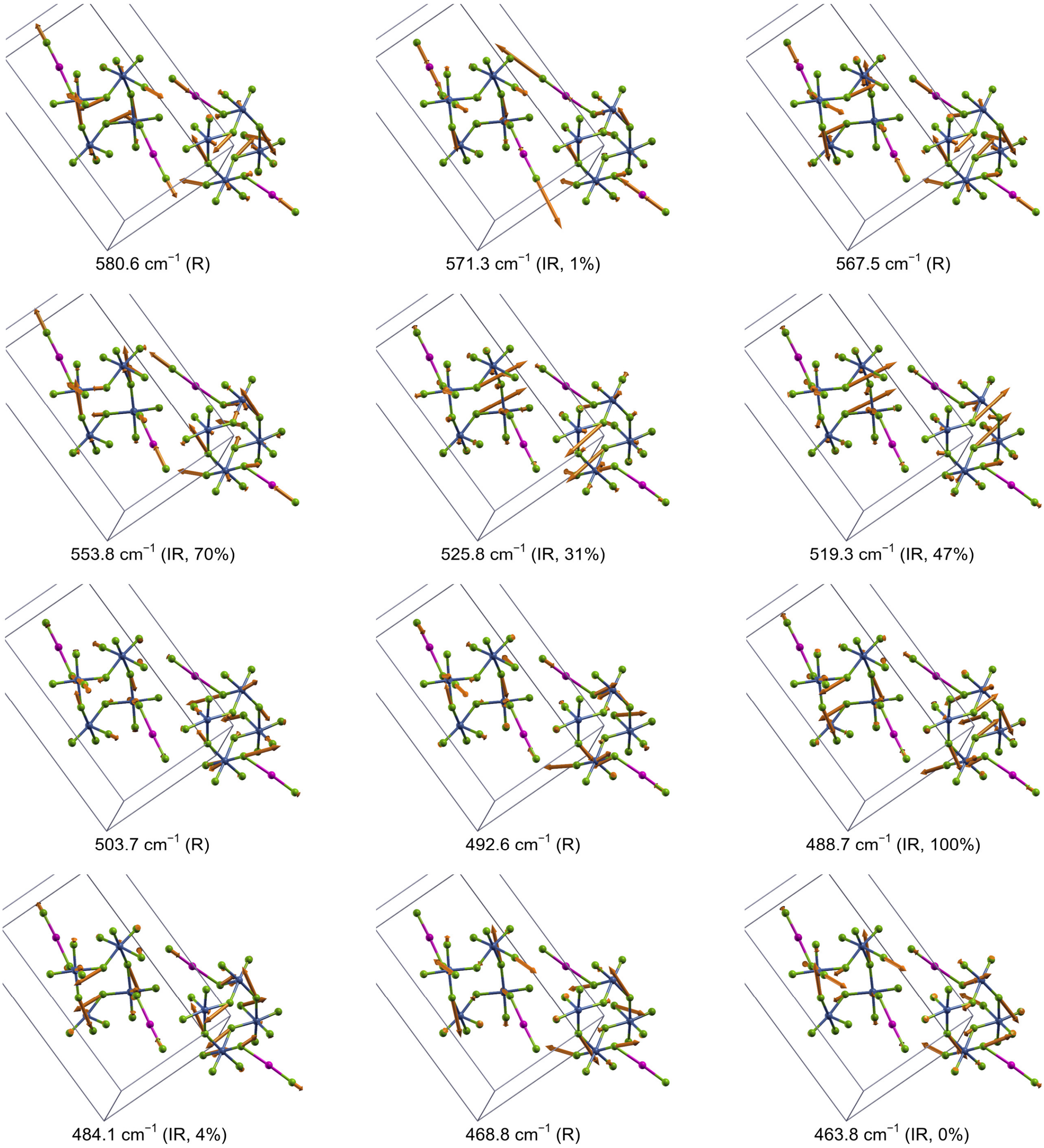

Figure S20 – continued.

$XeF_2 \cdot 2MnF_4$ – continued (part-4)

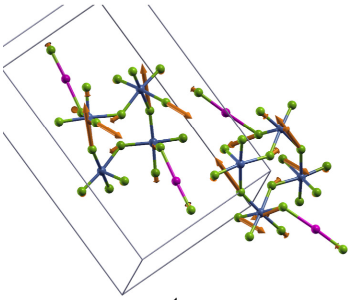

462.9 cm^{-1} (IR, 24%)

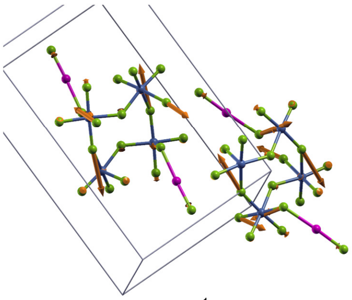

462.3 cm^{-1} (R)

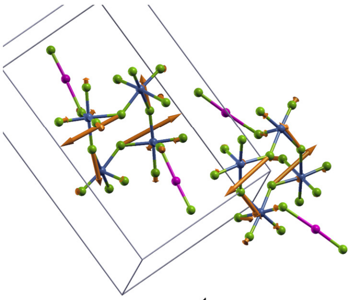

455.4 cm^{-1} (R)

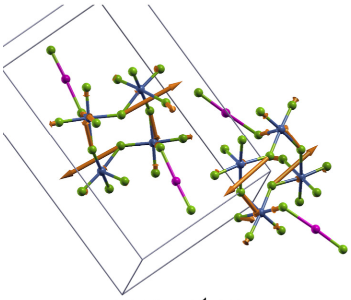

455.4 cm^{-1} (R)

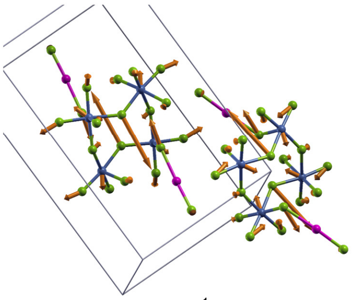

400.9 cm^{-1} (R)

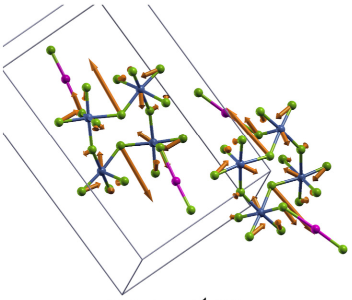

399.6 cm^{-1} (R)

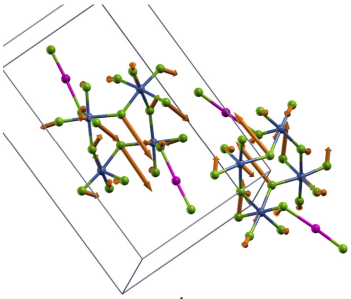

380.2 cm^{-1} (IR, 0%)

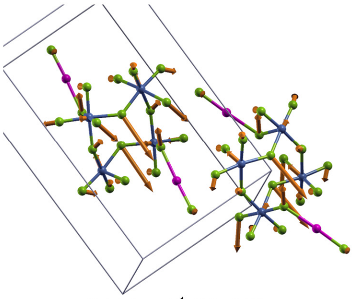

377.9 cm^{-1} (IR, 3%)

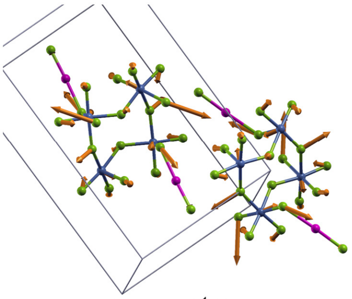

376.4 cm^{-1} (R)

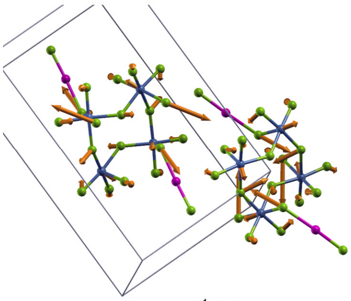

375.8 cm^{-1} (R)

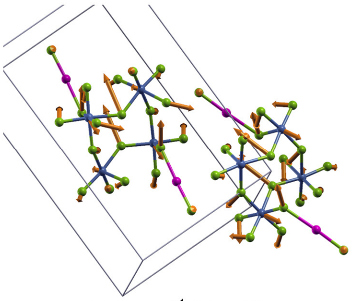

367.8 cm^{-1} (IR, 1%)

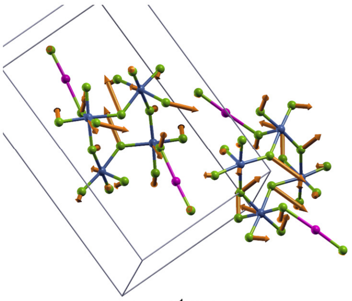

367.4 cm^{-1} (IR, 2%)

Figure S20 – continued.

XeF_2·$2MnF_4$ – continued (part-5)

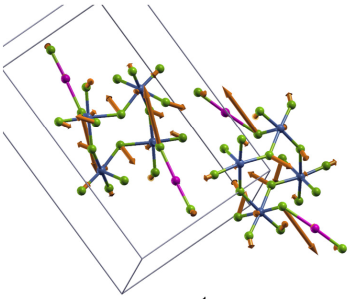

365.4 cm^{-1} (R)

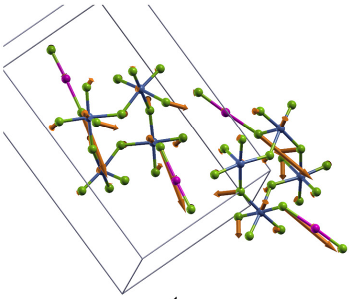

363.0 cm^{-1} (IR, 20%)

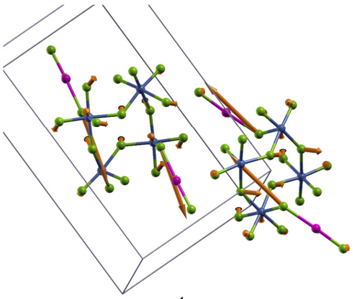

362.5 cm^{-1} (IR, 0%)

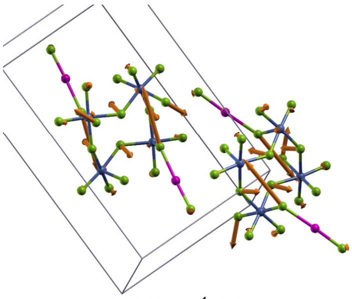

360.3 cm^{-1} (R)

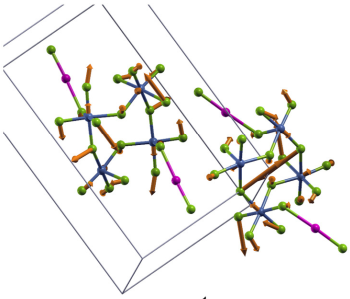

347.8 cm^{-1} (R)

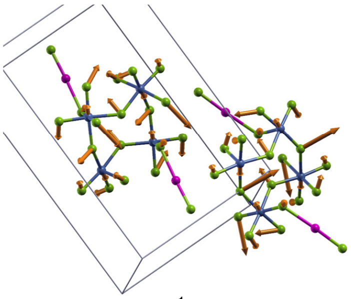

345.8 cm^{-1} (IR, 2%)

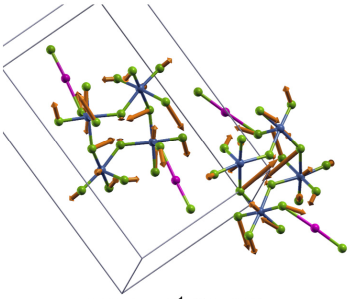

345.2 cm^{-1} (IR, 0%)

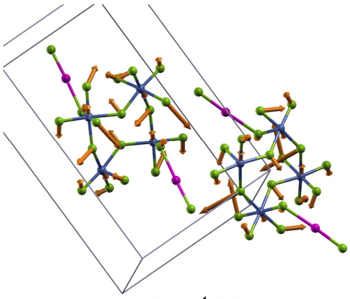

345.2 cm^{-1} (R)

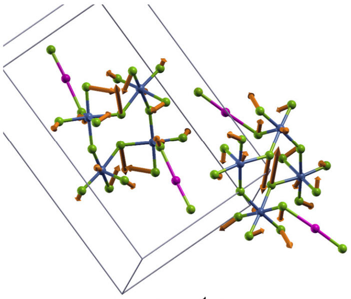

336.7 cm^{-1} (R)

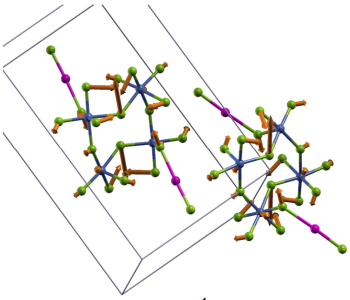

335.6 cm^{-1} (R)

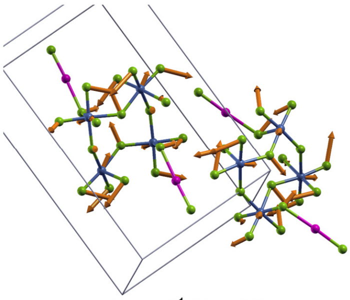

325.2 cm^{-1} (IR, 1%)

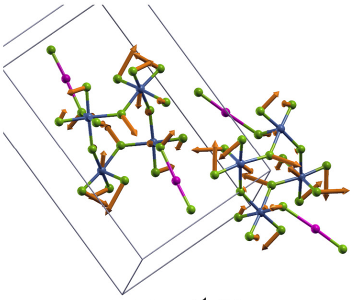

323.3 cm^{-1} (R)

Figure S20 – continued.

$XeF_2 \cdot 2MnF_4$ – continued (part-6)

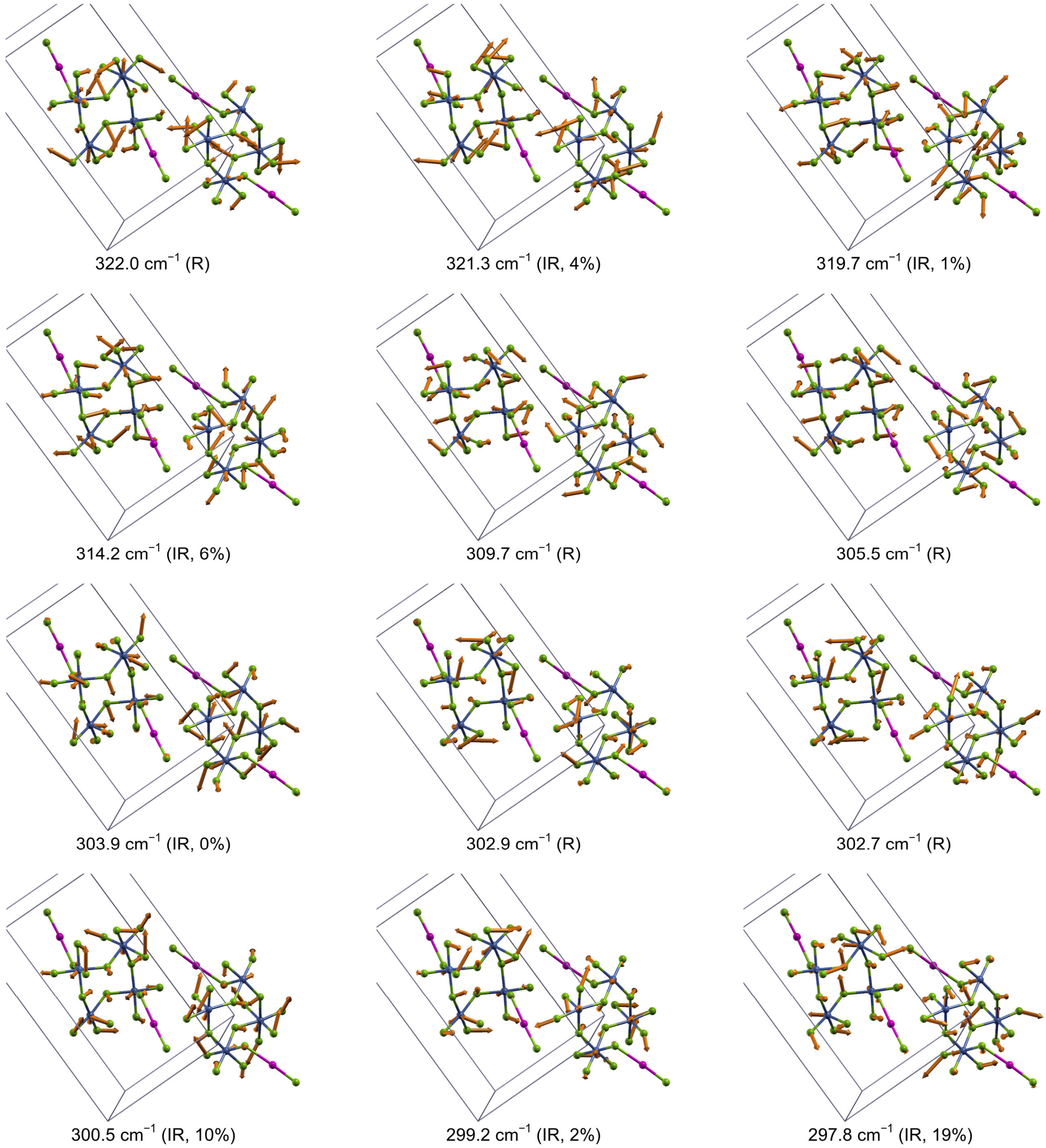

Figure S20 – continued.

XeF_2·$2MnF_4$ – continued (part-7)

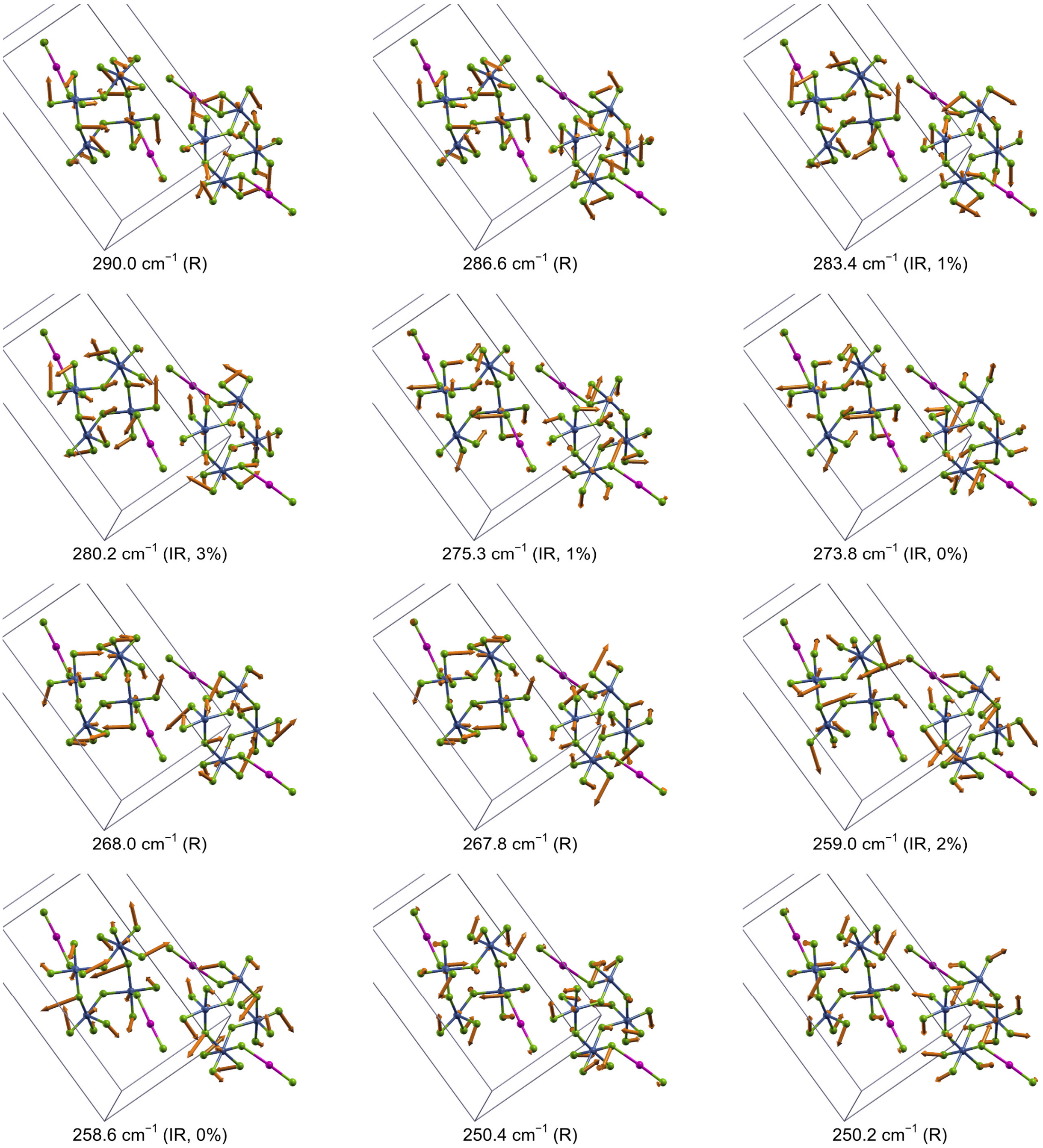

Figure S20 – continued.

$XeF_2·2MnF_4$ – continued (part-8)

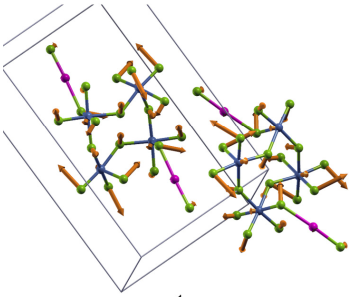

249.2 cm^{-1} (IR, 0%)

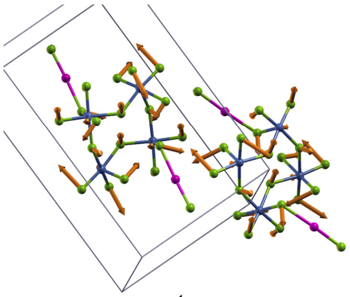

249.0 cm^{-1} (IR, 2%)

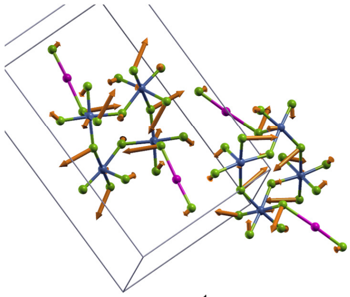

244.9 cm^{-1} (R)

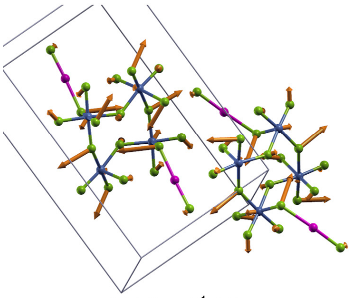

244.2 cm^{-1} (R)

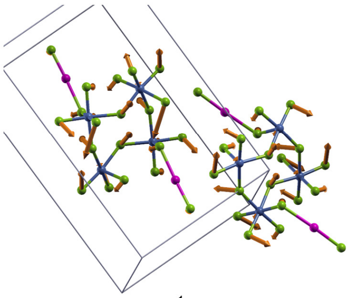

235.3 cm^{-1} (IR, 0%)

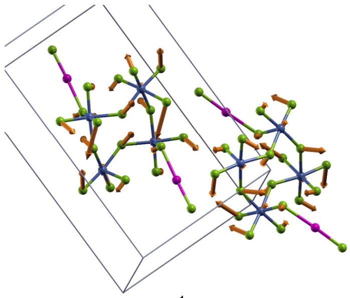

234.9 cm^{-1} (IR, 0%)

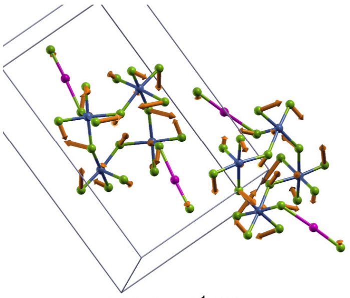

229.0 cm^{-1} (R)

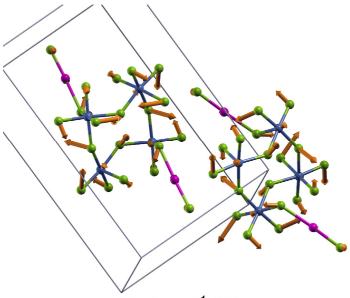

228.9 cm^{-1} (R)

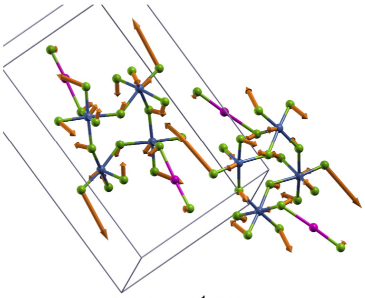

220.0 cm^{-1} (R)

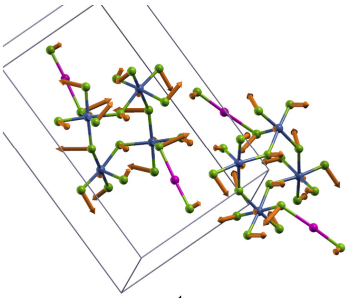

218.7 cm^{-1} (IR, 0%)

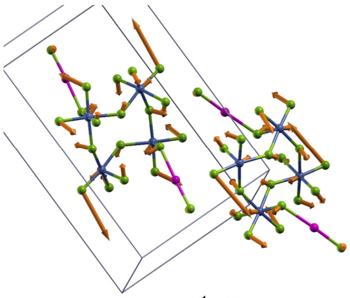

216.9 cm^{-1} (R)

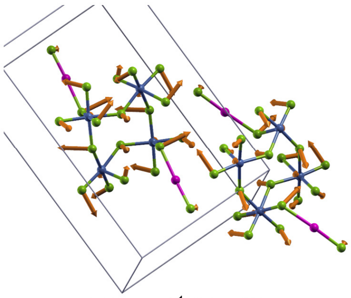

216.5 cm^{-1} (IR, 0%)

Figure S20 – continued.

XeF_2·$2MnF_4$ – continued (part-9)

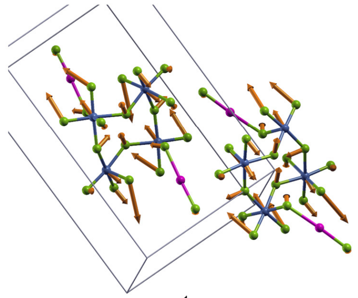

201.3 cm^{-1} (IR, 3%)

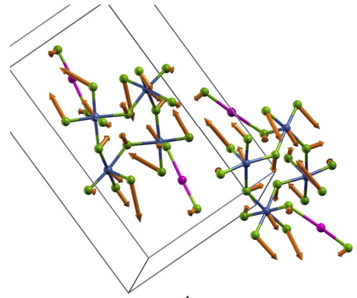

199.2 cm^{-1} (IR, 0%)

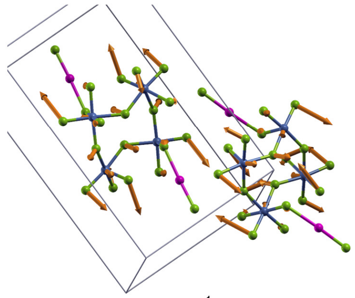

190.5 cm^{-1} (R)

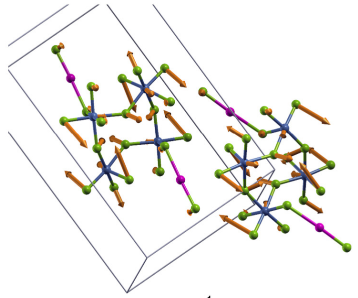

190.4 cm^{-1} (R)

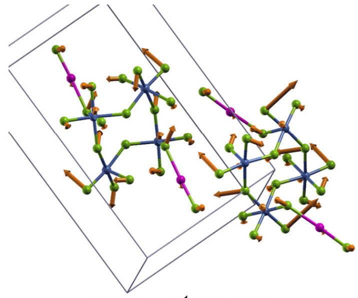

182.8 cm^{-1} (IR, 2%)

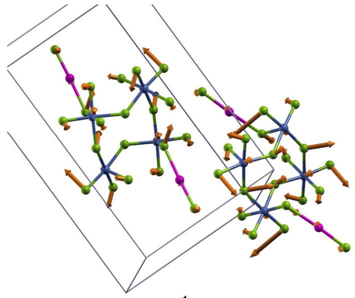

180.3 cm^{-1} (IR, 0%)

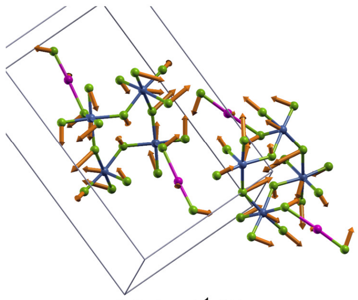

176.6 cm^{-1} (R)

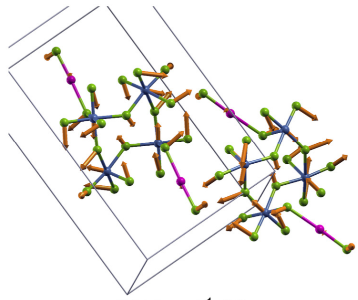

175.8 cm^{-1} (R)

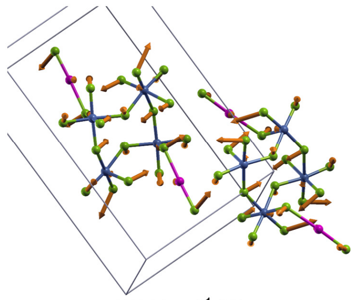

171.7 cm^{-1} (R)

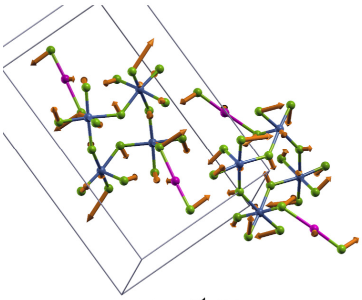

169.2 cm^{-1} (R)

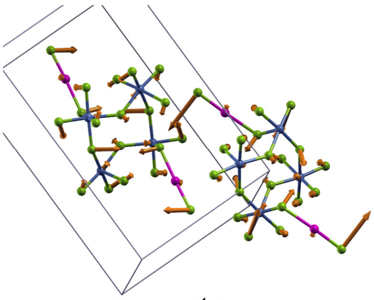

163.3 cm^{-1} (R)

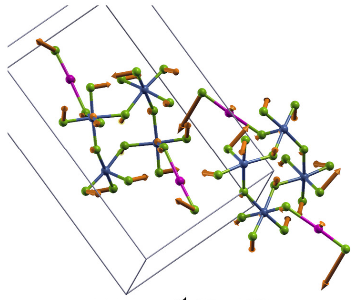

161.2 cm^{-1} (IR, 0%)

Figure S20 – continued.

$XeF_2 \cdot 2MnF_4$ – continued (part-10)

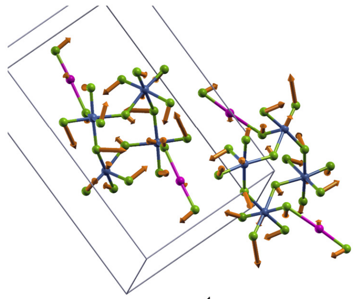
161.2 cm^{-1} (R)

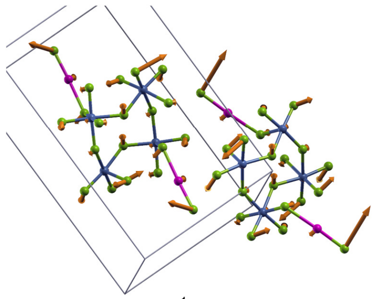
159.1 cm^{-1} (IR, 1%)

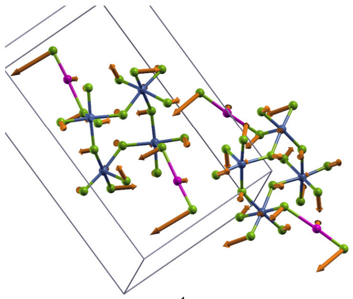
155.2 cm^{-1} (IR, 1%)

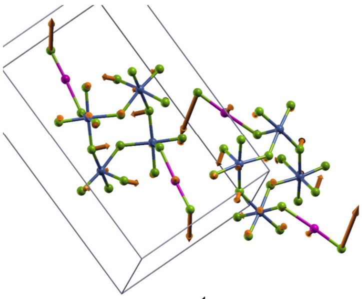
153.0 cm^{-1} (R)

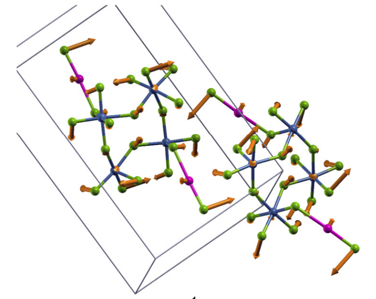
150.8 cm^{-1} (IR, 0%)

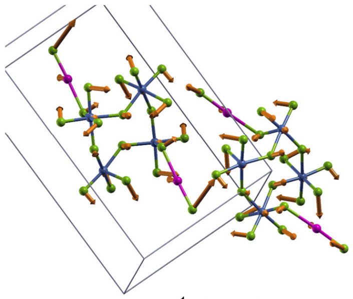
148.3 cm^{-1} (IR, 2%)

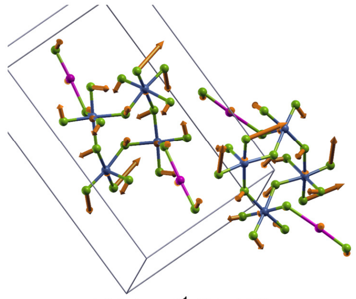
146.4 cm^{-1} (IR, 0%)

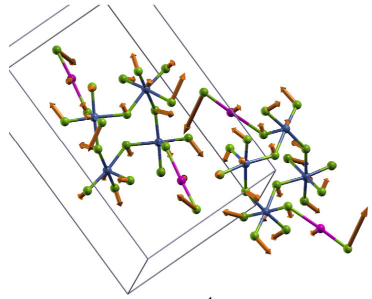
145.5 cm^{-1} (R)

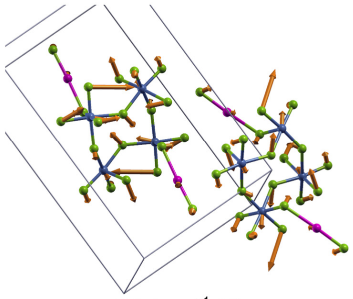
139.3 cm^{-1} (R)

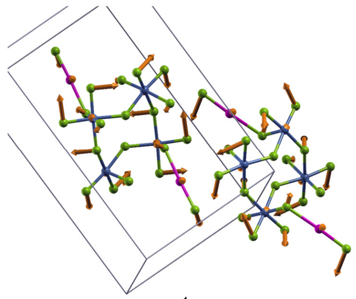
137.9 cm^{-1} (IR, 1%)

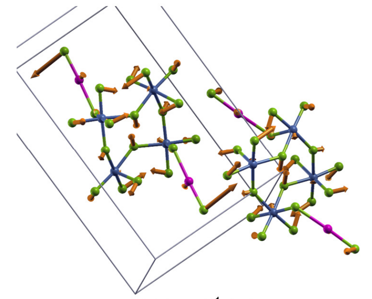
137.6 cm^{-1} (R)

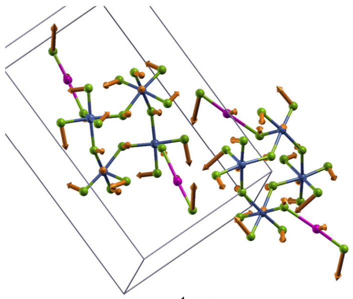
137.2 cm^{-1} (IR, 0%)

Figure S20 – continued.

$XeF_2 \cdot 2MnF_4$ – continued (part-11)

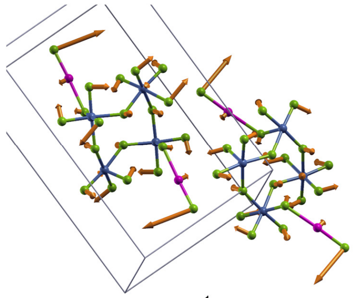

132.9 cm^{-1} (R)

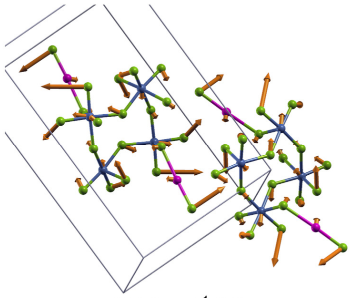

131.9 cm^{-1} (R)

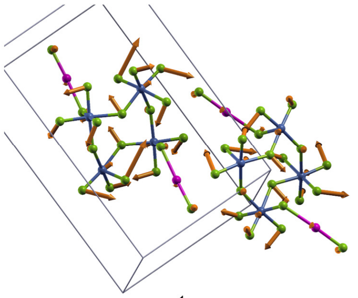

122.3 cm^{-1} (IR, 0%)

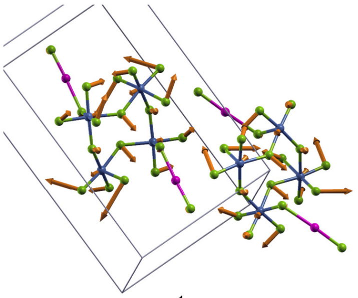

120.1 cm^{-1} (IR, 0%)

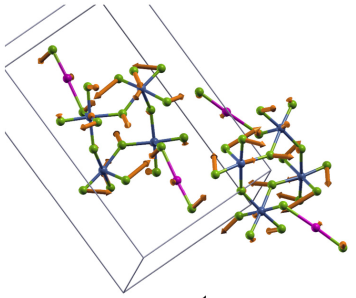

118.2 cm^{-1} (R)

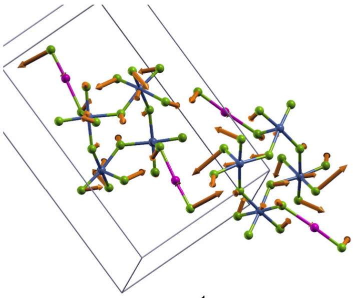

117.0 cm^{-1} (R)

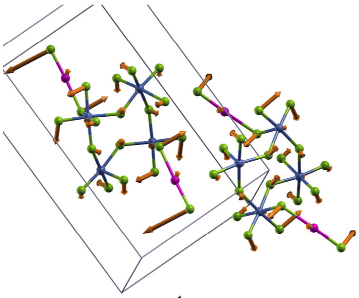

116.8 cm^{-1} (IR, 1%)

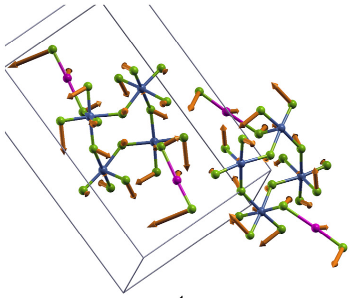

114.1 cm^{-1} (IR, 1%)

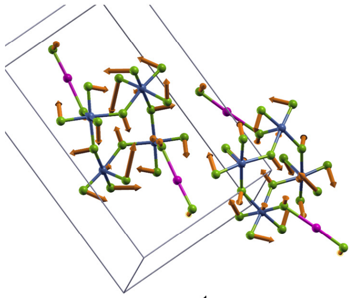

113.1 cm^{-1} (R)

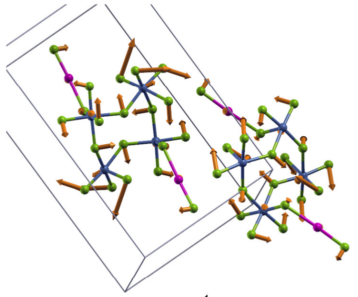

113.0 cm^{-1} (R)

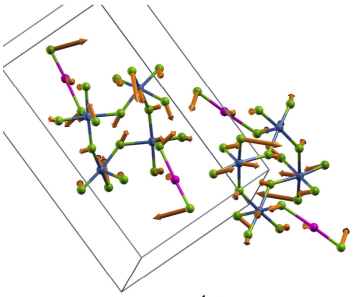

103.0 cm^{-1} (R)

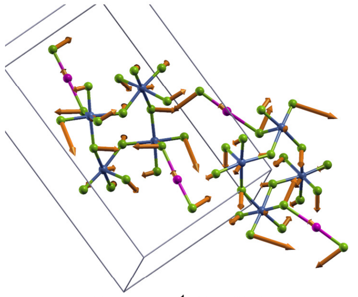

101.9 cm^{-1} (IR, 1%)

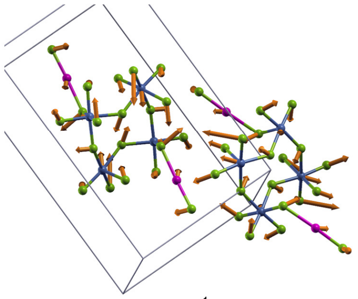

101.3 cm^{-1} (R)

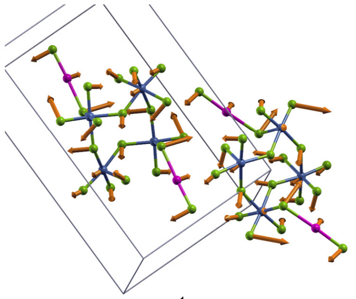

100.0 cm^{-1} (IR, 1%)

Figure S20 – continued.

References:

[S1] Saha, A.; Nia, S. S.; Rodríguez, J. A. Electron Diffraction of 3D Molecular Crystals. *Chem. Rev.* **2022**, *122* (17), 13883–13914.
https://doi.org/10.1021/acs.chemrev.1c00879.

[S2] Brown, I. D. The Chemical Bond in Inorganic Chemistry – The Bond Valence Model, 2nd Edition, IUCr Monographs on Crystallography, Oxford University Press: New York, **2016**.
https://doi.org/10.1093/acprof:oso/9780198742951.001.0001

[S3] Brown, I. D. Recent Developments in the Methods and Applications of the Bond Valence Model. *Chem. Rev.* **2009**, *109* (12), 6858–6919.
https://doi.org/10.1021/cr900053k

[S4] a) Brown, I. D. bvparam2020.cif, Accumulated Table of Bond Valence Parameters, (IUCr) Bond valence parameters, Brockhouse Institute for Materials Research, McMaster University, Hamilton, ON, Canada.
http://www.iucr.org/resources/data/datasets/bond-valence-parameters (2024)

b) Brown, I. D.; Altermatt, D. Bond-Valence Parameters for Solids. *Acta Crystallogr. B* **1985**, *41*, 244–247.
https://doi.org/10.1107/S0108768185002063

[S5] Alvarez, S. A cartography of the van der Waals territories. *Dalton Trans.* **2013**, *42* (24), 8617–8636.
https://doi.org/10.1039/C3DT50599E

[S6] Šmalc, A.; Lutar, K.; Kinkead, S. A. Xenon Difluoride (Modification). In *Inorganic Syntheses,* Vol. 29; Grimes, R. N., Ed.; John Wiley & Sons: New York, **1992**. pp 1–4.
https://doi.org/10.1002/9780470132609.ch1

[S7] Mazej, Z. Room temperature syntheses of MnF_3, MnF_4 and hexafluoromanganete(IV) salts of alkali cations. *J. Fluorine Chem.* **2002**, *114* (1), 75–80.
https://doi.org/10.1016/S0022-1139(01)00566-8

[S8] Gemmi, M.; Mugnaioli, E.; Gorelik, T. E.; Kolb, U.; Palatinus, L.; Boullay, P.; Hovmöller, S.; Abrahams, J. P. 3D Electron Diffraction: The Nanocrystallography Revolution. *ACS Cent. Sci.* **2019**, *5* (8), 1315–1329.
https://doi.org/10.1021/acscentsci.9b00394

[S9] Palatinus, L.; Brázda, P.; Jelínek, M.; Hrdá, J.; Steciuk, G.; Klementová, M. Specifics of the Data Processing of Precession Electron Diffraction Tomography Data and Their Implementation in the Program *PETS2.0*. *Acta Crystallogr. B* **2019**, *75* (4), 512–522.
https://doi.org/10.1107/S2052520619007534

[S10] Petříček, V.; Dušek, M.; Palatinus, L. Crystallographic Computing System JANA2006: General features. *Z. Kristallogr. - Cryst. Mater.* **2014**, *229* (5), 345–352.
https://doi.org/10.1515/zkri-2014-1737

[S11] Burla, M. C.; Caliandro, R.; Carrozzini, B.; Cascarano, G. L.; Cuocci, C.; Giacovazzo, C.; Mallamo, M.; Mazzone, A.; Polidori, G. Crystal Structure Determination and Refinement via *SIR2014*. *J. Appl. Crystallogr.* **2015**, *48* (1), 306–309. https://doi.org/10.1107/S1600576715001132

[S12] Veith, M.; Bärnighausen H. Die Kristall- und Molekülstruktur von Bis(trimethylsilyl)diimin. *Acta Crystallogr. B* **1974**, *30* (7), 1806–1813. https://doi.org/10.1107/S056774087400584X

[S13] Lozinšek, M.; Mercier, H. P. A.; Schrobilgen G. J. Mixed Noble-Gas Compounds of Krypton(II) and Xenon(VI); $[F_5Xe(FKrF)AsF_6]$ and $[F_5Xe(FKrF)_2AsF_6]$. *Angew. Chem. Int. Ed.* **2021**, *60* (15), 8149–8156. https://doi.org/10.1002/anie.202014682

[S14] Rigaku OD. CrysAlis PRO. Rigaku Corporation, Wrocław, Poland, **2023**.

[S15] a) Palatinus, L.; Chapuis, G. *SUPERFLIP* – a computer program for the solution of crystal structures by charge flipping in arbitrary dimensions. *J. Appl. Crystallogr.* **2007**, *40* (4), 786–790. https://doi.org/10.1107/S0021889807029238

b) Palatinus, L.; van der Lee, A. Symmetry determination following structure solution in *P*1. *J. Appl. Crystallogr* **2008**, *41* (6), 975–984. https://doi.org/10.1107/S0021889808028185

c) Palatinus, L.; Prathapa, S. J.; van Smaalen, S. *EDMA*: a computer program for topological analysis of discrete electron densities. *J. Appl. Crystallogr.* **2012**, *45* (3), 575–580. https://doi.org/10.1107/S0021889812016068

[S16] Sheldrick, G. M. Crystal structure refinement with *SHELXL*. *Acta Crystallogr.* C **2015**, *71* (1), 3–8. https://doi.org/10.1107/S2053229614024218

[S17] Dolomanov, O. V.; Bourhis, L. J.; Gildea, R. J.; Howard, J. A. K.; Puschmann, H. *OLEX2*: a complete structure solution, refinement and analysis program. *J. Appl. Crystallogr.* **2009**, *42* (2), 339–341. https://doi.org/10.1107/S0021889808042726

[S18] Brandenburg, K. *Diamond* – Crystal and Molecular Structure Visualization, Crystal Impact GbR, Bonn, Germany, **2005**.

[S19] Putz, H. *Match!* – Phase Analysis using Powder Diffraction, Crystal Impact GbR, Bonn, Germany, **2023**.

[S20] Toby, B. H.; Von Dreele, R. B. *GSAS-II*: the genesis of a modern open-source all purpose crystallography software package. *J. Appl. Crystallogr.* **2013**, *46* (2), 544–549. https://doi.org/10.1107/S0021889813003531

[S21] Bohinc, M.; Grannec, J.; Slivnik, J.; Žemva, B. On the Syntheses of Xenon Fluoromanganates(IV). *J. Inorg. Nucl. Chem.* **1976**, *38* (1), 75–76. https://doi.org/10.1016/0022-1902(76)80052-8

[S22] Giannozzi, P.; Baroni, S.; Bonini, N.; Calandra, M.; Car, R.; Cavazzoni, C.; Ceresoli, D.; Chiarotti, G. L.; Cococcioni, M.; Dabo, I.; Dal Corso, A.; Gironcoli, S. de; Fabris, S.; Fratesi, G.; Gebauer, R.;

Gerstmann, U.; Gougoussis, C.; Kokalj, A.; Lazzeri, M.; Martin-Samos, L.; Marzari, N.; Mauri, F.; Mazzarello, R.; Paolini, S.; Pasquarello, A.; Paulatto, L.; Sbraccia, C.; Scandolo, S.; Sclauzero, G.; Seitsonen, A. P.; Smogunov, A.; Umari, P.; Wentzcovitch, R. M. QUANTUM ESPRESSO: A Modular and Open-Source Software Project for Quantum Simulations of Materials. *J. Phys: Condens. Matter* **2009**, *21* (39), 395502. https://doi.org/10.1088/0953-8984/21/39/395502

[S23] Giannozzi, P.; Andreussi, O.; Brumme, T.; Bunau, O.; Nardelli, M. B.; Calandra, M.; Car, R.; Cavazzoni, C.; Ceresoli, D.; Cococcioni, M.; Colonna, N.; Carnimeo, I.; Corso, A. D.; Gironcoli, S. de; Delugas, P.; DiStasio Jr, R. A.; Ferretti, A.; Floris, A.; Fratesi, G.; Fugallo, G.; Gebauer, R.; Gerstmann, U.; Giustino, F.; Gorni, T.; Jia, J.; Kawamura, M.; Ko, H.-Y.; Kokalj, A.; Küçükbenli, E.; Lazzeri, M.; Marsili, M.; Marzari, N.; Mauri, F.; Nguyen, N. L.; Nguyen, H.-V.; Otero-de-la-Roza, A.; Paulatto, L.; Poncé, S.; Rocca, D.; Sabatini, R.; Santra, B.; Schlipf, M.; Seitsonen, A. P.; Smogunov, A.; Timrov, I.; Thonhauser, T.; Umari, P.; Vast, N.; Wu, X.; Baroni, S. Advanced Capabilities for Materials Modelling with Quantum ESPRESSO. *J. Phys. Condens. Matter* **2017**, *29* (46), 465901. https://doi.org/10.1088/1361-648X/aa8f79

[S24] Perdew, J. P.; Burke, K.; Ernzerhof, M. Generalized Gradient Approximation Made Simple. *Phys. Rev. Lett.* **1996**, *77* (18), 3865–3868. https://doi.org/10.1103/PhysRevLett.77.3865

[S25] Grimme, S. Semiempirical GGA-Type Density Functional Constructed with a Long-Range Dispersion Correction. *J. Comput. Chem.* **2006**, *27* (15), 1787–1799. https://doi.org/10.1002/jcc.20495

[S26] Blöchl, P. E. Projector Augmented-Wave Method. *Phys. Rev. B* **1994**, *50* (24), 17953–17979. https://doi.org/10.1103/PhysRevB.50.17953

[S27] Dal Corso, A. Pseudopotentials Periodic Table: From H to Pu. *Comput. Mat. Sci.* **2014**, *95*, 337–350. https://doi.org/10.1016/j.commatsci.2014.07.043 Code available from: https://dalcorso.github.io/pslibrary/ (2024)

[S28] Baroni, S.; de Gironcoli, S.; Dal Corso, A.; Giannozzi, P. Phonons and Related Crystal Properties from Density-Functional Perturbation Theory. *Rev. Mod. Phys.* **2001**, *73* (2), 515–562. https://doi.org/10.1103/RevModPhys.73.515

[S29] Tang, W., Sanville, E.; Henkelman, G. A Grid-Based Bader Analysis Algorithm without Lattice Bias. *J. Phys: Condens. Matter* **2009**, *21* (8), 084204. https://doi.org/10.1088/0953-8984/21/8/084204

[S30] Arnaldsson, A.; Tang, W.; Henkelman, G.; et al. Computer Program for Bader Charge Analysis; http://theory.cm.utexas.edu/henkelman/code/bader/ (2024)

[S31] Kokalj, A. XCrySDen–a New Program for Displaying Crystalline Structures and Electron Densities. *J. Mol. Graph. Model.* **1999**, *17* (3–4), 176–179. https://doi.org/10.1016/S1093-3263(99)00028-5 Code available from: http://www.xcrysden.org/ (2024)
